# A Multilocus Phylogeny of the World Sycoecinae Fig Wasps (Chalcidoidea: Pteromalidae)

**DOI:** 10.1371/journal.pone.0079291

**Published:** 2013-11-05

**Authors:** Astrid Cruaud, Jenny G. Underhill, Maïlis Huguin, Gwenaëlle Genson, Roula Jabbour-Zahab, Krystal A. Tolley, Jean-Yves Rasplus, Simon van Noort

**Affiliations:** 1 INRA, UMR1062 CBGP Centre de Biologie pour la Gestion des Populations, Montferrier-sur-Lez, France; 2 South African National Biodiversity Institute, Kirstenbosch Research Centre, Cape Town, South Africa; 3 Natural History Division, South African Museum, Iziko Museums of Cape Town, Cape Town, South Africa; 4 Department of Zoology, University of Cape Town, Rondebosch, South Africa; Consiglio Nazionale delle Ricerche (CNR), Italy

## Abstract

The Sycoecinae is one of five chalcid subfamilies of fig wasps that are mostly dependent on *Ficus* inflorescences for reproduction. Here, we analysed two mitochondrial (*COI*, Cyt*b*) and four nuclear genes (ITS2, *EF-1α, RpL27a, mago nashi*) from a worldwide sample of 56 sycoecine species. Various alignment and partitioning strategies were used to test the stability of major clades. All topologies estimated using maximum likelihood and Bayesian methods were similar and well resolved but did not support the existing classification. A high degree of morphological convergence was highlighted and several species appeared best described as species complexes. We therefore proposed a new classification for the subfamily. Our analyses revealed several cases of probable speciation on the same host trees (up to 8 closely related species on one single tree of *F. sumatrana*), which raises the question of how resource partitioning occurs to avoid competitive exclusion. Comparisons of our results with fig phylogenies showed that, despite sycoecines being internally ovipositing wasps host-switches are common incidents in their evolutionary history. Finally, by studying the evolutionary properties of the markers we used and profiling their phylogenetic informativeness, we predicted their utility for resolving phylogenetic relationships of Chalcidoidea at various taxonomic levels.

## Introduction

Fig trees (*Ficus*, Moraceae) and their pollinating fig wasps (Agaonidae, Chalcidoidea, Hymenoptera) present a specialized case of an obligate pollination mutualism [1]. Each *Ficus* species is reliant on agaonid fig wasps for pollination and, in return, the pollinating fig wasp depends on its host *Ficus* for reproduction and larval development [2,3]. The fig - pollinator association is exploited by several other lineages of Chalcidoidea, a few Braconidae (Ichneumonoidea) and up to 30 fig wasp species can be associated with a single *Ficus* host [4,5]. Among these wasp lineages are the Sycoecinae (Chalcidoidea), a group that does not belong to Agaonidae as previously stated [6], but is clearly a member of the Pteromalidae *sensu stricto* [7–10].

In contrast to most other wasp lineages associated with figs, all the Sycoecinae are internal ovipositors. Sycoecines, which do not show any pollination behaviour, cannot pollinate actively pollinated *Ficus* species [11]. In passively pollinated *Ficus* species, large quantities of pollen are released by anther dehiscence so that all the emerging wasps become covered with pollen [12]. Therefore, sycoecines (*Diaziella*) associated with passively pollinated fig trees (*Conosycea* stranglers) are efficient pollinators [13].

Convergent evolution is believed to account for the morphological similarity between the sycoecines and pollinating fig wasps both being exposed to identical selection pressures due to the constraints of internal oviposition [14]. These morphological adaptations, such as smooth, elongated and dorso-ventrally flattened head and thorax, and the presence of tibial and mandibular modifications, enable both pollinators and sycoecines to crawl through tightly appressed bracts surrounding the ostiole to enter the fig cavity.

Presently, the Sycoecinae comprises six genera and 72 described species. The current taxonomy is based on morphological delimitation [15–19] but no molecular phylogenetic analysis has been attempted so far. Therefore our understanding of sycoecine evolutionary relationships is wanting. Diagnostic characters for the six sycoecine genera are provided in Table 1, for identification keys and detailed taxonomy, the reader is directed to the website www.figweb.org [20]. Four sycoecine genera, *Crossogaster* Mayr (16 described species), *Philocaenus* Grandi (24), *Sycoecus* Waterston (10) and *Seres* Waterston (4), are restricted to the Afrotropical region and are associated with *Ficus* section *Galoglychia* (Table 1, detailed distribution available at www.figweb.org, [20]. The genus *Diaziella* Grandi (14 described species) occurs in the Oriental region and is associated with *Ficus* section *Urostigma*, subsection *Conosycea* [21–23]. Finally, the New Guinean genus *Robertsia* Bouček (4 described species) is associated with *Ficus* section *Stilpnophyllum*, subsection *Malvanthera* (Table 1) [6,24]. Extrapolating from our sampling of several hundred species of *Ficus* the total diversity of the Sycoecinae could reach 190 species worldwide. The subfamily is better known from the Afrotropical region (where an estimated 63% of the species are described) than the Oriental and Indo-Australasian regions where only an estimated 10% of the species are described [20]. Consequently, and despite that the Sycoecinae is one of the better-known fig wasp subfamilies, overall more than 65 % of the species await description. 

**Table 1:** Type species, diversity, distribution, host fig tree groups and diagnostic characters for the six previously defined genera of Sycoecinae.

**Genera**	**Type species**	**Species described**	**Distribution**	**Host *Ficus*subgenus/*section*/** *subsection*	**Diagnosis**
*Crossogaster* Mayr, 1885	*C. triformis* Mayr, 1885	16	Afrotropical	**Urostigma** ***Galoglychia** Platyphyllae*, *Chlamydodorae*, *Crassicostae*, *Caulocarpae*	Both sexes with one labial palp segment and two maxillary palp segments; the female eighth urotergite spiracular peritremata distinctly expanded; the male inner apical mandibular tooth is subequal (but still longer) to much longer than the outer tooth [17]
*Diaziella* Grandi, 1928	*D. bicolor* Grandi, 1928	14	Oriental	**Urostigma** **Urostigma** *Conosycea*	Four-segmented fore tarsi; a laminar projection present on the proximal fore tarsal segment; a pronounced hypopygium that may extend well beyond the end of the metasoma; an ovipositor usually long (about the length of the metasoma) sometimes shorter [22].
*Philocaenus* Grandi, 1952	*P. barbatus* Grandi, 1952	24	Afrotropical	***Urostigma Galoglychia** Galoglychia, Platyphyllae, Chlamydodorae, Caulocarpae*	Females with two anelli and four funicle segments; gastral tergites with a crenulated posterior edge; ventral tentorial pits in close apposition; eighth urotergite spiracle not expanded. Males have the outer mandibular tooth longer than the inner, without any ventral teeth present [18,19].
*Robertsia* Bouček, 1988	*R. mandibularis* Bouček, 1988	4	New Guinean	**Urostigma** ***Stilpnophyllum** Malvanthera*	No antennal scrobe; clypeal sutures not defined; medial carina present between the toruli; tarsi five-segmented; hypopygium not extending beyond end of metasoma; ovipositor short, usually not discernable [24].
*Seres* Waterston, 1919	*S. armipes* Waterston, 1919	4	Afrotropical	**Urostigma** ***Galoglychia** Caulocarpae*	Propodeal spiracles medially positioned with a plica extending from the internal edge of the spiracle to the posterior margin of the propodeum, which may sometimes be indistinct [15].
*Sycoecus* Waterston, 1914	*S. thaumastocnema* Waterston, 1914	10	Afrotropical	**Urostigma** ***Galoglychia** Cyathistipulae*	Fore tibial spur modified into a plate of many fine teeth; first funicle segment with axial expansion; propleura excavated; pronotum with distinct lateral depressions [16].

Classification of the genus *Ficus* follows Berg and Corner [93].

How fig wasp communities have been structured over space and time is a fascinating question that remains to be answered [1]. However, before addressing issues such as “how can we explain species coexistence in these closed communities?”, “do the partners have the same biogeographical history?” and “do we observe cophylogenetic patterns between partners?”, we first need robust phylogenies validated by expert taxonomists for all the involved fig-wasp lineages.

In this paper we employ multiple genetic loci, extensive taxon sampling of both described and undescribed species and several different analytical approaches to 1) evaluate the monophyly of the sycoecine genera, 2) provide a first comprehensive phylogenetic hypothesis for the subfamily, 3) propose a new classification and 4) discuss the phylogenetic relationships in light of the host fig tree relationships. 

On a more general note, we were interested in testing a combination of frequently used and recently developed markers to resolve the phylogenetic relationships of chalcidoid lineages at various taxonomic levels. Chalcidoidea have tremendous importance in both natural and managed ecosystems. Several species are for example used as biological control agents of agricultural and ornamental pests [25]. However, our understanding of how many of these lineages are monophyletic and what are their phylogenetic relationships is clearly wanting [9,10]. Although genes should be selected to match the time period of the phylogenetic problem at hand [26,27], the most commonly used markers remain 28S and 18S ribosomal genes, Cytochrome oxidase I (COI) and Cytochrome b (*Cytb*), whatever the taxonomic level studied (e.g. [9,28–33]). While ribosomal genes often evolve too slowly to resolve relationships in rapidly diversifying lineages, mitochondrial genes become saturated over larger time scales and are poor estimators of a phylogeny at high taxonomic level [26,34]. By studying the evolutionary properties of the markers we sequenced, profiling their phylogenetic informativeness (sensu Townsend [27]) under different alignment procedures, and comparing inferred topologies, we predict their utility for phylogenetic inference at shallower and deeper taxonomic levels. 

## Materials and Methods

### 1. Taxonomic sampling

**Figure 1:**
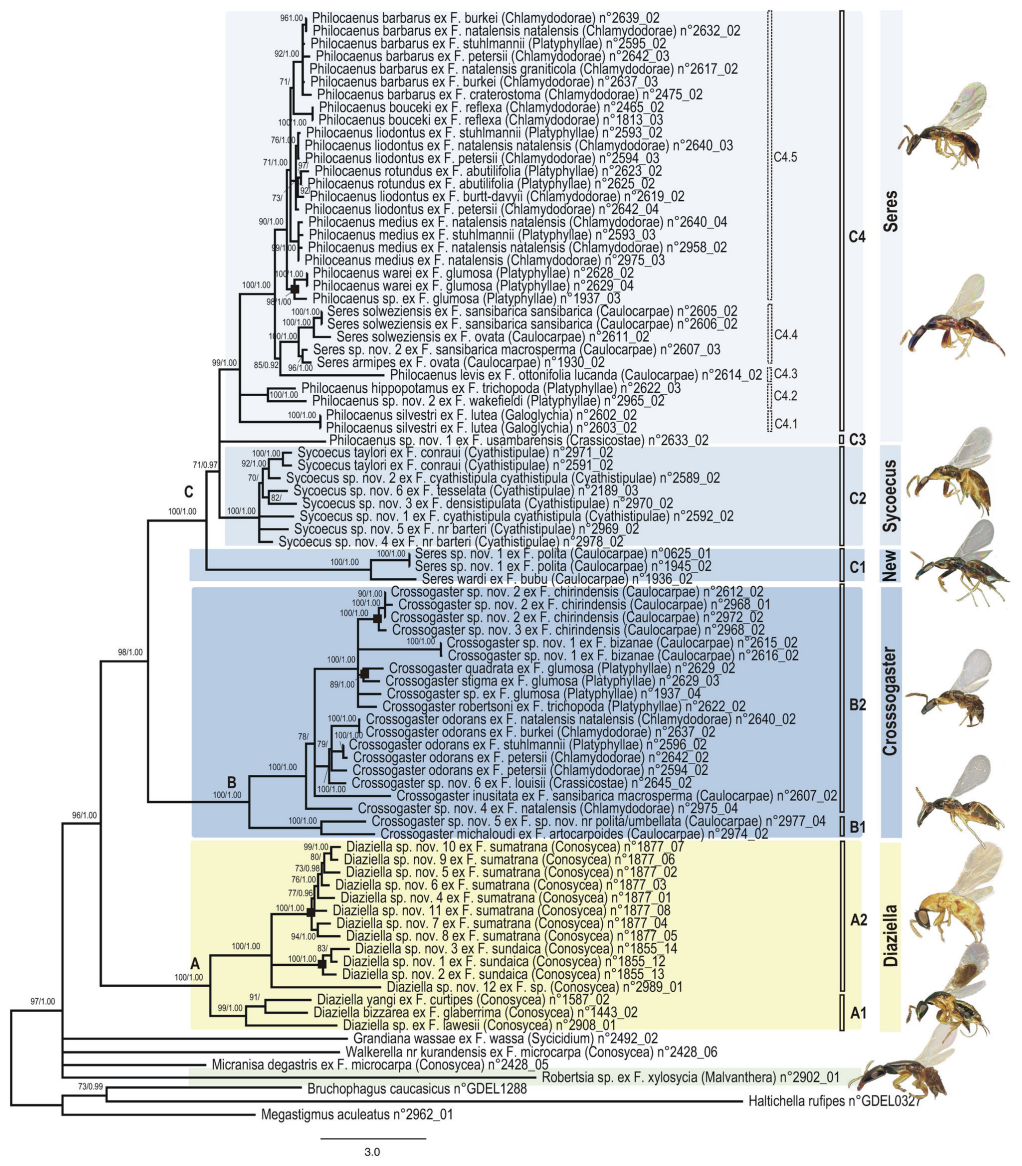
Phylogram of relationships among the Sycoecinae obtained from the analysis of the MAFFT alignment (combined dataset, without Gblocks cleaning, 6 partitions: mtDNAcodon1&2, mtDNAcodon3, *EF-1α*, ITS2, *RpL27a*, *mago* *nashi*). Uppercase letters refer to clades discussed in the text. The new classification is indicated by colored bars on the right (yellow = oriental species, blue = afrotropical species). Nodes with likelihood bootstrap (BP) values < 70 have been collapsed. BP (> 70) and Bayesian posterior probabilities (> 0.90) are indicated at nodes. Illustrations of female habitus for the main clades are provided on the right. Host fig tree subsections are indicated between parentheses. Black boxes at nodes show cases of probable speciation on a single host *Ficus* species.

Species identification was based on morphological characteristics according to the current literature and was conducted by SvN and JYR. Morphological observations were performed on 10 to 20 representatives of each species. A total of 81 sycoecine specimens representing all the known genera and 56 species, of which 50% are undescribed, were included in the molecular study (Table 2). Seventy percent of the species were represented by sequences from one specimen only. The type-species of the genera *Seres* (*S. armipes*) and *Philocaenus* (*P. barbarus*) were also included in our analyses. All necessary permits were obtained for the collection of specimens in nature reserves and national parks (Ministère des Eaux et Forêts et du Reboisement, Libreville, Gabon permit granted by Emile Mamfoumbi Kombila, Directeur de la Faune et de la Chasse; Uganda Wildlife Authority UWA/TDO/33/02; UNCST NS 214; Namibia Ministry of Environment and Tourism permit 1289/2008; Ezemvelo KZN Wildlife permits 2985/1999; 24139/2000; 29820/2002; 4502/2005; 4345/2005; 4346/2005;1958/2007; Kenyan Wildlife Service KWS/RP/5001; Cape Nature permits 288/1999; AAA004-00092-0035; AAA007-00324-0035; Northern Cape Province permits Fauna 131/2010, Fauna 132/2010; Eastern Cape Province permits CRO 101/11CR and CRO 102/11CR; Permits for field work in Borneo were obtained from the Sarawak Forestry Corporation and the Forest Research Institute Malaysia; Permits for specimen collection in China were obtained from the Chinese Natural Science Foundation (30670358, 30571507), KSCX2-YW-Z-003; Permits for the collection of specimen in Sulawesi were obtained from the Research Center for Biology LIPI (No : 3180/SU.3/KS/2007); Specimen collection in Taiwan was funded by the ANR project BioFigs/National Science Council, Taiwan, R. O. C. code: 98WFA0100291). Wasps were collected by sampling figs containing either adults or developing wasp larvae. The figs, containing wasps no more than a few days short of their emergence, were placed in handmade wasp-rearing chambers. Once emerged, adult wasps were killed and preserved in 95% ethanol. While the relationships between the chalcidoid subfamilies remain controversial, closer and more distant relatives were included as outgroups [9,10,35]. Six species belonging to the genera *Haltichella* (Haltichellinae, Chalcididae), *Bruchophagus* (Eurytominae, Eurytomidae), *Grandiana* (Otitesellinae, Pteromalidae), *Micranisa* (Otitesellinae, Pteromalidae), *Walkerella* (Otitesellinae, Pteromalidae), and *Megastigmus* (Megastigminae, Torymidae) were used (Table 2). Each time destructive extraction was used, vouchers were selected among specimens sampled from the same fig tree and the same fig after careful identification. Vouchers are deposited at CBGP, Montferrier-sur-Lez, France and Iziko South African Museum, Cape Town (SAMC). A high definition image library of vouchers was also constructed, using the EntoVision Premium Portable Imaging System, to allow future identification of specific taxa and traceability of our results (see Figure 1 for examples).

**Table 2:** List of Sycoecinae and outgroup species included in this study: voucher numbers and depository, taxonomic information, host *Ficus* species and locality data.

**Family**	**Subfamily**	**Genus**	**Species**	**Voucher number**	**Host *Ficus* species**	**host *Ficus* section**	**Locality**
Pteromalidae	*Sycoecinae*	*Crossogaster*	*inusitata*	2607_02 (SAMC)	*F. sansibarica macrosperma*	*Caulocarpae*	Zambia, West Kawambwa
	*Sycoecinae*	*Crossogaster*	*michaloudi*	2974_02 (SAMC)	*F. artocarpoides*	*Caulocarpae*	Uganda, Kibale National Park
	*Sycoecinae*	*Crossogaster*	*odorans*	2594_02 (SAMC)	*F. petersii*	*Chlamydodorae*	South Africa, Mapumulanga
	*Sycoecinae*	*Crossogaster*	*odorans*	2637_02 (SAMC)	*F. burkei*	*Chlamydodorae*	South Africa, Abel Erasmus Pass
	*Sycoecinae*	*Crossogaster*	*odorans*	2640_02 (SAMC)	*F. natalensis natalensis*	*Chlamydodorae*	Zambia, Kapiri Mposhi
	*Sycoecinae*	*Crossogaster*	*odorans*	2642_02 (SAMC)	*F. petersii*	*Chlamydodorae*	Zambia, Southeast Isoka
	*Sycoecinae*	*Crossogaster*	*quadrata*	2629_02 (SAMC)	*F. glumosa*	*Platyphyllae*	South Africa, Port Edward
	*Sycoecinae*	*Crossogaster*	*robertsoni*	2622_02 (SAMC)	*F. trichopoda*	*Platyphyllae*	South Africa, Umlalazi
	*Sycoecinae*	*Crossogaster*	sp.	1937_04 (CBGP)	*F. glumosa*	*Platyphyllae*	Cameroun, Tibati
	*Sycoecinae*	*Crossogaster*	sp. nov. 1	2615_02 (SAMC)	*F. bizanae*	*Caulocarpae*	South Africa, Ongoye forest
	*Sycoecinae*	*Crossogaster*	sp. nov. 1	2616_02 (SAMC)	*F. bizanae*	*Caulocarpae*	South Africa, Port St Johns
	*Sycoecinae*	*Crossogaster*	sp. nov. 2	2612_02 (SAMC)	*F. chirindensis*	*Caulocarpae*	Uganda, Kibale National Park
	*Sycoecinae*	*Crossogaster*	sp. nov. 2	2968_01 (SAMC)	*F. chirindensis*	*Caulocarpae*	Kenya, Kakemega Forest
	*Sycoecinae*	*Crossogaster*	sp. nov. 2	2972_02 (SAMC)	*F. chirindensis*	*Caulocarpae*	Uganda, Kibale National Park
	*Sycoecinae*	*Crossogaster*	sp. nov. 3	2968_02 (SAMC)	*F. chirindensis*	*Caulocarpae*	Kenya, Kakemega Forest
	*Sycoecinae*	*Crossogaster*	sp. nov. 4	2975_04 (SAMC)	*F. natalensis*	*Chlamydodorae*	Uganda, Kibale National Park
	*Sycoecinae*	*Crossogaster*	sp. nov. 5	2977_04 (SAMC)	F. sp. nov. nr *polita/umbellata*	*Caulocarpae*	Uganda, Kibale National Park
	*Sycoecinae*	*Crossogaster*	sp. nov. 6	2645_02 (SAMC)	*F. louisii*	*Crassicostae*	Gabon, Mt Doudou
	*Sycoecinae*	*Crossogaster*	*stigma*	2629_03 (SAMC)	*F. glumosa*	*Platyphyllae*	South Africa, Port Edward
	*Sycoecinae*	*Crossogaster*	*odorans*	2596_02 (SAMC)	*F. stuhlmannii*	*Platyphyllae*	South Africa, Hluhluwe region
	*Sycoecinae*	*Diaziella*	*bizzarea*	1443_02 (CBGP)	*F. glaberrima*	*Conosycea*	China, Yunnan, XTGB
	*Sycoecinae*	*Diaziella*	sp.	2908_01 (CBGP)	*F. lawesii*	*Conosycea*	Indonesia, Sulawesi, South Buton
	*Sycoecinae*	*Diaziella*	sp. nov. 1	1855_12 (CBGP)	*F. sundaica*	*Conosycea*	Malaysia, Sarawak
	*Sycoecinae*	*Diaziella*	sp. nov. 10	1877_07 (CBGP)	*F. sumatrana*	*Conosycea*	Malaysia, Sarawak
	*Sycoecinae*	*Diaziella*	sp. nov. 11	1877_08 (CBGP)	*F. sumatrana*	*Conosycea*	Malaysia, Sarawak
	*Sycoecinae*	*Diaziella*	sp. nov. 12	2989_01 (CBGP)	F. sp.	*Conosycea*	Philippines, Luzon, Mt Makiling, Los Banos
	*Sycoecinae*	*Diaziella*	sp. nov. 2	1855_13 (CBGP)	*F. sundaica*	*Conosycea*	Malaysia, Sarawak
	*Sycoecinae*	*Diaziella*	sp. nov. 3	1855_14 (CBGP)	*F. sundaica*	*Conosycea*	Malaysia, Sarawak
	*Sycoecinae*	*Diaziella*	sp. nov. 4	1877_01 (CBGP)	*F. sumatrana*	*Conosycea*	Malaysia, Sarawak
	*Sycoecinae*	*Diaziella*	sp. nov. 5	1877_02 (CBGP)	*F. sumatrana*	*Conosycea*	Malaysia, Sarawak
	*Sycoecinae*	*Diaziella*	sp. nov. 6	1877_03 (CBGP)	*F. sumatrana*	*Conosycea*	Malaysia, Sarawak
	*Sycoecinae*	*Diaziella*	sp. nov. 7	1877_04 (CBGP)	*F. sumatrana*	*Conosycea*	Malaysia, Sarawak
**Family**	**Subfamily**	**Genus**	**Species**	**Voucher number**	**Host *Ficus* species**	**host Ficus section**	**Locality**
	*Sycoecinae*	*Diaziella*	sp. nov. 8	1877_05 (CBGP)	*F. sumatrana*	*Conosycea*	Malaysia, Sarawak
	*Sycoecinae*	*Diaziella*	sp. nov. 9	1877_06 (CBGP)	*F. sumatrana*	*Conosycea*	Malaysia, Sarawak
	*Sycoecinae*	*Diaziella*	*yangi*	1587_02 (CBGP)	*F. curtipes*	*Conosycea*	China, Yunnan, Lancon
	*Otitesellinae*	*Micranisa*	*degastris*	2428_05 (CBGP)	*F. microcarpa*	*Conosycea*	Taiwan, Shiti
	*Sycoecinae*	*Philocaenus*	*barbarus*	2475_02 (SAMC)	*F. craterostoma*	*Chlamydodorae*	South Africa, Ngome forest
	*Sycoecinae*	*Philocaenus*	*barbarus*	2595_02 (SAMC)	*F. stuhlmannii*	*Platyphyllae*	South Africa, False Bay Park
	*Sycoecinae*	*Philocaenus*	*barbarus*	2617_02 (SAMC)	*F. natalensis graniticola*	*Chlamydodorae*	South Africa, Soutpansberg
	*Sycoecinae*	*Philocaenus*	*barbarus*	2632_02 (SAMC)	*F. natalensis natalensis*	*Chlamydodorae*	South Africa, Mtunzini
	*Sycoecinae*	*Philocaenus*	*barbarus*	2637_03 (SAMC)	*F. burkei*	*Chlamydodorae*	South Africa, Abel Erasmus Pass
	*Sycoecinae*	*Philocaenus*	*barbarus*	2639_02 (SAMC)	*F. burkei*	*Chlamydodorae*	South Africa, Port Edward
	*Sycoecinae*	*Philocaenus*	*barbarus*	2642_03 (SAMC)	*F. petersii*	*Chlamydodorae*	Zambia, Southeast Isoka
	*Sycoecinae*	*Philocaenus*	*bouceki*	1813_03 (CBGP)	*F. reflexa*	*Chlamydodorae*	Madagascar, Ranomafana
	*Sycoecinae*	*Philocaenus*	*bouceki*	2465_02 (CBGP)	*F. reflexa*	*Chlamydodorae*	Madagascar, Ranomafana
	*Sycoecinae*	*Philocaenus*	*hippopotamus*	2622_03 (SAMC)	*F. trichopoda*	*Platyphyllae*	South Africa, Umlalazi
	*Sycoecinae*	*Philocaenus*	*levis*	2614_02 (SAMC)	*F. ottonifolia lucanda*	*Caulocarpae*	Uganda, Kibale National Park
	*Sycoecinae*	*Philocaenus*	*liodontus*	2593_02 (SAMC)	*F. stuhlmannii*	*Platyphyllae*	Mozambique, Mandimba
	*Sycoecinae*	*Philocaenus*	*liodontus*	2619_02 (SAMC)	*F. burtt-davyii*	*Chlamydodorae*	South Africa, Woody Cape Reserve
	*Sycoecinae*	*Philocaenus*	*liodontus*	2640_03 (SAMC)	*F. natalensis natalensis*	*Chlamydodorae*	Zambia, Kapiri Mposhi
	*Sycoecinae*	*Philocaenus*	*liodontus*	2642_04 (SAMC)	*F. petersii*	*Chlamydodorae*	Zambia, Southeast Isoka
	*Sycoecinae*	*Philocaenus*	*medius*	2593_03 (SAMC)	*F. stuhlmannii*	*Platyphyllae*	Mozambique, Mandimba
	*Sycoecinae*	*Philocaenus*	*medius*	2640_04 (SAMC)	*F. natalensis natalensis*	*Chlamydodorae*	Zambia, Kapiri Mposhi
	*Sycoecinae*	*Philocaenus*	*medius*	2958_02 (SAMC)	*F. natalensis natalensis*	*Chlamydodorae*	Mozambique, South Nhachengue
	*Sycoecinae*	*Philocaenus*	*rotundus*	2623_02 (SAMC)	*F. abutilifolia*	*Platyphyllae*	South Africa, Kwazulu-Natal, Jozini
	*Sycoecinae*	*Philocaenus*	*rotundus*	2625_02 (SAMC)	*F. abutilifolia*	*Platyphyllae*	South Africa, Soutpansberg
	*Sycoecinae*	*Philocaenus*	*silvestrii*	2602_02 (SAMC)	*F. lutea*	*Galoglychia*	South Africa, Makhado
	*Sycoecinae*	*Philocaenus*	*silvestrii*	2603_02 (SAMC)	*F. lutea*	*Galoglychia*	South Africa, Ongoye forest
	*Sycoecinae*	*Philocaenus*	sp.	1937_03 (CBGP)	*F. glumosa*	*Platyphyllae*	Cameroun, Tibati
	*Sycoecinae*	*Philocaenus*	sp. nov. 1	2633_02 (SAMC)	*F. usambarensis*	*Crassicostae*	Zambia, Southwest Mporokoso
	*Sycoecinae*	*Philocaenus*	sp. nov. 2	2965_02 (SAMC)	*F. wakefieldi*	*Platyphyllae*	Kenya, SW Nairobi
	*Sycoecinae*	*Philocaenus*	*warei*	2628_02 (SAMC)	*F. glumosa*	*Platyphyllae*	South Africa, Makhado
	*Sycoecinae*	*Philocaenus*	*warei*	2629_04 (SAMC)	*F. glumosa*	*Platyphyllae*	South Africa, Port Edward
	*Sycoecinae*	*Philocaenus*	*liodontus*	2594_03 (SAMC)	*F. petersii*	*Chlamydodorae*	South Africa, Mapumulanga
	*Sycoecinae*	*Philoceanus*	*medius*	2975_03 (SAMC)	*F. natalensis*	*Chlamydodorae*	Uganda, Kibale National Park
**Family**	**Subfamily**	**Genus**	**Species**	**Voucher number**	**Host *Ficus* species**	**host Ficus section**	**Locality**
	*Sycoecinae*	*Robertsia*	sp.	2902_01 (CBGP)	*F. xylosycia*	*Malvanthera*	Papua New Guinea, East New Britain, Raunsepna
	*Sycoecinae*	*Seres*	*armipes*	1930_02 (CBGP)	*F. ovata*	*Caulocarpae*	Cameroun, West Baha
	*Sycoecinae*	*Seres*	*solweziensis*	2605_02 (SAMC)	*F. sansibarica sansibarica*	*Caulocarpae*	South Africa, Legalameetse Nature Reserve
	*Sycoecinae*	*Seres*	*solweziensis*	2606_02 (SAMC)	*F. sansibarica sansibarica*	*Caulocarpae*	South Africa, Mpumalanga
	*Sycoecinae*	*Seres*	*solweziensis*	2611_02 (SAMC)	*F. ovata*	*Caulocarpae*	Zambia, Kawambwa
	*Sycoecinae*	*Seres*	sp. nov. 1	0625_01 (CBGP)	*F. polita*	*Caulocarpae*	Madagascar, Joffreville
	*Sycoecinae*	*Seres*	sp. nov. 1	1945_02 (CBGP)	*F. polita*	*Caulocarpae*	Madagascar, Ambondanihefy
	*Sycoecinae*	*Seres*	sp. nov. 2	2607_03 (SAMC)	*F. sansibarica macrosperma*	*Caulocarpae*	Zambia, West Kawambwa
	*Sycoecinae*	*Seres*	*wardi*	1936_02 (CBGP)	*F. bubu*	*Caulocarpae*	Cameroun, South Ebolowa
	*Sycoecinae*	*Sycoecus*	sp. nov. 1	2592_02 (SAMC)	*F. cyathistipula cyathistipula*	*Cyathistipulae*	Tanzania, Lake Chala
	*Sycoecinae*	*Sycoecus*	sp. nov. 2	2589_02 (SAMC)	*F. cyathistipula cyathistipula*	*Cyathistipulae*	Mozambique, Mount Namuli
	*Sycoecinae*	*Sycoecus*	sp. nov. 3	2970_02 (SAMC)	*F. densistipulata*	*Cyathistipulae*	Uganda, Mabira Forest
	*Sycoecinae*	*Sycoecus*	sp. nov. 4	2978_02 (SAMC)	*F*. nr *barteri*	*Cyathistipulae*	Zambia, Ikalenge
	*Sycoecinae*	*Sycoecus*	sp. nov. 5	2969_02 (SAMC)	*F*. nr *barteri*	*Cyathistipulae*	Uganda, Mabira Forest
	*Sycoecinae*	*Sycoecus*	sp. nov. 6	2189_03 (SAMC)	*F. tesselata*	*Cyathistipulae*	Gabon, Makokou
	*Sycoecinae*	*Sycoecus*	*taylori*	2591_02 (SAMC)	*F. conraui*	*Cyathistipulae*	Uganda, Kibale National Park
	*Sycoecinae*	*Sycoecus*	*taylori*	2971_02 (SAMC)	*F. conraui*	*Cyathistipulae*	Uganda, Kibale National Park
	*Otitesellinae*	*Walkerella*	*nr kurandensis*	2428_06 (CBGP)	*F. microcarpa*	*Conosycea*	Taiwan, Shiti
	*Otitesellinae*	*Grandiana*	*wassae*	2492_02 (CBGP)	*F. wassa*	*Sycicidium*	Solomon Islands, Guadalcanal
Chalcididae	*Haltichellinae*	*Haltichella*	*rufipes*	GDEL0327 (CBGP)	N/A	N/A	France, Alpes-Maritimes, Vallon Gordolasque
Eurytomidae	*Eurytominae*	*Bruchophagus*	*caucasicus*	GDEL1288 (CBGP)	N/A	N/A	France, Alpes-Maritimes, Lucéram, Mont L’Ablé
Torymidae	*Megastigminae*	*Megastigmus*	*aculeatus*	2962_01 (SAMC)	N/A	N/A	Tanzania, SW Kalumbo

More information is available from the authors upon request.

### 2. Marker choice, DNA extraction, PCR amplification and sequencing

We selected markers that retain phylogenetic signal within deeper nodes as well as within terminal clades. We combined two mitochondrial protein-coding genes (Cytochrome oxydase I, COI and Cytochrome b, Cyt*b*) and four nuclear genes (the F2 copy of elongation factor-1a, *EF-1α*; the internal transcribed spacer 2, ITS2; the ribosomal protein *RpL27a* and the regulatory protein *mago nashi*). 

mtDNA loci and ITS2 have proven useful in resolving insect molecular phylogenies at shallower taxonomic levels. However, they are rapidly evolving, which make them poor markers for deep divergences [26,34]. *EF-1α* has been successfully used to resolve within-family relationships (e.g. [36–39]). While the exonic portions of *RpL27a* have proven informative for resolving deep-level relationships within the Hymenoptera [40], its intronic portions have been successfully used to reconstruct the phylogeography of parasitoids of oak galls (Pteromalidae, [41,42]. To our knowledge, *mago nashi* which encodes for a transcription factor that plays essential roles in *Drosophila axis* formation [43] has never been included in studies of insect molecular phylogeny.

Genomic fig wasp DNA was isolated using standard phenol–chloroform extraction and Qiagen DNeasy or ZyGEM extraction kits following standard protocols. Primer sequences and amplification protocols followed Cruaud et al. [44] for Cyt*b* and *COI*, Cruaud et al. [45] for *EF-1α* , Lopez-Vaamonde et al. [46] for ITS2 and Lohse et al. [41] for *RpL27a* and *mago nashi*. PCR products were purified using ExonucleaseI and Phosphatase, and sequenced directly using the BigDyeTerminator V3.1 kit (Applied Biosystems) and an ABI3730XL sequencer at Genoscope, Evry, France. Both strands for each overlapping fragment were assembled using the sequence editing software Geneious v5.5 [47]. All the sequences were deposited in GenBank (Table S1).

### 3. Phylogenetic analyses

Alignments of *COI*, Cyt*b*, *EF-1α* and *mago nashi* were straightforward due to a lack of length variation. ITS2 and *RPL27a* were aligned using various procedures, to assess the impact of alignment methods on phylogenetic inferences. ITS2 and *RPL27a* alignments were reconstructed with ClustalW [48] and MAFFT 6.864 [49]. Default parameters were used in ClustalW and the L-INS-i option was chosen in MAFFT [49]. Alignments were then cleaned from highly divergent blocks using the online version of Gblocks 0.91b [50] using the default settings and a less stringent selection of blocks. For the latter strategy (hereafter named « Gblocks relaxed »), the "minimum number of sequences for a flanking position" and the "minimum number of sequences for a conserved position" were set to half the number of sequences, the "minimum length of a block” was set to 5 and selection of positions with gaps present in less than half of the sequences was allowed.

Alignments of the protein coding genes were translated to amino acids using Mega 4.0.2 [51] to detect frameshift mutations and premature stop codons, which may indicate the presence of pseudogenes. Alignments of individual gene regions obtained with each strategy were first analysed separately to produce single-gene trees and then concatenated, resulting in six combined datasets (ClustalW; ClustalW + Gblocks default parameters; ClustalW + Gblocks relaxed parameters; MAFFT; MAFFT + Gblocks default parameters; MAFFT + Gblocks relaxed parameters). All combined datasets (including charsets) have been deposited on TreeBase (http://purl.org/phylo/treebase/phylows/study/TB2:S14838).

Phylogenetic trees of the six combined datasets were estimated using maximum likelihood (ML) and Bayesian methods. All analyses were conducted on a 150 cores Linux Cluster at CBGP as well as on the CIPRES Science Gateway [52]. Two alternative partitioning strategies were compared using Bayes factors (BF) [53]: Scheme 1: mitochondrial genes, *EF-1α*, ITS2, *RpL27a* and *mago nashi versus* Scheme 2: first + second codon positions of mitochondrial genes, third codon positions of mitochondrial genes, *EF-1α*, ITS2, *RpL27a* and *mago nashi*. Following Kass and Raftery [53], Pagel and Meade [54], and Schulte and de Queiroz [55], Bayes factors were calculated using the following formula: BF = 2*(ln L1 - ln L0) + (P1-P0) * ln (0.01) where ln Li and Pi are respectively, the harmonic mean of the ln likelihoods and the number of free parameters of model i. BF values from 2 to 6 were considered positive evidence, from 6 to 10 as strong evidence, and > 10 as very strong evidence favouring the alternative hypothesis over the null hypothesis. Best fitting model for each partition was identified using the Akaike information criterion [56] as implemented in MrAIC.pl 1.4.3 [57]. We performed ML analyses and associated bootstrapping using the MPI-parallelized RAxML 7.2.8-ALPHA [58]. GTRCAT approximation of models was used for ML boostrapping [58] (1000 replicates). Bootstrap percentage (BP) > 95% was considered as strong support and a BP < 70% as weak. 

**Figure 2:**
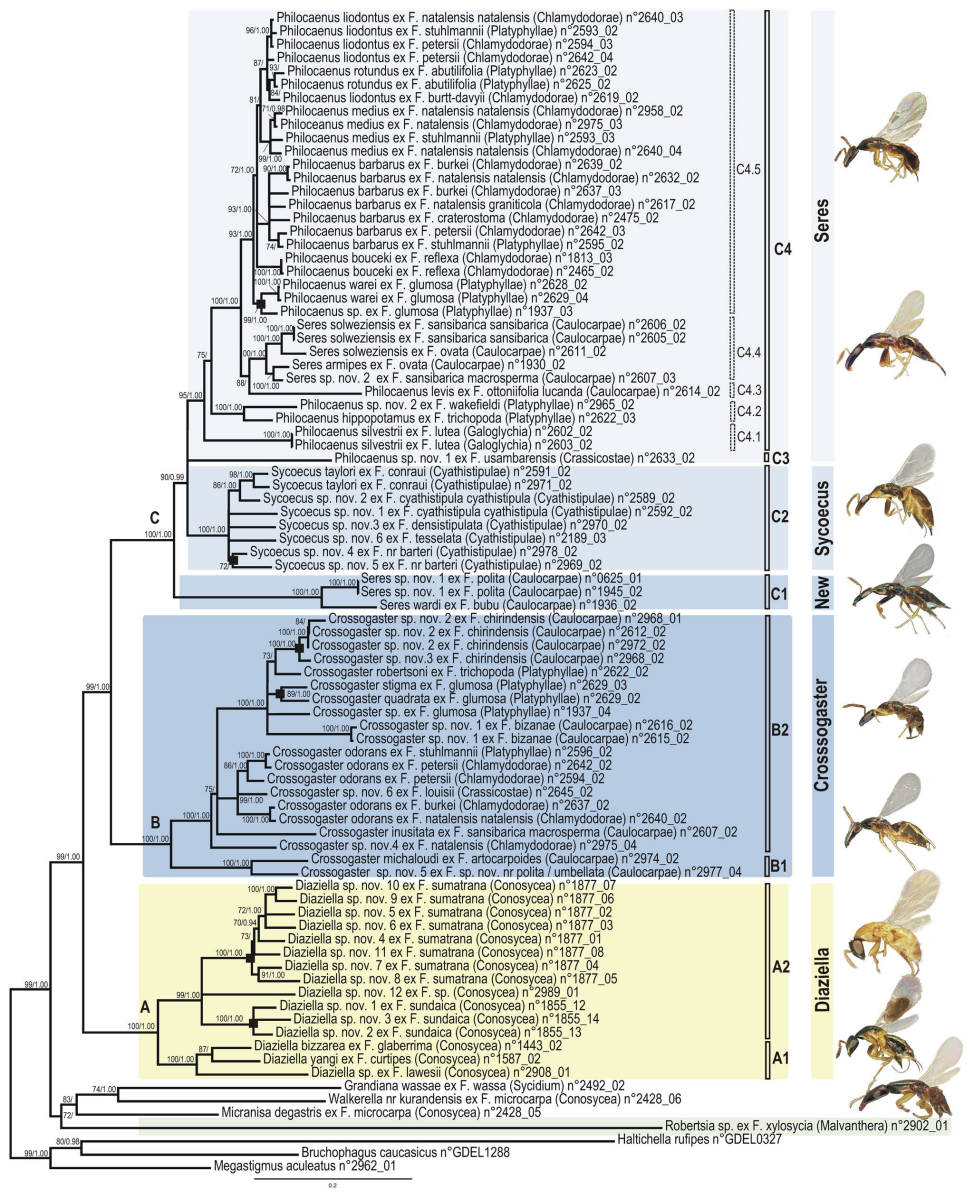
Phylogram of relationships among the Sycoecinae obtained from the analysis of the ClustalW alignment (combined dataset, without Gblocks cleaning, 5 partitions: mtDNA, *EF-1α*, ITS2, *RpL27a*, *mago* *nashi*). Uppercase letters refer to clades discussed in the text. The new classification is indicated by colored bars on the right (yellow = oriental species, blue = afrotropical species). Nodes with likelihood bootstrap values < 70 have been collapsed. BP (> 70) and Bayesian posterior probabilities (> 0.90) are indicated at nodes. Illustrations of female habitus for the main clades are provided on the right. Host fig tree subsections are indicated between parentheses. Black boxes at nodes show cases of probable speciation on a single host *Ficus* species.

Bayesian analyses were conducted using a parallel version of MrBayes v. 3.2.1 [59]. We assumed across-partition heterogeneity in model parameters by unlinking parameters across partitions. Parameter values for the model were initiated with default uniform priors and branch lengths were estimated using default exponential priors. To improve mixing of the cold chain and avoid it converging on local optima, we used Metropolis-coupled Markov chain Monte Carlo (MCMC), with each run including a cold chain and three incrementally heated chains. The heating parameter was set to 0.02 in order to allow swap frequencies from 20% to 70% [60]. We ran two independent runs of 20 million generations for the MAFFT-6 partitions and the MAFFT-Gblocks relaxed-6 partitions datasets and sampled values every 2000 generations. We ran two independent runs of 10 million generations for all other datasets and sampled values every 1000 generations. For the initial determination of burn-in, we examined the plot of overall model likelihood against generation number to find the point where the likelihood started to fluctuate around a constant value. The points sampled prior to convergence of the chains were then discarded. Convergence was also evaluated using the effective sample size (ESS) values of each parameters as reported in Tracer v1.5 [61]. The results were based on the pooled samples from the stationary phases of the two independent runs. Posterior probabilities (PP) > 0.95 were considered as strong support.

### 4. Test of alternative topologies

To compare topologies and assess whether certain alternative relationships among recovered clades could be statistically rejected, we performed AU [62] and SH [63] tests implemented in the program package CONSEL [64]. The program *makermt* was used to generates K=10 sets of bootstrap replicates. Each set consisted of 100,000 replicates of the row sums (10 times the default number of replicates). Default scales parameters were used (r_1_=0.5, r_2_=0.6, r_3_=0.7, r_4_=0.8, r_5_=0.9, r_6_=1, r_7_=1.1, r_8_=1.2 r_9_=1.3, r_10_=1.4), meaning that in the *k*-th set of replicates, *N*_k_ = *r*_k_ *N* sites were randomly chosen with replacement to calculate the row sums (with N=total number of sites). RAxML was used to compute the per-site log likelihoods for all trees.

### 5. Phylogenetic informativeness of markers

We initially compared the evolutionary properties of the markers using a Bayesian framework. For each partition of the complete combined datasets (without Gblocks cleaning), we studied base composition, substitution rates and rate variation among sites (α). We also compared rate variation among partitions, considering the parameter m (rate multiplier).

We profiled the phylogenetic informativeness (PI) of the markers, using the method described by Townsend [27] and implemented in the program PhyDesign [65]). This method uses per-site rate estimates to project the utility of a gene for resolving phylogeny across lineage history [27,66]. For each partition of a combined dataset, the phylogenetic informativeness of all sites can be summed, which provides the net phylogenetic informativeness (i.e. the degree to which the partition is predicted to contribute to resolution of the phylogeny across history). The informativeness can also be divided by the number of sites, resulting in the phylogenetic informativeness per site. We adopted this later approach in the present study as it allows a comparison of the relative power of genes without the influence of gene length, which may be considered as more accurate [67,68]. PI profiles indicate historical epochs during which a partition is most likely to provide phylogenetic signal but do not discount for the misleading effect of homoplasy caused by convergence to the same character state in divergent lineages [27,67,68]. Therefore, one must be careful when interpreting PI profiles. When reading PI profiles from tips to root, PI of the marker increases until reaching its PImax. Once the profile has crested, there are more and more sites that are evolving faster than the optimal rate, which can result in homoplasy (“phylogenetic noise”, [68,69]. Homoplasy plays a greater effect when the internodes are short. Therefore, when trying to resolve relationships, it is preferable to use character sets with PI profiles peaking deeper in time than the epoch of interest [68].

Bayesian and ML analyses of ClustalW and MAFFT alignments produced similar topologies (see results) and informativeness was calculated using the MAFFT datasets (6 partitions, without Gblocks cleaning, default-Gblocks cleaning, relaxed-Gblocks cleaning). Site rates as inferred by MrBayes (report siterates=yes command) were compiled into PhyDesign formatted files (see PhyDesign FAQ) and used as input rates. Those “vector files” are available upon request. The ultrametric tree was obtained from the MAFFT ML tree (6 partitions) using PATHd8 [70] by arbitrarily setting the root to 1.

## Results

### 1. Impact of alignment strategy

The final matrix contained 81 sycoecine specimens representing 56 ingroup species and six outgroups. Outgroup ITS2 sequences and *Bruchophagus caucasicus, Haltichella rufipes* and *Megastigmus aculeatus RpL27a* sequences were too divergent to be reliably aligned and were consequently excluded from the analyses. No stop codons or frame shifts were detected in the protein coding regions. 

Numbers of aligned base pairs, variable sites and parsimony-informative sites for each gene region used in this study are summarized in Table 3. MAFFT and ClustalW alignments of ITS2 and *RPL27a* resulted in datasets with similar properties (Δ total sites _MAFFT/ClustalW_ = -2 for ITS2, 28 for *RPL27a*; Δ parsimony informative sites _MAFFT/ClustalW_ = -8 for ITS2, -4 for *RPL27a*). Removing highly divergent alignment blocks using Gblocks with default parameters dramatically reduced the number of parsimony-informative sites (about 95% loss for ITS2 and between 66% (ClustalW) and 70% (MAFFT) loss for *RPL27a*). This loss resulted in far less resolved ITS2 and *RPL27a* trees, when either the MAFFT + Gblocks default or ClustalW + Gblocks default datasets were analysed (Figures S17, S20 and see Table S3 for a comparison of tree resolutions). The Gblocks-relaxed parameters cleaning only slightly affected the resolution of the ITS2 tree and did not affect the resolution of the *RPL27a* tree (Table S3, Figures S18, S21). 

**Table 3:** Numbers and percentages of aligned base pairs, variable sites and parsimony-informative sites for the gene regions used in this study.

**Partition**	**Total sites**	**Variable sites**	**Parsimony-informative sites**
mtDNA	2168	1082 (49.9%)	821 (37.9%)
*EF-1α*	516	156 (30.2%)	115 (22.3%)
*mago nashi*	309	108 (35.0%)	87 (28.2%)
ITS2 Alignment ClustalW	705	461 (65.4%)	345 (48.9%)
ITS2 Alignment ClustalW + Gblocks default	88	23 (26.1%)	17 (19.3%)
ITS2 Alignment ClustalW + Gblocks relaxed	338	187 (55.3%)	155 (45.9%)
ITS2 Alignment MAFFT	703	420 (59.7%)	337 (47.9%)
ITS2 Alignment MAFFT + Gblocks default	87	23 (26.4%)	17 (19.5%)
ITS2 Alignment MAFFT + Gblocks relaxed	373	229 (61.4%)	199 (53.3%)
*RpL27a* Alignment ClustalW	658	380 (57.8%)	258 (39.2%)
*RpL27a* Alignment ClustalW + Gblocks default	275	117 (42.5%)	86 (31.3%)
*RpL27a* Alignment ClustalW + Gblocks relaxed	543	321 (59.1%)	251 (46.2%)
*RpL27a* Alignment MAFFT	686	358 (52.2%)	254 (37.0%)
*RpL27a* Alignment MAFFT + Gblocks default	261	105 (40.2%)	75 (28.7%)
*RpL27a* Alignment MAFFT + Gblocks relaxed	542	338 (62.4%)	247 (45.6%)

For all partitions the best-fitting model chosen by MrAIC was GTR+I+Γ. We used a discrete gamma approximation [71] with four categories. A total of four analyses were performed per six combined datasets (2 partitioning schemes (5 versus 6 partitions) each analysed using ML and Bayesian methods, Table 4). For all bayesian analyses, after discarding 25% of the samples as burnin, ESS value of each parameter largely exceeded 200, which showed that convergence was reached. Twenty-four combined trees were obtained (Figures S1-S12) and deposited on TreeBase (http://purl.org/phylo/treebase/phylows/study/TB2:S14838). For all datasets, Bayes factor indicated that the most complex partitioning scheme (6 partitions) was preferred over the least complex one (5 partitions) (Table 4). 

**Table 4:** Arithmetic and harmonic means (lnL) for trees obtained in Bayesian analyses based on alternative alignment and partitioning strategies.

**Dataset**	**Partitioning strategies**	**Alignment**	**Figures**	**Harmonic Mean (LnL)**	Bayes factor (BF**)
1	P1 : mtDNA, *EF-1α*, ITS2, *RpL27a*, *mago nashi* [5 partitions, 54 free parameters*]	ClustalW	2, 4, S1	-41666.23	BF_2/1_ = 2487.0
2	P2 : mtDNAcodon1&2, mtDNAcodon3, *EF-1α*, ITS2, *RpL27a*, *mago nashi* [6 partitions, 65 free parameters*]	ClustalW	4, S2	-40397.38	
3	P1	ClustalW + Gblocks default	4, S3	-31414.10	BF_4/3_ = 2432.8
4	P2	ClustalW + Gblocks default	4, S4	-30172.36	
5	P1	ClustalW + Gblocks relaxed	4, S5	-37383.72	BF_6/5_ = 2459.5
6	P2	ClustalW + Gblocks relaxed	4, S6	-36128.63	
7	P1	MAFFT	1, 4, S7	-41369.61	BF_8/7_ = 2474.5
8	P2	MAFFT	4, S8	-40107.02	
9	P1	MAFFT + Gblocks default	4, S9	-31258.75	BF_10/9_ = 2432.1
10	P2	MAFFT + Gblocks default	4, S10	-30017.36	
11	P1	MAFFT + Gblocks relaxed	4, S11	-38341.39	BF_12/11_ = 2457.9
12	P2	MAFFT + Gblocks relaxed	4, S12	-37087.12	

* excluding branch length and topology parameters. Given that all parameters are unlinked among partitions, the number of free parameters of the composite model is the sum of the free parameters of its submodels. For all partitions the best-fitting model chosen by MrAIC was GTR+I+Γ (10 free parameters).

** BF_1/0_ = 2*(ln L1 – ln L0) + (P1-P0) * ln (0.01) where ln Li and Pi are respectively, the harmonic mean of the ln likelihoods and the number of free parameters of model i. BF values from 2 to 6 were considered positive evidence, from 6 to 10 as strong evidence, and > 10 as very strong evidence favouring the alternative hypothesis over the null hypothesis.

**Figure 3:**
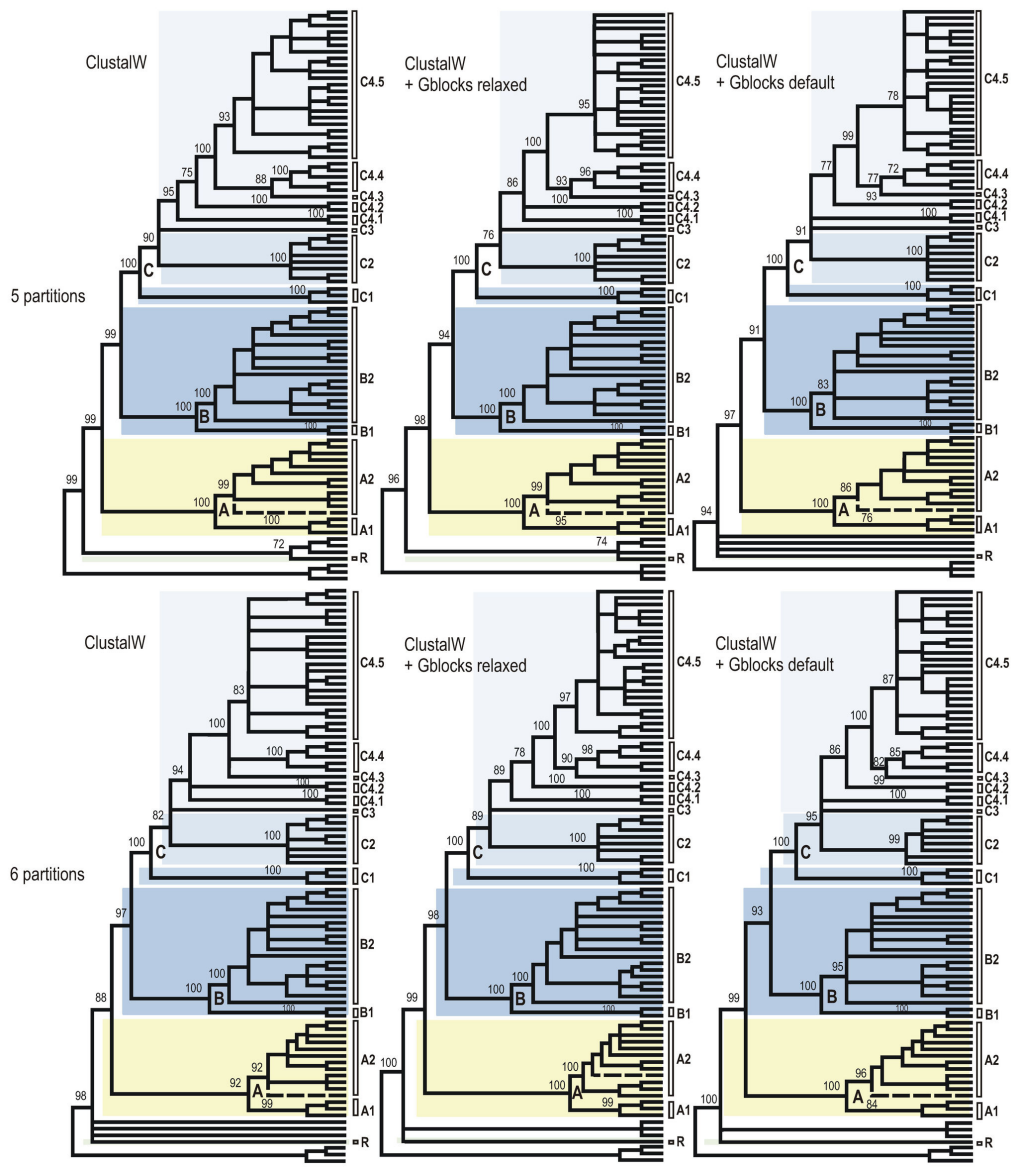
Cladograms of relationships among the Sycoecinae obtained from the ClustalW alignment of the combined dataset under three different alignment strategies and two partitioning schemes. The corresponding ML and Bayesian trees are given in Figures 2, S1-S6. Nodes with BP support < 70% have been collapsed and BP supports for main clades are indicated at nodes. Uppercase letters refer to clades discussed in the text (see also Figure 2). The dashed line indicates the only taxon with a conflicting position among trees (see text).

**Figure 4:**
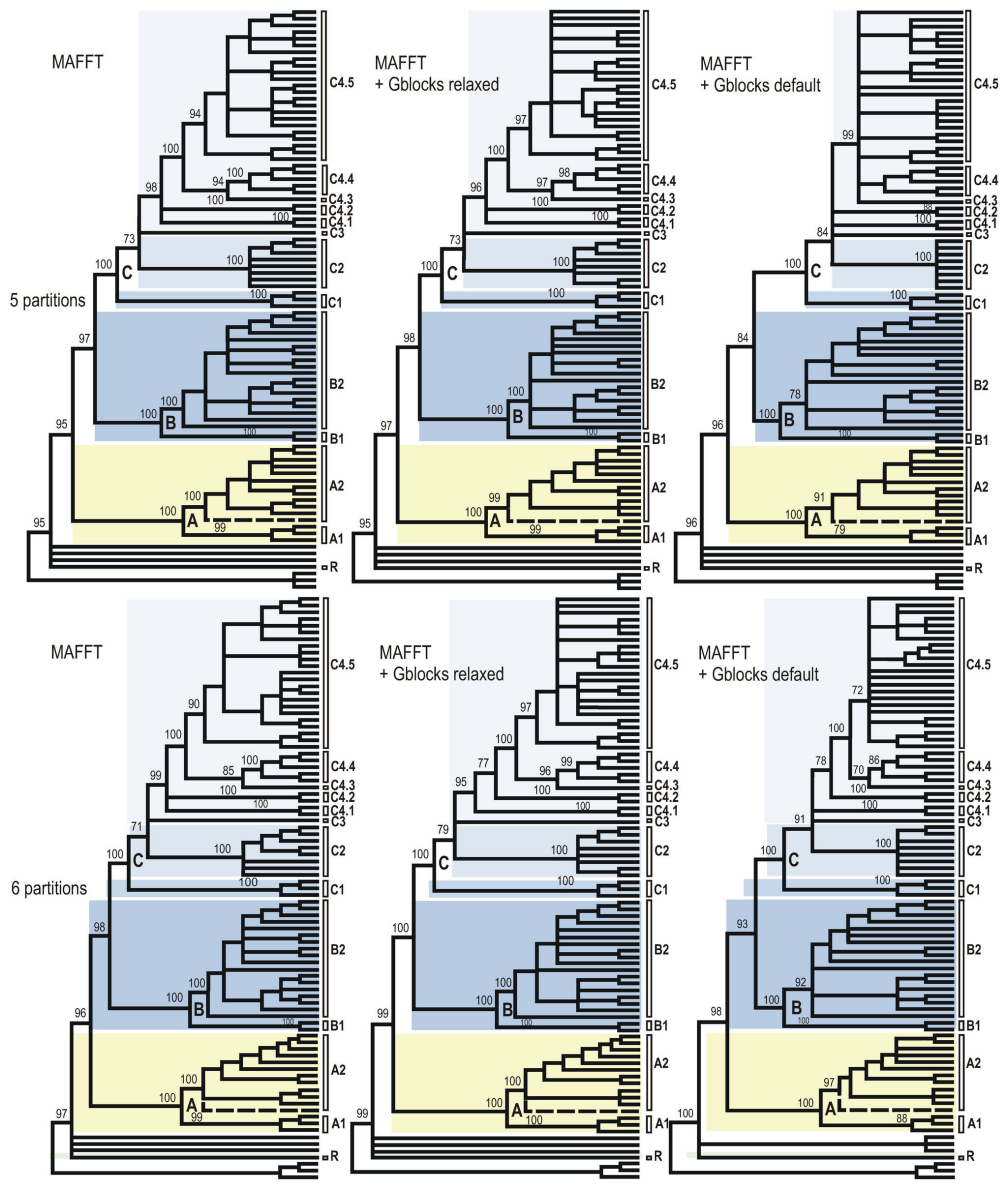
Cladograms of relationships among the Sycoecinae obtained from the MAFFT alignment of the combined dataset under three different alignment strategies and two partitioning schemes. The corresponding ML and Bayesian trees are given in Figures 1, S7-S12. Nodes with BP support < 70% have been collapsed and BP supports for main clades are indicated at nodes. Uppercase letters refer to clades discussed in the text (see also Figure 1). The dashed line indicates the only taxon with a conflicting position among trees (see text).

All trees had well-resolved backbones and three major clades (A, B, C) were identified (Figures 1-4, S1-S12). As expected the Gblocks-default topologies (Figures 3-4; S3-S4; S9-S10) showed poorer nodal supports at shallower (within C2, C4.5 clades) and intermediate nodes (within clade C). The MAFFT - 6 partitions datasets was used as input for SH and AU tests of alternative topologies (Table S2). Those tests indicated that the Gblocks-default parameters topologies were all significantly different from the others, though our visual comparison of trees revealed that conflicting nodes had poor supports. The only topological conflict between all 24 topologies with a BP > 70 was the position of *Diaziella* sp. nov. 12 ex *Ficus* sp. (n° 2989_01) (Figures 3-4, dashed line). Its position was either unresolved (5 trees), as sister to the rest of clade A2 (6 trees, 80 < BP < 92; 0.52 < PP < 1.00) or nested within clade A2 (ClustalW+Gblocks relaxed-6 partitions tree, BP=75, PP=0.89). This uncertainty was probably due to the amount of missing data for this taxon (only 3 markers on 6, Table S1). 

### 2. Evolutionary properties and phylogenetic informativeness (PI) of markers

Model parameter estimates for each partition of the Bayesian analyses of the MAFFT combined dataset - 6 partitions (mean and 95% credibility intervals) are reported in Table 5. Parameter estimates from the analysis of the Clustal W combined dataset (6 partitions) were similar (available upon request). As might be expected, the mtDNA partition showed extreme base compositional bias (69.7% and 96.0% of A/T for mtDNA first + second codon positions and third codon positions respectively). Among the nuclear genes analysed, while *RPL27a* and *mago nashi* were A/T-biased (73.6% and 65.4 % respectively), *EF-1α* and ITS2 show more or less even base composition (50.9% and 55.2% of A/T, respectively). There was a higher rate of A-G and C-T transitions for all partitions (52.6% for ITS2, 64.1% for mtDNA first + second codon positions, 68.8% for *RPL27a*, 80,3% for *EF-1α*, 84.9% for *mago nashi* and 88.8% for mtDNA third codon positions). The mtDNA third codon positions showed a high rate of C-T transitions relative to any other transformations (57.0%). For the nuclear genes, the rates of transitions were close and so were the rates of transversions. The ITS2 rate matrix was the least skewed towards one type of change over another. The gamma shape parameter α was higher for the nuclear genes than for the mtDNA partitions, indicating that nuclear genes show less rate heterogeneity among sites than mitochondrial genes (Table 5). The rate multiplier parameters (m) indicated that rates of substitution were different among partitions. The mtDNA third codon positions evolved about twenty-three times faster than the fastest nuclear gene (ITS2). *EF-1α* was the most slowly evolving marker. 

**Table 5:** Evolutionary properties of the partitions used in this study.

**Partitions**	**r A-C**	**r A-G**	**r A-T**	**r C-G**	**r C-T**	**r G-T**
*EF-1α*	0,069 (0,042-0,096)	0,317 (0,238-0,397)	0,044 (0,018-0,073)	0,04 (0,02-0,062)	0,486 (0,402-0,564)	0,044 (0,02-0,07)
ITS2	0,118 (0,093-0,143)	0,251 (0,216-0,287)	0,159 (0,133-0,185)	0,091 (0,068-0,114)	0,275 (0,239-0,312)	0,106 (0,083-0,128)
*mago nashi*	0,053 (0,03-0,08)	0,389 (0,302-0,477)	0,045 (0,026-0,066)	0,032 (0,008-0,06)	0,46 (0,372-0,552)	0,022 (0,002-0,042)
*RpL27a*	0,075 (0,054-0,097)	0,327 (0,28-0,375)	0,068 (0,054-0,082)	0,081 (0,046-0,116)	0,361 (0,314-0,411)	0,088 (0,066-0,112)
mtDNA 1&2 codon positions	0,065 (0,044-0,086)	0,301 (0,252-0,352)	0,117 (0,095-0,14)	0,132 (0,093-0,173)	0,34 (0,289-0,394)	0,046 (0,03-0,06)
mtDNA 3 codon positions	0,009 (0-0,02)	0,318 (0,229-0,408)	0,003 (0,002-0,004)	0,089 (0-0,219)	0,57 (0,444-0,682)	0,011 (0-0,022)
**Partitions**	**pi A**	**pi C**	**pi G**	**pi T**	**alpha**	**rate multiplier (m)**
*EF-1α*	0,275 (0,238-0,312)	0,25 (0,217-0,283)	0,241 (0,207-0,275)	0,234 (0,203-0,268)	2,749 (0,141-4,323)	0,037 (0,03-0,044)
ITS2	0,255 (0,232-0,28)	0,213 (0,191-0,235)	0,236 (0,212-0,26)	0,297 (0,272-0,321)	3,318 (1,981-4,874)	0,239 (0,205-0,271)
*mago nashi*	0,359 (0,313-0,404)	0,167 (0,135-0,199)	0,179 (0,146-0,216)	0,295 (0,253-0,337)	7,623 (0,29-18,925)	0,062 (0,048-0,075)
*RpL27a*	0,352 (0,324-0,382)	0,129 (0,111-0,147)	0,136 (0,116-0,156)	0,384 (0,355-0,413)	4,389 (1,761-7,465)	0,12 (0,105-0,139)
mtDNA 1&2 codon positions	0,293 (0,272-0,314)	0,145 (0,127-0,164)	0,158 (0,14-0,175)	0,404 (0,38-0,427)	0,606 (0,427-0,81)	0,08 (0,066-0,091)
mtDNA 3 codon positions	0,5 (0,482-0,516)	0,024 (0,022-0,026)	0,017 (0,015-0,019)	0,46 (0,443-0,477)	0,347 (0,319-0,377)	5,503 (5,44-5,561)

Mean and 95% credibility intervals of the model parameters for each partition included in the Bayesian analyses of the MAFFT combined datasets (6 partitions) are reported. Parameter estimates from the analysis of the Clustal W combined dataset (6 partitions) were similar (available upon request).

**Figure 5:**
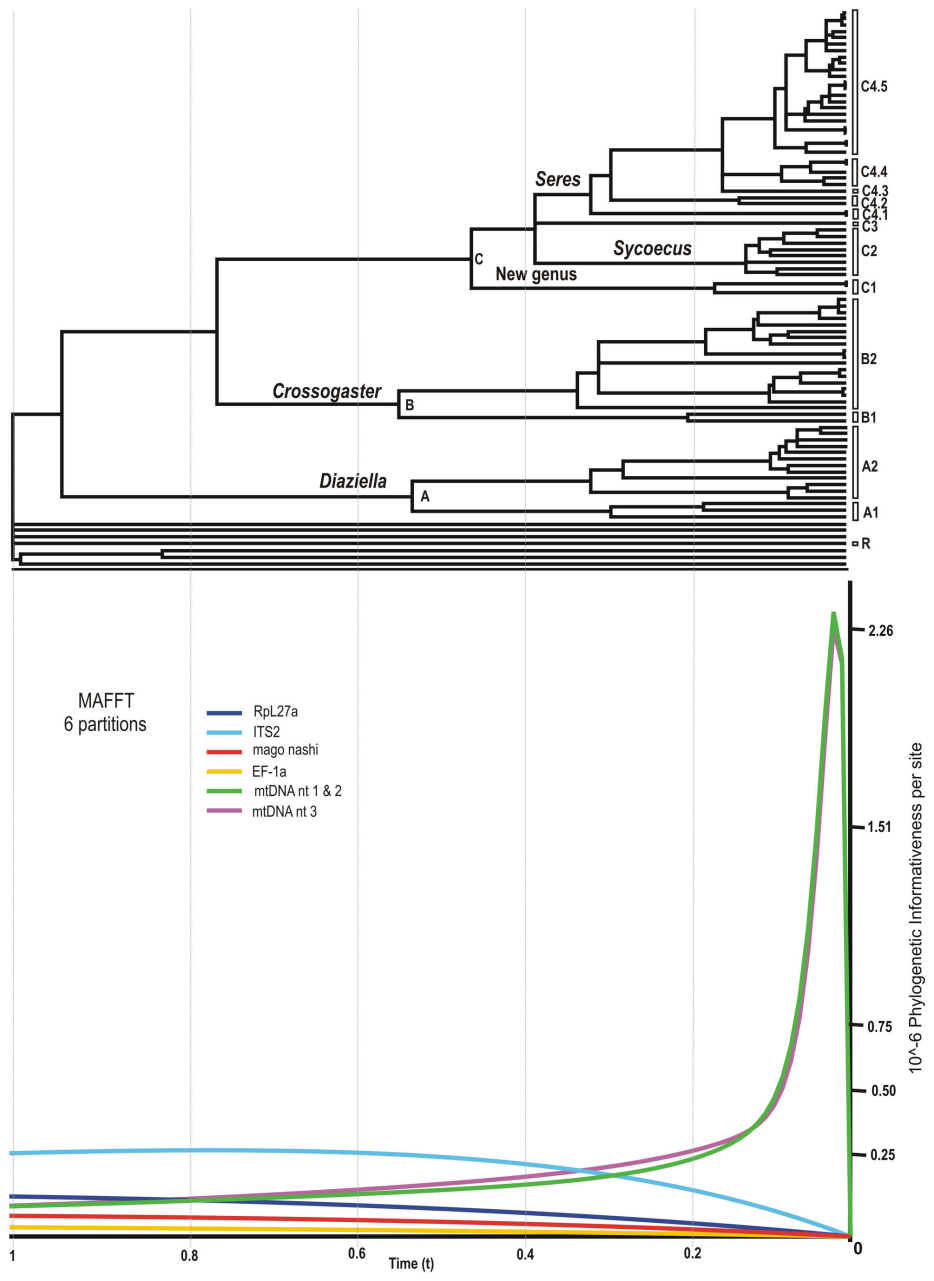
Per site phylogenetic informativeness profiles of the markers based on the MAFFT dataset (6 partitions). Uppercase letters refer to clades discussed in the text (see also Figures 1 & 2).

For the complete dataset (MAFFT without Gblocks cleaning - 6 partitions), mtDNA (first+second and third codon positions) showed sharp peaks of informativeness at shallower nodes (Figure 5). Then, the profiles declined showing potential for homoplasy, which is not surprising when considering mtDNA evolutionary properties (Table 5). This prediction is confirmed by the observation of the real performance of the mtDNA marker (i.e. the single-gene tree) in resolving shallower relationships (Figure S13). Interestingly, PI profile suggested that ITS2 was informative across the whole phylogeny. Again, this prediction was confirmed by the observation of the ITS2 tree (Figure S16), on which main clades were recovered. Overall, shapes of the PI profiles correlated well with the corresponding single-gene tree topologies (Figures S13-S16, S19) and, more precisely, with the distribution of branch lengths across the topologies. The mtDNA topology had longer terminal than internal branches. The deeper in the tree, the shorter the branches, though enough information remained to resolve, at least partially, deeper relationships. Terminal branches of the ITS2 tree were much shorter than those of the mtDNA tree, but longer than those of the *RPL27a* tree. The deeper branches of the ITS2 tree were longer than those of the *RPL27a* tree. Neither *EF-1α* nor *mago nashi*, the slowest evolving markers contained enough information to resolve relationships within species complexes and sometimes even between species (Figures S14, S15). Our qualitative comparison of single gene tree topologies would lead us to the following ranking in informativeness at shallower nodes : mtDNA > ITS2 > *RPL27a* > *mago nashi* ^~^ *EF-1α*, which corresponded to the relative position of the PI profile curves. At deeper nodes, this comparison would lead us to rank the markers as follows: ITS2 (longest branches) > *RPL27a *> mtDNA ^*~*^ *mago nashi* ^~^ *EF-1α*, which is again compatible with the PI profiles on Figure 5. *mago nashi* PI profile curve lays a bit above *EF-1α* PI profile curve probably because some longer branches do appear in the *mago nashi* topology (Figure S15).

PI profiles were similar, when a relaxed Gblocks cleaning was performed (Figure S22), and, as expected, the informativeness of ITS2 and *RPL27a* sharply decreased, when default parameters were used to clean the alignment (Figure S23). A bit surprisingly, the default-Gblocks mtDNA PI score was the highest across the whole phylogeny, though RPL27a showed an equivalent resolution at intermediate nodes (Figures S13, S20). 

### 3. Sycoecinae relationships

As we were interested in relationships at both lower and higher levels, we focussed on trees from the ML and Bayesian analyses of the complete datasets (ie without Gblocks cleaning). We deliberately kept what could be considered the “worst” tree (ie ClustalW + 5 partitions, Figure 2, S1) and the “best” tree (ie MAFFT + 6 partitions, Figures 1, S8) as no one was preferred by the SH/AU tests of alternative topologies. We also wanted to over-emphasize that the few topological differences only occurred between nodes that were either poorly supported in both trees or poorly supported in at least one of the two trees. In the following section, BP and PP values are mentioned as followed: BP ClustalW-tree/BP MAFFT-tree; PP ClustalW-tree/PP MAFFT-tree or are summarized by unique values when no differences between the trees were observed.

The genus *Robertsia* never clustered with other Sycoecinae species. However, a monophyletic Sycoecinae could not be rejected by both SH and AU tests (Table S2). 

All the remaining sycoecine genera formed a strongly supported clade (BP = 99/96, PP = 1.00). The phylogenies supported the monophyly of the genera *Crossogaster* (BP = 100, PP = 1.00), *Diazella* (BP = 100, PP = 1.00) and *Sycoecus* (BP = 100, PP = 1.00) but recovered neither *Philocaenus* nor *Seres* as monophyletic. Both AU and SH tests rejected a monophyletic *Seres* (Table S2).

The Sycoecinae (genus *Robertsia* excepted) clustered into three major clades: *Diaziella* species (clade A) appeared sister to a clade grouping all Afrotropical Sycoecinae (BP = 99/98, PP = 1.00/1.00), which clustered into two clades: clade B (*Crossogaster* spp.) and clade C (*Philocaenus*, *Sycoecus* and *Seres* spp., BP = 100, PP = 1.00). 

Within clade A, *Diaziella yangi*, *D. bizarrea* and *D*. sp. ex *F. lawesii* (clade A1, BP = 100/99, PP = 1.00), were sister to a clade (clade A2, BP = 99/100, PP = 1.00) that grouped all other *Diaziella* species. 

Within clade B, *Crossogaster* sp. nov. 5 + *C. michaloudi* (clade B1; BP = 100, PP = 1.00) were sister to the remaining *Crossogaster* species (clade B2; BP = 100, PP = 1.00). Interestingly, *Crossogaster* sp. nov. 6 ex *F. louisii* grouped with the *C. odorans* clade (BP = 99/100, PP = 1.00). 

Within clade C, *Seres* species hosted by *Ficus polita* and *F. bubu* (clade C1, BP = 100, PP = 1.00) were recovered sister to a strongly supported clade (BP = 90/71, PP = 0.99/0.97) including 1) *Sycoecus* species (clade C2, BP = 100, PP = 1.00), 2) an undescribed *Philocaenus* species (“clade” C3) and, 3) a strongly supported clade (C4, BP = 95/99, PP = 1.00) clustering *Philocaenus* species and all the remaining *Seres* species (type species of the genus *Seres* included). Within clade C4, four groups of *Philocaenus* species could be distinguished (C4.1 to C4.4). *P. barbarus* could be a complex of at least three species. Furthermore, *P. liodontus* and *P. rotundus* did not form mutually exclusive clades. 

Finally, our analyses recovered several cases, where most closely related species were sampled from the same host tree (black boxes in Figures 1 & 2), which could indicate sympatric speciation (Genera *Diaziella*, *Crossogaster*, *Sycoecus* and *Philocaenus*) .

## Discussion

### 1. Impact of alignment and partitioning strategies, accuracy of phylogenetic informativeness profiles

As underlined by Rokas et al. [26], one should be cautious when making generalizations about the taxonomic level at which a given marker might be useful. Genera belonging to different insect families may span a range of evolutionary ages, and hence evolutionary properties and phylogenetic informativeness (PI) of markers should be used as guidelines only [69]. Despite the potential problems while applying these properties across different families, we believe that our results may be useful for the designing of future phylogenetic studies between and within chalcidoid families, especially within the pteromalid complex [9]. It as been shown that MAFFT outperformed ClustalW on several datasets (e.g. [49]), however, this was not the case for our dataset. We also highlighted that complex partitioning schemes, even favoured by Bayes Factors did not lead to different topologies. Then, between what could be considered the “worst” tree (ie ClustalW + 5 partitions, Figures 2, S1) and the “best” tree (ie MAFFT + 6 partitions, Figures 1, S8) only few topological differences were identified and all of them occurred between nodes that were either poorly supported in both trees or poorly supported in at least one of the two trees. We also showed that using Gblocks with default settings on our alignments dramatically decreased the number of parsimony informative sites (Table 3) and tree resolution. This indicates that one must be cautious when using Gblocks to clean alignments. Gene properties should be studied before Gblocks refinement and selection of blocks should be used only when focussing on relationships at high level, especially when an important number of markers are concatenated [72].

Overall, shapes of the phylogenetic informativeness (PI) profiles (Figure 5) correlated well with the distribution of branch lengths on the corresponding single-gene tree topologies (Figures S13-S21). It is noteworthy that ITS2 contained information spanning the whole tree and mtDNA did contain signal to contribute resolving shallower but also deeper nodes of the phylogeny. It was surprising that the mtDNA PI score was still the highest of any gene at the root of our phylogeny when we profiled the informativeness of each partition of the MAFFT-Gblocks default dataset (Figure S23). Klopstein et al. [67] suggested that PI could be biased towards fast rates (due to the whole set-up of the measure). This could explain the relative position of the curves on the MAFFT-Gblocks default PI profiles. Indeed, for this dataset, mean values of m (rate multiplier) scores for the two partitions: mtDNA-third codon (mtDNAnt3) and mtDNA-first + second codon (mtDNAnt1&2) positions were the highest of any partitions (Table 6). Nevertheless, the mtDNA PI score for the MAFFT dataset was not the highest of any gene at the root of our phylogeny (Figure 5), though the rate multiplier of the mtDNAnt3 partition was the highest of any other partitions (Table 5). Moreover, the mean values of m scores for the ITS2 and *RPL27a* partitions were higher than the mean value of m score of the mtDNAnt1&2 partition, though the mtDNAnt1&2 PI score was higher than ITS2 and *RPL27a* PI scores at shallower and intermediate nodes (Figure 5).

**Table 6:** Rate multiplier values (m) for each partitions included in the Bayesian analyses of the MAFFT + Gblocks default parameters and MAFFT + Gblocks relaxed parameters combined datasets (6 partitions).

**Partitions**	**rate multiplier (m)**	**rate multiplier (m)**
	**MAFFT + Gblocks (default parameters)**	**MAFFT + Gblocks (relaxed parameters)**
*EF-1α*	0.041 (0.033-0.044)	0.037 (0.029-0.044)
ITS2	0.065 (0.032-0.103)	0.161 (0.137-0.186)
*mago nashi*	0.069 (0.053-0.086)	0.063 (0.050-0.077)
*RpL27a*	0.065 (0.052-0.081)	0.116 (0.100-0.134)
mtDNA 1&2 codon positions	0.083 (0.070-0.096)	0.079 (0.067-0.092)
mtDNA 3 codon positions	4.364 (4.328-4.397)	5.022 (4.976-5.068)

### 2. Monophyly of the sycoecine genera and new classification

#### 2.1. Robertsia (clade A)

The genus *Robertsia* is morphologically very distinct from the other sycoecine genera (Table 1) and is the only Sycoecinae genus with apterous males [6,24]. Our study shows that taxonomic affinities of *Robertsia* are ambiguous. However, no definitive conclusion can be drawn from our results. Further studies, including more representatives of the genus and New Guinean pteromalid genera are required. These studies could for example discard a possible long-branch attraction artifact between *Robertsia* and the outgroup species used in the present study or a negative impact of missing data (only ITS2, *RPL27a* and *mago nashi* could be obtained for this taxa). 

#### 2.2. Diaziella (clade A)

*Diaziella* is strongly supported as monophyletic across all analyses. This genus is also well defined morphologically [22]. All *Diaziella* species are associated with *Ficus* species occurring in Oriental tropical forests and belonging to the subsection *Conosycea* (Table 1, Figures 1 & 2). Our analyses highlighted two species-groups within the genus (A1 and A2). The species associated with the basal most *Conosycea* fig trees *Ficus glaberrima*, *F. curtipes* and *F. lawesii* [44,73] clustered in a strongly supported clade (clade A1). These species are characterized by a metallic tinge on the female thorax. Within the clade A2, two small radiations of eight and three species are hosted respectively by *F. sumatrana* and *F. sundaica*. Females of these species are characterized by a non-metallic thorax (black or yellow).

#### 2.3. Crossogaster (clade B)

*Crossogaster* is recovered as monophyletic with strong support in all the analyses, a result also supported by morphology (Table 1). *Crossogaster* species are associated with four of the six *Galoglychia* subsections namely *Platyphyllae, Chlamydodorae, Crassicostae and Caulocarpae* that occur in afrotropical forests and savannas (Figures 1 & 2, Table 1).

*Crossogaster* splits into two well supported clades (B1 and B2). Based on morphological interpretation, van Noort (1994a) split *Crossogaster* into two monophyletic species-groups and excluded three unassigned basal species. Both the *C. odorans* species-group (characterised by the presence of elongate multiporous plate sensilla (MPS) on five antennal funicle segments), and the *C. triformis* species-group (characterised by the presence of placoid MPS on the usually four antennal funicle segments) are included in our clade B2, but each group is not supported suggesting that MPS appearance and number of funicular segments is homoplasious. Our clade B1 corresponds with two of the species exhibiting plesiomorphic characters, *C. michaloudi* and *C. lachaisei* (*Crossogaster* sp. nov 5 is closely related to *C. lachaisei*). The third species exhibiting plesiomorphic characters, *C. inusitata*, is a basal representative included in clade B2. However, more representative taxa need to be included to redefine the *Crossogaster* species-groups.

Interestingly, *Crossogaster odorans* appeared to cluster into several subclades, which is not surprising since *Crossogaster odorans* was described from specimens associated with *F. burkei*. Van Noort (1994a) acknowledged that *C. odorans* probably comprised a species complex and discussed the associated morphological variation.

#### 2.4. A new genus basal to Seres + Sycoecus (clade C1)

Since this clade is strongly supported with a long-branch length and is characterized by strong morphological synapomorphies, it should represent a new genus that will be described elsewhere (van Noort & Rasplus, in prep). Species belonging to clade C1 exhibit a unique plate of teeth on the fore tibia of females and a metallic green head in males. *Seres wardi* was first identified as a distinct member of *Seres* based on morphology of both sexes [15]. Together with another distinct member (*Seres longicalcar*), *S. wardi* was placed in the genus *Seres* based on the perceived synapomorphic positioning of the propodeal spiracles [15], which now appears to have been derived independently on several occasions rendering *Seres* as currently recognised to be a polyphyletic assemblage. Species belonging to this clade are only associated with fig trees of the section *Galoglychia* subsection *Caulocarpae*, all of them occurring in afrotropical rainforests (Figures 1 & 2). 

#### 2.5. Sycoecus (clade C2)

*Sycoecus* is recovered as a strongly supported monophyletic clade across all the analyses, confirming the morphological delimitation of this genus that exhibits striking apomorphies (Table 1). All species of *Sycoecus* are associated with fig trees from the subsection *Cyathistipulae* that grow in the evergreen forests of Central, West and East Africa (Figures 1 & 2, Table 1).

#### 2.6. A new genus associated with subsection Crassicostae ? (lineage C3)

Unfortunately, we were able to include only the one known *Philocaneus* species associated with the subsection *Crassicostae*, a subsection with a species distribution centred in the poorly sampled Congo basin. The remaining seven species in this subsection have not yet had their associated fig wasp faunal assemblages sampled or if sampled (*F. louisii* and *F. elasticoides*) potential *Philocaenus* species were not reared. Our results show that the phylogenetic position of *Philocaenus* sp. nov. ex. *F. usambarensis* is ambiguous (Figures 1 & 2, C3). Although it is recovered as sister to all other *Philocaenus* species, this relationship is not supported. Morphologically this species is also exceptional. The mandibles are very different and the ovipositor is exceptionally long for *Philocaenus* being longer than half of the metasomal length. There are a total of eight *Ficus* species in subsection *Crassicostae*. Given the host relationships of *Philocaenus*, this subsection may potentially host further related *Philocaenus* species. If inclusion of these species in the phylogeny results in a monophyletic clade, erection of a new genus may be warranted. However, for the time being, we prefer to keep this species as part of the genus *Seres* (in its extended delimitation, see here under).

#### 2.7. *Seres*, *sensu nov*. (Clade C4)

In all analyses, a clade clustering some *Seres* species and including *S. armipes*, type species of the genus *Seres* (clade C4.4) makes *Philocaenus* paraphyletic. In our opinion *Philocaenus* should be synonymized under *Seres*. Consequently, the following new combinations are proposed: *Seres arrujumensis* (van Noort, 2006) comb. nov., *S.* bakeri (van Noort, 1994) comb. nov., *S. barbarus* (Grandi, 1955) comb. nov., *S.* barbatus (Grandi, 1952) comb. nov., *S. bifurcus* (van Noort, 1994) comb. nov., *S.* boučeki (Wiebes, 1982) comb. nov., *S. cavus* (van Noort, 1994) comb. nov., *S.* clairae (van Noort, 1994) comb. nov., *S. comorensis* (van Noort, 1994) comb. nov., *S.* comptoni (van Noort, 1994) comb. nov., *S. geminus* (van Noort, 1994) comb. nov., *S.* hippopotomus (van Noort, 1994) comb. nov., *S. insolitus* (van Noort, 1994) comb. nov., *S.* jinjaensis (van Noort, 1994) comb. nov., *S. levis* Waterston, 1920 (Original combination restored). *S. liodontus* (Wiebes, 1979) comb. nov., *S.* medius (van Noort, 1994) comb. nov. , *S. quatuordentatus* (van Noort, 1994) comb. nov. , *S.* rasplusi (van Noort, 1994) comb. nov., *S. rotundus* (van Noort, 1994) comb. nov., *S.* silvestrii (Grandi, 1916) comb. nov., *S. ugandensis* (van Noort, 1994) comb. nov., *S.* warei (van Noort, 1994) comb. nov., *S. zambesiacus* (van Noort, 1994) comb. nov. 

We give a diagnosis of *Seres* in its new delimitation, and a complete list of *Seres* species in Appendix S1. In summary, the genus is characterized morphologically by 1) an eighth urotergite spiracle not enlarged 2) the presence of two labial and three maxillary palp segments, 3) ventral tentorial pits in close apposition, 4) forewing marginal vein thickened 5) the outer tooth of male mandibles longer than the inner mandible.

Molecularly, the genus *Seres* in its new sense comprises five distinct species groups (C4.1-C4.5) that largely but not always exactly correspond to the species groups previously defined on morphological characters. A detailed study about *Seres* species groups is in preparation and will be published elsewhere. 

### 3. Evolutionary implications of the relationships among the Sycoecinae

As mentioned above, all trees have well-resolved backbones, which indicates stability of higher-level relationships to different alignment strategies (Figures 1-4, S1-S12). Based on this robust phylogenetic hypothesis of relationships among the Sycoecinae, we give preliminary results regarding the evolutionary history of the group.

####  3.1. High degree of morphological convergence

The genera *Crossogaster* and *Seres* (*sensu nov*.) are morphologically similar (head and body shapes) and in a morphological phylogenetic assessment were recovered as sister taxa due to perceived synapomorphies [74] that we here show are likely to represent homoplasious convergence. Many of the species in both genera have a single comb of backward pointing teeth on the mandible, and a single comb of teeth on the fore tibia. *Crossogaster* and *Seres* species are often found on the same host species, particularly within the subsections *Platyphyllae* and *Chlamydodorae*. Contrary to the conclusions based on the morphological analysis of the Sycoecinae, our molecular phylogenies show that *Seres* and *Crossogaster* are not each other’s closest relative. Therefore, the morphological similarity of the two lineages is probably the product of convergent evolution. Indeed, both lineages have probably evolved similar adaptations under identical selection pressures due to the constraints of internal oviposition in the same host. This has already been demonstrated for the sycoecines and their associated pollinating fig wasps, for which head shape (calculated as the ratio of head width to head length) and fresh fig diameter of host fig trees were correlated [14]. As mentioned above, morphological convergences are also common between the species of both genera confounding the ability to tease apart phylogenetic relationships based on morphology alone.

#### 3.2. Several cases of probable speciation on a single host fig species

Interestingly, our analyses show that speciation on a single host *Ficus* species has probably occurred recurrently in different sycoecine genera. *Ficus sumatrana* and *F. sundaica* host eight and three related *Diaziella* species respectively (Figures 1 & 2). This is the first time so many congeneric species of non-pollinating fig wasps (8) have been collected on the same tree and recorded from the same host fig species. All these species were morphologically clearly differentiated based on the head shape, antennae structure, wing patterns and mandible shape (pers. obs. SvN & JYR). It is noteworthy that all these species were collected from fig syconia that contained very few pollinators. *Ficus sumatrana* is passively pollinated and *Diaziella* species enter the fig covered with pollen. Given the successful reproduction in fig syconia with low pollinator incidence, it suggests that at least some of the *Diaziella* species may play a role in the pollination of their host, as previously reported about other sycoecine species [13].

Although less spectacular than the small radiation of *Diaziella* species on *F. sumatrana*, our results highlight several cases of apparent speciation on a single host fig species in the genera *Crossogaster* (twice), *Sycoecus* (once) and *Seres* (once) (Figures 1 & 2. Black boxes). Again, in all cases, the species were clearly differentiable based on morphology (pers. obs. SvN & JYR). It is noteworthy that outside our dataset, all known *Robertsia* species (4) have only ever been reared from *Ficus xylosycia* (*Malvanthera*) [24].

As for pollinators [1,5], sycoecine generation time is by far shorter than fig generation time, which could explain why sycoecine speciation by duplication is so common. A possible scenario would involve divergence between sycoecine species isolated on different populations of their host fig tree (e.g. by forest fragmentation). When secondary contact occurs, while fig trees can still interbreed, reproductive incompatibilities have evolved between sycoecine species precluding mating of species. As a consequence, sycoecine species co-occur on the same host tree [5]. Another scenario would be that co-occuring sycoecine species result from ecological speciation [75]. For example, the two species collected in the same *F. chirindensis* figs in Kenya (2968_01 and 2968_02) are clearly differentiable based on morphology (antennae structure, mandible shape) and coloration: one species is pale (2968_01, sp nov. 2) and the other is dark (2968_02, sp. nov. 3). As pale wasp species are attracted to light at night (see 76–79 for the Agaonidae and [21] for the Sycoecinae), we could hypothesize that the first species exploit pools of *F. chirindensis* figs attractive at night whereas the dark species exploit pools of figs attractive at day. It has been shown that co-occuring agaonid species may have different longevity and ability to resist desiccation [80]. This could be also hypothesized for sycoecines. Wasps with longer life span being more likely to disperse over longer distances [81]. Explaining the co-occurrence of eight *Diaziella* species on *F. sumatrana* is more puzzling, especially since the species do not exhibit morphological differences that could be linked to the exploitation of different niches (e.g. ovipositor length [82] or color). As mentioned above, one possibility would be that all the species have evolved in allopatry, in different forest fragments. Another explanation could be that *F. sumatrana* is a complex of sympatric species that share representatives of the different *Diaziella* species, all the species being sampled by chance on one *F. sumatrana* tree. Further studies are needed to understand which mechanisms have been involved.

#### 3.3. Comparison with available *Ficus host* phylogenies

Although current taxonomy of the Sycoecinae supports a degree of host specificity between the wasps and their *Ficus* hosts, our study reveals many examples of break-down in host-specificity. For instance, we frequently observe the association of more than one sycoecine species (often belonging to several genera) with one *Ficus* species (e.g. *Crossogaster* and *Seres* species Figs. 1 & 2). Furthermore, a single sycoecine species can be associated with more than one *Ficus* species (e.g. *S. medius*, *S.* liodontus). Such complex associations imply that events such as host-switches and speciation on host are common incidents in the evolutionary history of these independent lineages. 

**Figure 6:**
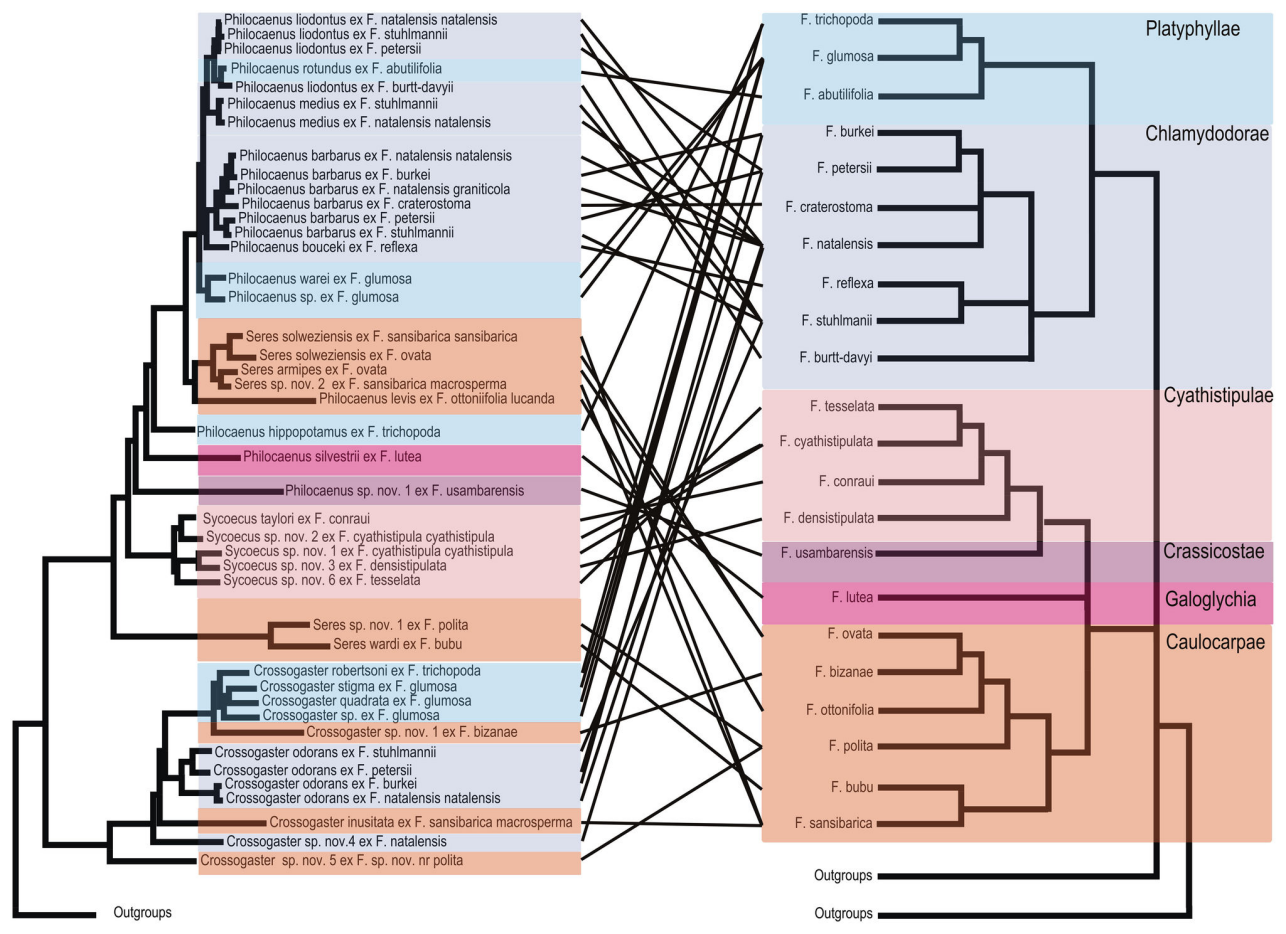
Compared phylogenies of the Afrotropical sycoecine fig wasps (this study) and their *Ficus* hosts (adapted from Rønsted et al. [94] and Renoult et al. [95]) .

Ostiolar morphology prevents entry into the fig cavity for wasps that are not specifically adapted [14,83,84]. Externaly ovipositing fig wasps do not have to conform to the morphological adaptations required to enter the fig cavity through a host-specific ostiole. Therefore, internally ovipositing wasps are thought to be highly host specific and less likely to experience host shifts than the externally ovipositing wasps [1,5,46,85]. However, these ideas are still contentious. Recent studies have shown that some externally ovipositing fig wasps may be highly host specific [5,86–88] and could have cospeciated with their host figs [86,87] but see 89,90. Overall, we lack extensive data on non-pollinating fig wasps host specificity and further cophylogenetic studies on representative samplings of both figs and wasps are required. Our results strongly suggest that strict cospeciation has not shaped the evolutionary history of both sycoecines and their host *Ficus* (Figure 6). It appears that the constraints of internal oviposition may not be enough to prevent host-switching events. However, further studies are needed to uncover the fine evolutionary history of the partners. These should especially focus attention on species that are associated with numerous fig hosts, the majority of which fall within *Ficus* subsection *Chlamydodorae*.

## Conclusion

Prior to this study, elucidation of the taxonomic relationships within the Sycoecinae through molecular phylogenetic analyses had not been attempted. The combined analysis of mitochondrial and nuclear gene regions resulted in a robust phylogenetic hypothesis of relationships among the Sycoecinae, validated by our morphological observations. This study contributes to the global effort to better understand how world fig wasp communities have been structured through space and time. With Agaonidae (*Ficus* pollinators, [44,91]), Sycophaginae [45,88] and Sycoryctinae [92], Sycoecinae is the fourth fig wasp group for which a worldwide phylogeny is now available. 

## Supporting Information

Table S1 **GenBank accession numbers.**
(PDF)

Table S2 **Results of statistical tests of alternative topologies.** Significant tests (P<0.05) are highlighted in bold font.
(DOCX)

Table S3 **Performance of ITS2 and *RPL27a* in resolving phylogenetic relationships under default (Figures S17, S20) and relaxed-GBlocks (Figures S18, S21) cleaning.** Topologies obtained without GBlocks cleaning (Figures S16, S19) are taken as references.
(DOCX)

Appendix S1 **Diagnosis of *Seres* in its new delimitation and complete list of *Seres* species with their species-group assignation.**
(DOC)

Figure S1 **Trees from a) the ML and b) Bayesian analyses of the combined dataset aligned using ClustalW and 5 partitions.** Likelihood bootstrap values and Posterior probabilities are indicated at nodes.
(PDF)

Figure S2 **Trees from a) the ML and b) Bayesian analyses of the combined dataset aligned using ClustalW and 6 partitions.** Likelihood bootstrap values and Posterior probabilities are indicated at nodes.
(PDF)

Figure S3 **Trees from a) the ML and b) Bayesian analyses of the combined dataset aligned using ClustalW + Gblocks (default parameters) and 5 partitions.** Likelihood bootstrap values and Posterior probabilities are indicated at nodes.
(PDF)

Figure S4 **Trees from a) the ML and b) Bayesian analyses of the combined dataset aligned using ClustalW + Gblocks (default parameters) and 6 partitions.** Likelihood bootstrap values and Posterior probabilities are indicated at nodes.
(PDF)

Figure S5 **Trees from a) the ML and b) Bayesian analyses of the combined dataset aligned using ClustalW + Gblocks (relaxed parameters) and 5 partitions.** Likelihood bootstrap values and Posterior probabilities are indicated at nodes.
(PDF)

Figure S6 **Trees from a) the ML and b) Bayesian analyses of the combined dataset aligned using ClustalW + Gblocks (relaxed parameters) and 6 partitions.** Likelihood bootstrap values and Posterior probabilities are indicated at nodes.
(PDF)

Figure S7 **Trees from a) the ML and b) Bayesian analyses of the combined dataset aligned using MAFFT and 5 partitions.** Likelihood bootstrap values and Posterior probabilities are indicated at nodes.
(PDF)

Figure S8 **Trees from a) the ML and b) Bayesian analyses of the combined dataset aligned using MAFFT and 6 partitions.** Likelihood bootstrap values and Posterior probabilities are indicated at nodes.
(PDF)

Figure S9 **Trees from a) the ML and b) Bayesian analyses of the combined dataset aligned using MAFFT + Gblocks (default parameters) and 5 partitions.** Likelihood bootstrap values and Posterior probabilities are indicated at nodes.
(PDF)

Figure S10 **Trees from a) the ML and b) Bayesian analyses of the combined dataset aligned using MAFFT + Gblocks (default parameters) and 6 partitions.** Likelihood bootstrap values and Posterior probabilities are indicated at nodes.
(PDF)

Figure S11 **Trees from a) the ML and b) Bayesian analyses of the combined dataset aligned using MAFFT + Gblocks (relaxed parameters) and 5 partitions.** Likelihood bootstrap values and Posterior probabilities are indicated at nodes.
(PDF)

Figure S12 **Trees from a) the ML and b) Bayesian analyses of the combined dataset aligned using MAFFT + Gblocks (relaxed parameters) and 6 partitions.** Likelihood bootstrap values and Posterior probabilities are indicated at nodes.
(PDF)

Figure S13 **Tree from the ML analysis of the mitochondrial partition.** Likelihood bootstrap values are indicated at nodes (1000 replicates).
(PDF)

Figure S14 **Tree from the ML analysis of the EF-1*α* gene region.** Likelihood bootstrap values are indicated at nodes (1000 replicates).
(PDF)

Figure S15 **Tree from the ML analysis of the *magonashi* gene region.** Likelihood bootstrap values are indicated at nodes (1000 replicates).
(PDF)

Figure S16 **Tree from the ML analysis of the ITS2 gene region (ClustalW alignment).** Likelihood bootstrap values are indicated at nodes (1000 replicates).
(PDF)

Figure S17 **Tree from the ML analysis of the ITS2 gene region (ClustalW alignment + Gblocks default parameters).** Likelihood bootstrap values are indicated at nodes (1000 replicates).
(PDF)

Figure S18 **Tree from the ML analysis of the ITS2 gene region (ClustalW alignment + Gblocks relaxed parameters).** Likelihood bootstrap values are indicated at nodes (1000 replicates).
(PDF)

Figure S19 **Tree from the ML analysis of the *RpL27a* gene region (ClustalW alignment).** Likelihood bootstrap values are indicated at nodes (1000 replicates).
(PDF)

Figure S20 **Tree from the ML analysis of the *RpL27a* gene region (ClustalW alignment + Gblocks default parameters).** Likelihood bootstrap values are indicated at nodes (1000 replicates).
(PDF)

Figure S21 **Tree from the ML analysis of the *RpL27a* gene region (ClustalW alignment + Gblocks relaxed parameters).** Likelihood bootstrap values are indicated at nodes (1000 replicates).
(PDF)

Figure S22 **Per site phylogenetic informativeness profiles of the markers based on the MAFFT + Gblocks relaxed parameters dataset.** Uppercase letters refer to clades discussed in the text (see also Figures 1 & 2).
(PDF)

Figure S23 **Per site phylogenetic informativeness profiles of the markers based on the MAFFT + Gblocks default parameters dataset.** Uppercase letters refer to clades discussed in the text (see also Figures 1 & 2).
(PDF)
