# Supplementary material for: A Multilocus Phylogeny of the World Sycoecinae Fig Wasps (Chalcidoidea: Pteromalidae)
Source: PLoS One. 2013 Nov 5;8(11):e79291. doi: 10.1371/journal.pone.0079291 (PMC3818460; doi:10.1371/journal.pone.0079291)
Supplement: Table S1 — GenBank accession numbers. (PDF) [file pone.0079291.s001.pdf]

Table S1 Genbank accession numbers for the sycoecine and outgroup species used in this study.

voucher number	genus	species	host	EF-1a	ITS2	mago nashi	Cytb	COI	RPL27a
0625_01	*Seres*	sp. nov. 1	*F. polita*	JQ756828	JQ756755	JQ756683		JQ756538	
1443_02	*Diaziella*	*bizzarea*	*F. glaberrima*	JQ756829	JQ756756	JQ756684	JQ756608	JQ756539	
1587_02	*Diaziella*	*yangi*	*F. curtipes*	JQ756830		JQ756685		JQ756540	JQ756903
1813_03	*Philocaenus*	*bouceki*	*F. reflexa*	JQ756831	JQ756757	JQ756686	JQ756609		JQ756904
1855_12	*Diaziella*	sp. nov. 1	*F. sundaica*	JQ756832	JQ756758	JQ756687			JQ756905
1855_13	*Diaziella*	sp. nov. 2	*F. sundaica*	JQ756833	JQ756759	JQ756688		JQ756541	JQ756906
1855_14	*Diaziella*	sp. nov. 3	*F. sundaica*	JQ756834	JQ756760	JQ756689	JQ756610	JQ756542	JQ756907
1877_01	*Diaziella*	sp. nov. 4	*F. sumatrana*	JQ756835	JQ756761		JQ756611	JQ756543	
1877_02	*Diaziella*	sp. nov. 5	*F. sumatrana*	JQ756836	JQ756762	JQ756690	JQ756612	JQ756544	JQ756908
1877_03	*Diaziella*	sp. nov. 6	*F. sumatrana*	JQ756837	JQ756763	JQ756691	JQ756613		JQ756909
1877_04	*Diaziella*	sp. nov. 7	*F. sumatrana*	JQ756838	JQ756764	JQ756692	JQ756614	JQ756545	JQ756910
1877_05	*Diaziella*	sp. nov. 8	*F. sumatrana*	JQ756839	JQ756765	JQ756693	JQ756615	JQ756546	JQ756911
1877_06	*Diaziella*	sp. nov. 9	*F. sumatrana*	JQ756840	JQ756766	JQ756694	JQ756616	JQ756547	JQ756912
1877_07	*Diaziella*	sp. nov. 10	*F. sumatrana*	JQ756841	JQ756767	JQ756695	JQ756617	JQ756548	JQ756913
1877_08	*Diaziella*	sp. nov. 11	*F. sumatrana*	JQ756842	JQ756768	JQ756696	JQ756618	JQ756549	JQ756914
1930_02	*Seres*	*armipes*	*F. ovata*	JQ756843	JQ756769	JQ756697	JQ756619	JQ756550	JQ756915
1936_02	*Seres*	*wardi*	*F. bubu*	JQ756844	JQ756770	JQ756698		JQ756551	JQ756916
1937_03	*Philocaenus*	sp.	*F. glumosa*	JQ756845	JQ756771	JQ756699	JQ756620	JQ756552	JQ756917
1937_04	*Crossogaster*	sp.	*F. glumosa*	JQ756846		JQ756700	JQ756621		
1945_02	*Seres*	sp. nov. 1	*F. polita*	JQ756847	JQ756772	JQ756701			JQ756918
2189_03	*Sycoecus*	sp. nov. 6	*F. tesselata*		JQ756773	JQ756702	JQ756622	JQ756553	JQ756919
2428_05	*Micranisa*	*degastris*	*F. microcarpa*	JQ756848		JQ756703	JQ756623	JQ756554	JQ756920
2428_06	*Walkerella*	nr *kurandensis*	*F. microcarpa*	JQ756849		JQ756704	JQ756624		JQ756921
2465_02	*Philocaenus*	*bouceki*	*F. reflexa*	JQ756850	JQ756774	JQ756705	JQ756625	JQ756555	JQ756922
2475_02	*Philocaenus*	*barbarus*	*F. craterostoma*	JQ756851	JQ756775	JQ756706	JQ756626	JQ756556	JQ756923

voucher number	genus	species	host	EF-1a	ITS2	mago nashi	Cytb	COI	RPL27a
2492_02	*Grandiana*	*wassae*	*F. wassa*	JQ756852		JQ756707	JQ756627	JQ756557	JQ756924
2589_02	*Sycoecus*	sp. nov. 2	*F. cyathistipula cyathistipula*	JQ756853	JQ756776	JQ756708	JQ756628	JQ756558	JQ756925
2591_02	*Sycoecus*	*taylori*	*F. conraui*		JQ756777		JQ756629	JQ756559	
2592_02	*Sycoecus*	sp. nov. 1	*F. cyathistipula cyathistipula*	JQ756854	JQ756778				
2593_02	*Philocaenus*	*liodontus*	*F. stuhlmannii*	JQ756855	JQ756779	JQ756709	JQ756630	JQ756560	JQ756926
2593_03	*Philocaenus*	*medius*	*F. stuhlmannii*	JQ756856	JQ756780	JQ756710	JQ756631	JQ756561	JQ756927
2594_02	*Crossogaster*	*odorans*	*F. petersii*	JQ756857	JQ756781	JQ756711	JQ756632	JQ756562	JQ756928
2594_03	*Philocaenus*	*liodontus*	*F. petersii*		JQ756782		JQ756633	JQ756563	
2595_02	*Philocaenus*	*barbarus*	*F. stuhlmannii*		JQ756783		JQ756634	JQ756564	
2596_02	*Crossogaster*	*odorans*	*F. stuhlmannii*		JQ756784		JQ756635		
2602_02	*Philocaenus*	*silvestrii*	*F. lutea*	JQ756858	JQ756785	JQ756712	JQ756636		
2603_02	*Philocaenus*	*silvestrii*	*F. lutea*	JQ756859	JQ756786	JQ756713	JQ756637		
2605_02	*Seres*	*solweziensis*	*F. sansibarica sansibarica*	JQ756860	JQ756787	JQ756714	JQ756638	JQ756565	JQ756929
2606_02	*Seres*	*solweziensis*	*F. sansibarica sansibarica*	JQ756861	JQ756788	JQ756715	JQ756639	JQ756566	JQ756930
2607_02	*Crossogaster*	*inusitata*	*F. sansibarica macrosperma*	JQ756862	JQ756789		JQ756640	JQ756567	
2607_03	*Seres*	sp. nov. 2	*F. sansibarica macrosperma*	JQ756863	JQ756790	JQ756716	JQ756641	JQ756568	JQ756931
2611_02	*Seres*	*solweziensis*	*F. ovata*	JQ756864	JQ756791	JQ756717	JQ756642	JQ756569	JQ756932
2612_02	*Crossogaster*	sp. nov. 2	*F. chirindensis*		JQ756792		JQ756643	JQ756570	

voucher number	genus	species	host	EF-1a	ITS2	mago nashi	Cytb	COI	RPL27a
2614_02	*Philocaenus*	*levis*	*F. ottonifolia lucanda*		JQ756793		JQ756644	JQ756571	
2615_02	*Crossogaster*	sp. nov. 1	*F. bizanae*	JQ756865			JQ756645	JQ756572	JQ756933
2616_02	*Crossogaster*	sp. nov. 1	*F. bizanae*	JQ756866	JQ756794	JQ756718	JQ756646	JQ756573	JQ756934
2617_02	*Philocaenus*	*barbarus*	*F. natalensis graniticola*	JQ756867	JQ756795	JQ756719	JQ756647	JQ756574	JQ756935
2619_02	*Philocaenus*	*liodontus*	*F. burtt-davyii*	JQ756868	JQ756796	JQ756720	JQ756648	JQ756575	JQ756936
2622_02	*Crossogaster*	*robertsoni*	*F. trichopoda*	JQ756869	JQ756797	JQ756721		JQ756576	JQ756937
2622_03	*Philocaenus*	*hippopotamus*	*F. trichopoda*	JQ756870	JQ756798	JQ756722	JQ756649	JQ756577	JQ756938
2623_02	*Philocaenus*	*rotundus*	*F. abutilifolia*	JQ756871	JQ756799	JQ756723	JQ756650		JQ756939
2625_02	*Philocaenus*	*rotundus*	*F. abutilifolia*	JQ756872	JQ756800	JQ756724	JQ756651	JQ756578	JQ756940
2628_02	*Philocaenus*	*warei*	*F. glumosa*	JQ756873	JQ756801	JQ756725	JQ756652	JQ756579	
2629_02	*Crossogaster*	*quadrata*	*F. glumosa*	JQ756874	JQ756802	JQ756726	JQ756653	JQ756580	JQ756941
2629_03	*Crossogaster*	*stigma*	*F. glumosa*	JQ756875	JQ756803		JQ756654	JQ756581	JQ756942
2629_04	*Philocaenus*	*warei*	*F. glumosa*	JQ756876	JQ756804	JQ756727	JQ756655	JQ756582	JQ756943
2632_02	*Philocaenus*	*barbarus*	*F. natalensis natalensis*	JQ756877	JQ756805	JQ756728	JQ756656	JQ756583	JQ756944
2633_02	*Philocaenus*	sp. nov. 1	*F. usambarensis*	JQ756878	JQ756806	JQ756729		JQ756584	JQ756945
2637_02	*Crossogaster*	*odorans*	*F. burkei*	JQ756879			JQ756657	JQ756585	JQ756946
2637_03	*Philocaenus*	*barbarus*	*F. burkei*	JQ756880		JQ756730	JQ756658	JQ756586	JQ756947
2639_02	*Philocaenus*	*barbarus*	*F. burkei*		JQ756807		JQ756659	JQ756587	
2640_02	*Crossogaster*	*odorans*	*F. natalensis natalensis*	JQ756881	JQ756808	JQ756731	JQ756660	JQ756588	JQ756948
2640_03	*Philocaenus*	*liodontus*	*F. natalensis natalensis*	JQ756882	JQ756809	JQ756732	JQ756661	JQ756589	JQ756949

voucher number	genus	species	host	EF-1a	ITS2	mago nashi	Cytb	COI	RPL27a
2640_04	*Philocaenus*	*medius*	*F. natalensis natalensis*		JQ756810		JQ756662	JQ756590	
2642_02	*Crossogaster*	*odorans*	*F. petersii*	JQ756883	JQ756811	JQ756733	JQ756663	JQ756591	JQ756950
2642_03	*Philocaenus*	*barbarus*	*F. petersii*	JQ756884	JQ756812	JQ756734	JQ756664	JQ756592	JQ756951
2642_04	*Philocaenus*	*liodontus*	*F. petersii*	JQ756885	JQ756813	JQ756735	JQ756665	JQ756593	
2645_02	*Crossogaster*	sp. nov. 6	*F. louisii*	JQ756886	JQ756814	JQ756736	JQ756666	JQ756594	JQ756952
2902_01	*Robertsia*	sp.	*F. xylosycia*		JQ756815	JQ756737			JQ756953
2908_01	*Diaziella*	sp.	*F. lawesii*		JQ756816	JQ756738			JQ756954
2958_02	*Philocaenus*	*medius*	*F. natalensis natalensis*	JQ756887	JQ756817	JQ756739	JQ756667	JQ756595	JQ756955
2962_01	*Megastigmus*	*aculeatus*	N/A	JQ756888		JQ756740	JQ756668	JQ756596	
2965_02	*Philocaenus*	sp. nov. 2	*F. wakefieldi*	JQ756889	JQ756818	JQ756741	JQ756669	JQ756597	JQ756956
2968_01	*Crossogaster*	sp. nov. 2	*F. chirindensis*	JQ756890	JQ756819	JQ756742	JQ756670	JQ756598	JQ756957
2968_02	*Crossogaster*	sp. nov. 3	*F. chirindensis*	JQ756891	JQ756820	JQ756743	JQ756671	JQ756599	JQ756958
2969_02	*Sycoecus*	sp. nov. 5	*F.* nr *barteri*	JQ756892	JQ756821	JQ756744	JQ756672	JQ756600	JQ756959
2970_02	*Sycoecus*	sp. nov. 3	*F. densistipulata*	JQ756893		JQ756745	JQ756673	JQ756601	JQ756960
2971_02	*Sycoecus*	*taylori*	*F. conraui*	JQ756894	JQ756822	JQ756746	JQ756674	JQ756602	JQ756961
2972_02	*Crossogaster*	sp. nov. 2	*F. chirindensis*	JQ756895	JQ756823	JQ756747	JQ756675	JQ756603	JQ756962
2974_02	*Crossogaster*	*michaloudi*	*F. artocarpoides*	JQ756896		JQ756748			JQ756963
2975_03	*Philoceanus*	*medius*	*F. natalensis*	JQ756897	JQ756824	JQ756749	JQ756676	JQ756604	JQ756964
2975_04	*Crossogaster*	sp. nov. 4	*F. natalensis*	JQ756898	JQ756825	JQ756750	JQ756677		JQ756965

voucher number	genus	species	host	EF-1a	ITS2	mago nashi	Cytb	COI	RPL27a
2977_04	*Crossogaster*	sp. nov. 5	*F.* sp. nov. nr *polita/umbellata*	JQ756899		JQ756751	JQ756678		JQ756966
2978_02	*Sycoecus*	sp. nov. 4	*F.* nr *barteri*	JQ756900	JQ756826	JQ756752	JQ756679		JQ756967
2989_01	*Diaziella*	sp. nov. 12	*F.* sp.		JQ756827		JQ756680	JQ756605	
GDEL0327	*Haltichella*	*rufipes*	N/A	JQ756901		JQ756753	JQ756681	JQ756606	
GDEL1288	*Bruchophagus*	*caucasicus*	N/A	JQ756902		JQ756754	JQ756682	JQ756607	
