# Supplementary material for: A Multilocus Phylogeny of the World Sycoecinae Fig Wasps (Chalcidoidea: Pteromalidae)
Source: PLoS One. 2013 Nov 5;8(11):e79291. doi: 10.1371/journal.pone.0079291 (PMC3818460; doi:10.1371/journal.pone.0079291)
Supplement: Table S2 — Results of statistical tests of alternative topologies. Significant tests (P<0.05) are highlighted in bold font. (DOCX) [file pone.0079291.s002.docx]

**Table S2.** Results of statistical tests of alternative topologies. Significant tests (P<0.05) are highlighted in bold font.

| dataset | best topology | alternative topology | Difference  (-ln *L*) | AU test  *P* value | SH test  *P* value |  |
| --- | --- | --- | --- | --- | --- | --- |
| combined dataset, aligned with MAFFT | MAFFT, ML tree [6 partitions] | ClustalW, ML tree [6 partitions] | 10.7 | 0.218 | 0.861 | 1 |
|  |  | ClustalW, Bayesian tree [6 partitions] | 11.2 | 0.247 | 0.870 | 2 |
|  |  | ClustalW, ML tree [5 partitions] | 16.7 | 0.287 | 0.752 | 3 |
|  |  | ClustalW, Bayesian tree [5 partitions] | 15.3 | 0.140 | 0.800 | 4 |
|  |  | ClustalW + Gblocks default, ML tree [5 partitions] | 95.3 | **0.001** | **0.018** | 5 |
|  |  | ClustalW + Gblocks default, Bayesian tree [5 partitions] | 83.6 | **0.003** | **0.025** | 6 |
|  |  | ClustalW + Gblocks default, ML tree [6 partitions] | 111.9 | **<0.001** | **0.006** | 7 |
|  |  | ClustalW + Gblocks default, Bayesian tree [6 partitions] | 87.8 | **0.010** | **0.023** | 8 |
|  |  | ClustalW + Gblocks relaxed, ML tree [5 partitions] | 72.3 | **0.030** | 0.069 | 9 |
|  |  | ClustalW + Gblocks relaxed, Bayesian tree [5 partitions] | 63.3 | 0.054 | 0.117 | 10 |
|  |  | ClustalW + Gblocks relaxed, ML tree [6 partitions] | 57.8 | 0.074 | 0.149 | 11 |
|  |  | ClustalW + Gblocks relaxed, Bayesian tree [6 partitions] | 22.1 | 0.100 | 0.698 | 12 |
|  |  | MAFFT, ML tree [5 partitions] | 13.2 | 0.410 | 0.804 | 13 |
|  |  | MAFFT, Bayesian tree [5 partitions] | 7.7 | 0.417 | 0.929 | 14 |
|  |  | MAFFT, Bayesian tree [6 partitions] | 2.4 | 0.742 | 0.980 | 16 |
|  |  | MAFFT + Gblocks default, ML tree [5 partitions] | 79.3 | **0.016** | **0.047** | 17 |
|  |  | MAFFT + Gblocks default, Bayesian tree [5 partitions] | 65.7 | **0.007** | **0.043** | 18 |
|  |  | MAFFT + Gblocks default, ML tree [6 partitions] | 100.4 | **0.001** | **0.007** | 19 |
|  |  | MAFFT + Gblocks default, Bayesian tree [6 partitions] | 75.9 | **0.005** | **0.033** | 20 |
|  |  | MAFFT + Gblocks relaxed, ML tree [5 partitions] | 39.2 | **0.047** | 0.359 | 21 |
|  |  | MAFFT + Gblocks relaxed, Bayesian tree [5 partitions] | 11.0 | 0.219 | 0.885 | 22 |
|  |  | MAFFT + Gblocks relaxed, ML tree [6 partitions] | 29.2 | 0.102 | 0.507 | 23 |
|  |  | MAFFT + Gblocks relaxed, Bayesian tree [6 partitions] | 10.7 | 0.366 | 0.876 | 24 |
|  |  | Sycoecinae constrained to be monophyletic, MAFFT, ML tree [6 partitions] | 3.9 | 0.458 | 0.958 | 25 |
|  |  | *Diaziella* constrained to be sister to all other Sycoecinae, including *Robertsia*, MAFFT, ML tree [6 partitions] | 8.4 | 0.116 | 0.901 | 26 |
|  |  | *Seres* constrained to be monophyletic, MAFFT, ML tree [6 partitions] | 80.2 | **<0.001** | **0.047** | 27 |
