# Supplementary material for: A Multilocus Phylogeny of the World Sycoecinae Fig Wasps (Chalcidoidea: Pteromalidae)
Source: PLoS One. 2013 Nov 5;8(11):e79291. doi: 10.1371/journal.pone.0079291 (PMC3818460; doi:10.1371/journal.pone.0079291)
Supplement: Table S3 — Performance of ITS2 and RPL27a in resolving phylogenetic relationships under default (Figures S17, S20) and relaxed-GBlocks (Figures S18, S21) cleaning. Topologies obtained without GBlocks cleaning (Figures S16, S19) are taken as references. (DOCX) [file pone.0079291.s003.docx]

**Table S3.** Performance of ITS2 and *RPL27a* in resolving phylogenetic relationships under default (Figs S17, S20) and relaxed-GBlock (Figs S18, S21) cleaning. Topologies obtained without GBlock cleaning (Figs S16, S19) are taken as references.

|  | **Default-GBlock topology** | **Relaxed-GBlock topology** |
| --- | --- | --- |
| **ITS2** | *Shallower nodes:*  Shorter branches, lots of polytomies, clades less resolved (e.g. *Diaziella* ex *F. sundaica* clade, C4.5, B2), some well-supported clades disappeared (C4.1, C4.2, C4.4) | *Shallower nodes:*  Branches a bit shorter, a few polytomies (e.g. in C4.5, C2). |
|  | *Intermediate nodes:*  Clades C1, C2, C4 disappeared | *Intermediate nodes:*  Relationships within clade C changed but all were unsupported in the reference tree |
|  | *Deeper nodes:*  Backbone of the tree unresolved, Clade C not monophyletic | *Deeper nodes:*  Unchanged, high Bootstrap supports |
| ***RPL27a*** | *Shallower nodes:*  Branches globally shorter, clades less resolved (e.g. *P. barbarous* clade, *C. odorans* clade, *Diaziella* ex *F. sundaica* clade), some well-supported clades disappeared (e.g. C4.2) | *Shallower nodes:*  No significant differences |
|  | *Intermediate nodes:*  Highly-supported clade C2 disappeared | *Intermediate nodes:*  No significant differences |
|  | *Deeper nodes:*  Not much differences, Bootstrap supports decreased | *Deeper nodes:*  No significant differences well resolved and highly supported. |
