# Supplementary material for: A Multilocus Phylogeny of the World Sycoecinae Fig Wasps (Chalcidoidea: Pteromalidae)
Source: PLoS One. 2013 Nov 5;8(11):e79291. doi: 10.1371/journal.pone.0079291 (PMC3818460; doi:10.1371/journal.pone.0079291)
Supplement: Appendix S1 — Diagnosis of Seres in its new delimitation and complete list of Seres species with their species-group assignation. (DOC) [file pone.0079291.s004.doc]

**Appendix S1. Diagnosis of *Seres* in its new delimitation and complete list of *Seres* species with their species-group assignation.**

***Seres* Waterston**

*Seres* Waterston 1919, 275-276.

Type species: *Seres armipes* Waterston 1919, by original designation.

*Philocaenus* Grandi, 1952, 38-40. Type species: *Philocaenus barbatus* Grandi, by monotypy. **Syn. nov.**

*Phagoblastus* Grandi 1955, 100-102. Type species: *Phagoblastus barbarus* Grandi, by monotypy. Synonymised with *Philocaenus* (van Noort, 1994b).

**Description**

FEMALE. Head as broad as long to distinctly elongate, slightly to extremely dorso-ventrally flattened, smooth, polished; clypeus narrow to broad, epistomal margin very variable in shape; toruli in close apposition or touching; malar sulcus either absent, present for the first quarter to third of the cheek length or for full cheek length. Compound eyes situated posteriorly on face, the temple may still be present laterally above the eyes; or centrally, large. Vertex with an occipital carina present medially, situated dorsal to a broad invagination of the ventral margin of the vertex; strong setae present. Vertex usually rounded, but temple and frons may meet the vertex at ninety degrees, such that the vertex is flat with broad, shallow, lateral excavations or else a medial prominence is present and the vertex is more rounded. Lateral ocelli situated on axial lip of excavations or on rounded vertex. Ventral tentorial pits in very close apposition, often difficult to distinguish as paired, usually situated in the posterior end of a short medial depression, ca. a third of the distance or half way between the oral fossa and the foramen magnum; sometimes distinctly separated without a medial depression present. Internally the tentorial beams diverge dorsally such that the dorsal tentorial pits are widely spaced and situated on the clypeal sutures. Hypostoma may be present or absent, mouthparts sunken such that the stipes are flush with the ventral surface of the head, cardo absent. Two labial palp segments, basal segment half to subequal the length of the distal segment. Three maxillary palp segments. Mandible very variable, with two apical teeth and the ventral armature ranging from a single tooth to a plate of many teeth.

Antennae either eleven segmented, formula 1124(3), or twelve segmented, formula 1134(3), anelli in close apposition, second larger than first. Multiporous plate sensilla (MPS) numerous, placoid, thin and elongate, present in a single row in close apposition to one another, reduced in number ventrally on funicle segments.

Mesosoma dorso-ventrally flattened, smooth, polished; pronotum transverse to elongate, laterally depressed, concave with a weak carina present on the dorso-anterior margin; mesonotum suboval to subtriangular, parapsidal sulci complete, curved; propodeal spiracles either anteriorly situated (may be surrounded by a shallow excavation) or medially situated with sulci well defined posterior to spiracles, shallower anterior to the spiracles. General form of legs typical for sycoecines, fore femur stout, elongate, subcylindrical; fore tibial armature variable: may be bidentate, a comb of teeth, or a combination of 2-3 rows of teeth. Fore tibial spur elongate, articulated half way up tibia, ventral tooth next to spur insertion may be present. Wings almost glabrous, microsetae indistinct, fringe present on forewing, longer fringe on hindwing; anal and medial setal tracts present. Postmarginal vein subequal to shorter than stigmal, marginal vein thickened.

Metasoma. Tergites either crenulated (frayed) with three deeper dorsal medial incisions or with five to seven evenly spaced incisions on the posterior edge, with a smooth edge. In the latter case the medial three incisions on first tergite are in close approximation, with the lateral two incisions closing posteriorly with the medial incision. Eighth urotergite spiracle normal, ventro-laterally situated, may have a slightly expanded peritreme. Ovipositor valves from 0.28X to half the length of the gaster.

MALE. Head ranging from broad to elongate either subquadrate or distinctly narrower anteriorly than across the eyes; broadly excavated posterio-ventrally; clypeus subtriangular in area, epistomal margin broader than in female, slightly concave. Toruli usually touching but may be separated, situated between eyes or just below or in line with the base of the eyes. Antennal formula 1124(3), with the second anellus longer than the first. Multiporous plate sensilla thin, placoid, numerically very reduced. Dorsal tentorial pits situated on lateral clypeal sutures, either closer to epistomal margin than to toruli or half way between the toruli and the epistomal margin. Strong setae present on the vertex. Malar sulcus either absent or if present then only for posterior half of cheek. Ventral tentorial pits slightly to well separated, either situated closer to the oral fossa than to the foramen magnum or half way between the foramen magnum and the oral fossa. Three maxillary palp segments, two labial palp segments. Mandible either subequal in length, with the outer tooth slightly longer or with a larger outer apical tooth and a shorter inner subapical tooth, which may be uni- or bicuspid; two glands, subapical gland smaller.

Mesosoma. Pronotum broad or torpedo shaped, with a carina present on the anterio-dorsal margin; mesonotum broad, semicircular to subtriangular, parapsidal sulci incomplete to complete, evenly curved or straight. Propodeal sulci may be present, spiracles anteriorly or medially situated. Two strong subequal dorso-apical teeth on fore tibia. Wings pilose, axial third bare, rest covered in microsetae, anal and medial setal tracts present on forewing;; fringe present. Thickened marginal vein.

Metasoma. Posterior edges of tergites straight, with strong short setae along the edge. Aedeagus small (less than half the metsoma length) to large.

**Diagnosis**. Fore tarsi five-segmented, antennal scrobe present; hypopygium not extending beyond end of metasoma; clypeal sutures present; fore tibial spur normal; 2 labial, 3 maxillary palp segments; antenna with either two or three anelli and four funicle segments (i.e. antennae either 11 or 12 segmented); first funicle segment normal; ventral tentorial pits in close apposition; eighth urotergite spiracle not expanded; forewing marginal vein thickened. *Seres* males fully winged; outer mandibular tooth longer than the inner, without any ventral teeth present.

**Complete list of *Seres* species with their species-group assignation**

***Seres armipes* species-group**

[*Seres* *armipes*](http://www.figweb.org/Fig_wasps/pteromalidae/sycoecinae/Seres/Seres_armipes_armipes.htm) *armipes* Waterston, 1919

[*Seres armipes*](http://www.figweb.org/Fig_wasps/pteromalidae/sycoecinae/Seres/Seres_armipes_breviceps.htm) *breviceps* Wiebes, 1961

[*Seres*](http://www.figweb.org/Fig_wasps/pteromalidae/sycoecinae/Seres/Seres_solweziensis.htm) *solweziensis* van Noort, 1993

***Seres barbatus* species-group**

[*Seres arrujumensis*](http://www.figweb.org/Fig_wasps/pteromalidae/sycoecinae/Philocaenus/Philocaenus_arrujumensis.htm) (van Noort, 2006) comb. nov.

[*Seres*](http://www.figweb.org/Fig_wasps/pteromalidae/sycoecinae/Philocaenus/Philocaenus_barbatus.htm) *barbatus* (Grandi, 1952) comb. nov.

[*Seres*](http://www.figweb.org/Fig_wasps/pteromalidae/sycoecinae/Philocaenus/Philocaenus_hippopotomus.htm) *hippopotomus* (van Noort, 1994) comb. nov.

***Seres levis* species-group**

[*Seres*](http://www.figweb.org/Fig_wasps/pteromalidae/sycoecinae/Philocaenus/Philocaenus_bakeri.htm) *bakeri* (van Noort, 1994) comb. nov.

[*Seres*](http://www.figweb.org/Fig_wasps/pteromalidae/sycoecinae/Philocaenus/Philocaenus_bifurcus.htm) *bifurcus* (van Noort, 1994) comb. nov.

[*Seres*](http://www.figweb.org/Fig_wasps/pteromalidae/sycoecinae/Philocaenus/Philocaenus_clairae.htm) *clairae* (van Noort, 1994) comb. nov.

[*Seres*](http://www.figweb.org/Fig_wasps/pteromalidae/sycoecinae/Philocaenus/Philocaenus_levis.htm) *levis* Waterston, 1920 comb. nov.

[*Seres*](http://www.figweb.org/Fig_wasps/pteromalidae/sycoecinae/Philocaenus/Philocaenus_rasplusi.htm) *rasplusi* (van Noort, 1994) comb. nov.

***Seres liodontus* species-group**

[*Seres*](http://www.figweb.org/Fig_wasps/pteromalidae/sycoecinae/Philocaenus/Philocaenus_barbarus.htm) *barbarus* (Grandi, 1955) comb. nov.

[*Seres*](http://www.figweb.org/Fig_wasps/pteromalidae/sycoecinae/Philocaenus/Philocaenus_bouceki.htm) *boučeki* (Wiebes, 1982) comb. nov.

[*Seres*](http://www.figweb.org/Fig_wasps/pteromalidae/sycoecinae/Philocaenus/Philocaenus_comorensis.htm) *comorensis* (van Noort, 1994) comb. nov.

[*Seres*](http://www.figweb.org/Fig_wasps/pteromalidae/sycoecinae/Philocaenus/Philocaenus_geminus.htm) *geminus* (van Noort, 1994) comb. nov.

[*Seres*](http://www.figweb.org/Fig_wasps/pteromalidae/sycoecinae/Philocaenus/Philocaenus_insolitus.htm) *insolitus* (van Noort, 1994) comb. nov.

[*Seres*](http://www.figweb.org/Fig_wasps/pteromalidae/sycoecinae/Philocaenus/Philocaenus_jinjaensis.htm) *jinjaensis* (van Noort, 1994) comb. nov.

[*Seres*](http://www.figweb.org/Fig_wasps/pteromalidae/sycoecinae/Philocaenus/Philocaenus_liodontus.htm) *liodontus* (Wiebes, 1979) comb. nov.

[*Seres*](http://www.figweb.org/Fig_wasps/pteromalidae/sycoecinae/Philocaenus/Philocaenus_medius.htm) *medius* (van Noort, 1994) comb. nov.

[*Seres*](http://www.figweb.org/Fig_wasps/pteromalidae/sycoecinae/Philocaenus/Philocaenus_quatuordentatus.htm) *quatuordentatus* (van Noort, 1994) comb. nov.

[*Seres*](http://www.figweb.org/Fig_wasps/pteromalidae/sycoecinae/Philocaenus/Philocaenus_rotundus.htm) *rotundus* (van Noort, 1994) comb. nov.

[*Seres*](http://www.figweb.org/Fig_wasps/pteromalidae/sycoecinae/Philocaenus/Philocaenus_ugandensis.htm) *ugandensis* (van Noort, 1994) comb. nov.

[*Seres*](http://www.figweb.org/Fig_wasps/pteromalidae/sycoecinae/Philocaenus/Philocaenus_warei.htm) *warei* (van Noort, 1994) comb. nov.

[*Seres*](http://www.figweb.org/Fig_wasps/pteromalidae/sycoecinae/Philocaenus/Philocaenus_zambesiacus.htm) *zambesiacus* (van Noort, 1994) comb. nov.

***Seres silvestrii* species-group**

[*Seres*](http://www.figweb.org/Fig_wasps/pteromalidae/sycoecinae/Philocaenus/Philocaenus_cavus.htm) *cavus* (van Noort, 1994) comb. nov.

[*Seres*](http://www.figweb.org/Fig_wasps/pteromalidae/sycoecinae/Philocaenus/Philocaenus_comptoni.htm) *comptoni* (van Noort, 1994) comb. nov.

[*Seres*](http://www.figweb.org/Fig_wasps/pteromalidae/sycoecinae/Philocaenus/Philocaenus_silvestrii.htm) *silvestrii* (Grandi, 1916) comb. nov.

**Species-group inquirenda**

[*Seres* *longicalcar*](http://www.figweb.org/Fig_wasps/pteromalidae/sycoecinae/Seres/Seres_longicalcar.htm) van Noort, 1993
