# Supplementary figures and images for: A Multilocus Phylogeny of the World Sycoecinae Fig Wasps (Chalcidoidea: Pteromalidae)
Source: PLoS One. 2013 Nov 5;8(11):e79291. doi: 10.1371/journal.pone.0079291 (PMC3818460; doi:10.1371/journal.pone.0079291)

Fig S1  
a)

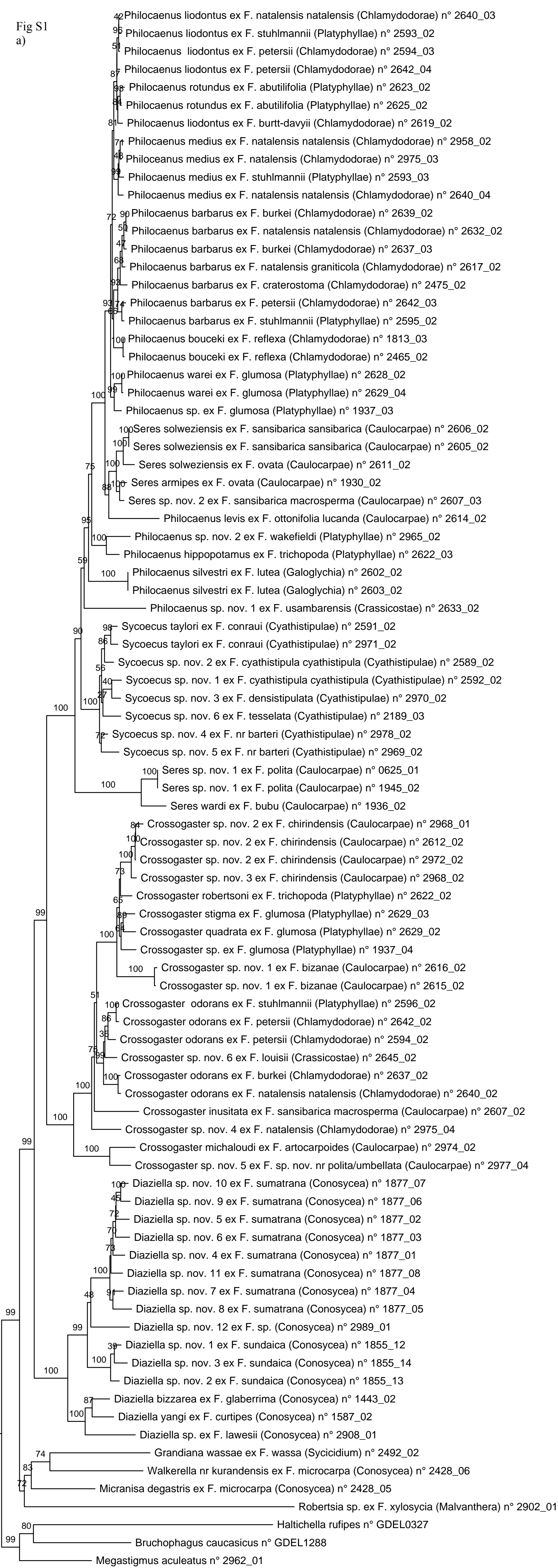

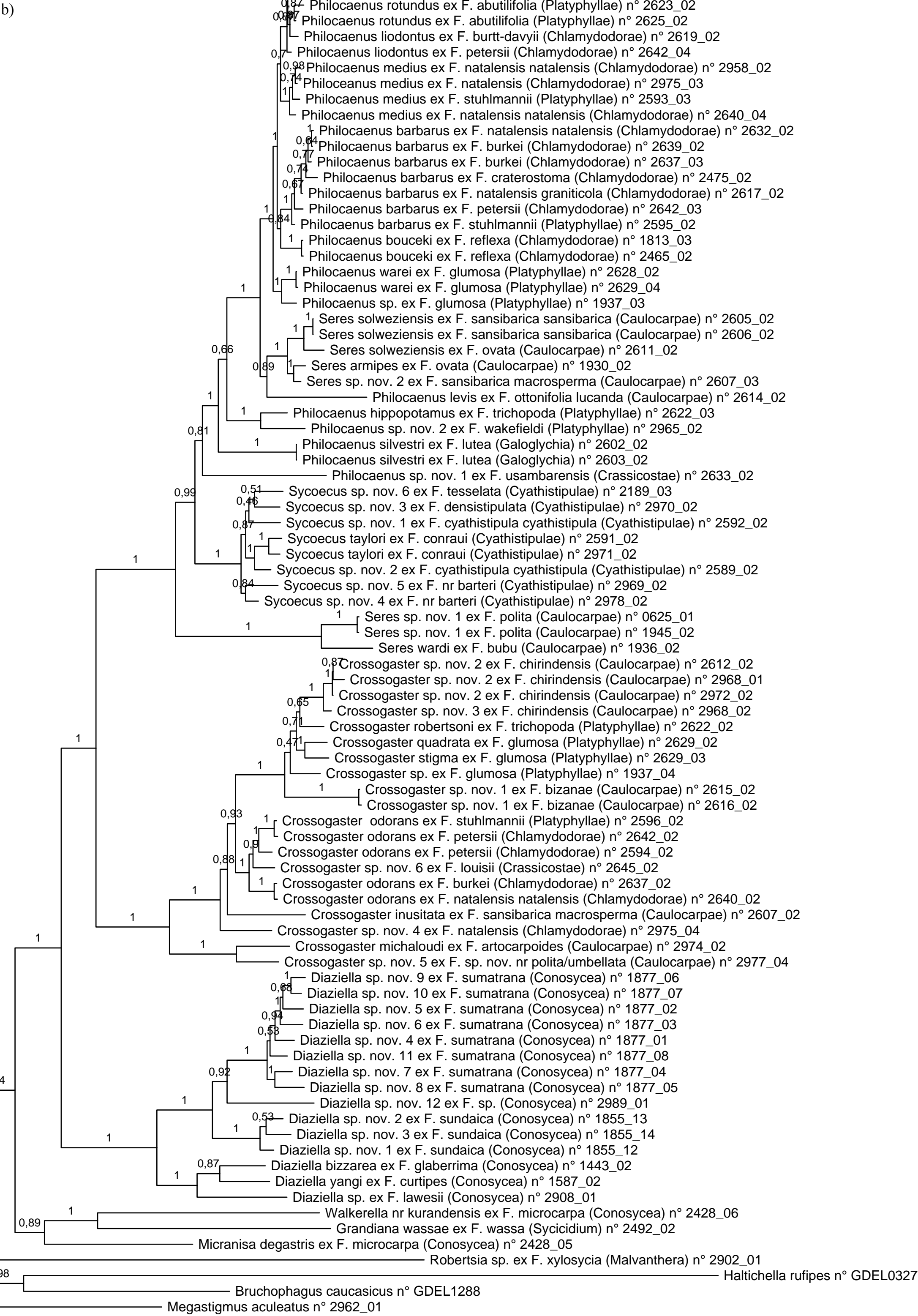

Supplement: Figure S1 — Trees from a) the ML and b) Bayesian analyses of the combined dataset aligned using ClustalW and 5 partitions. Likelihood bootstrap values and Posterior probabilities are indicated at nodes. (PDF) [file pone.0079291.s005.pdf]

Fig S2  
a)

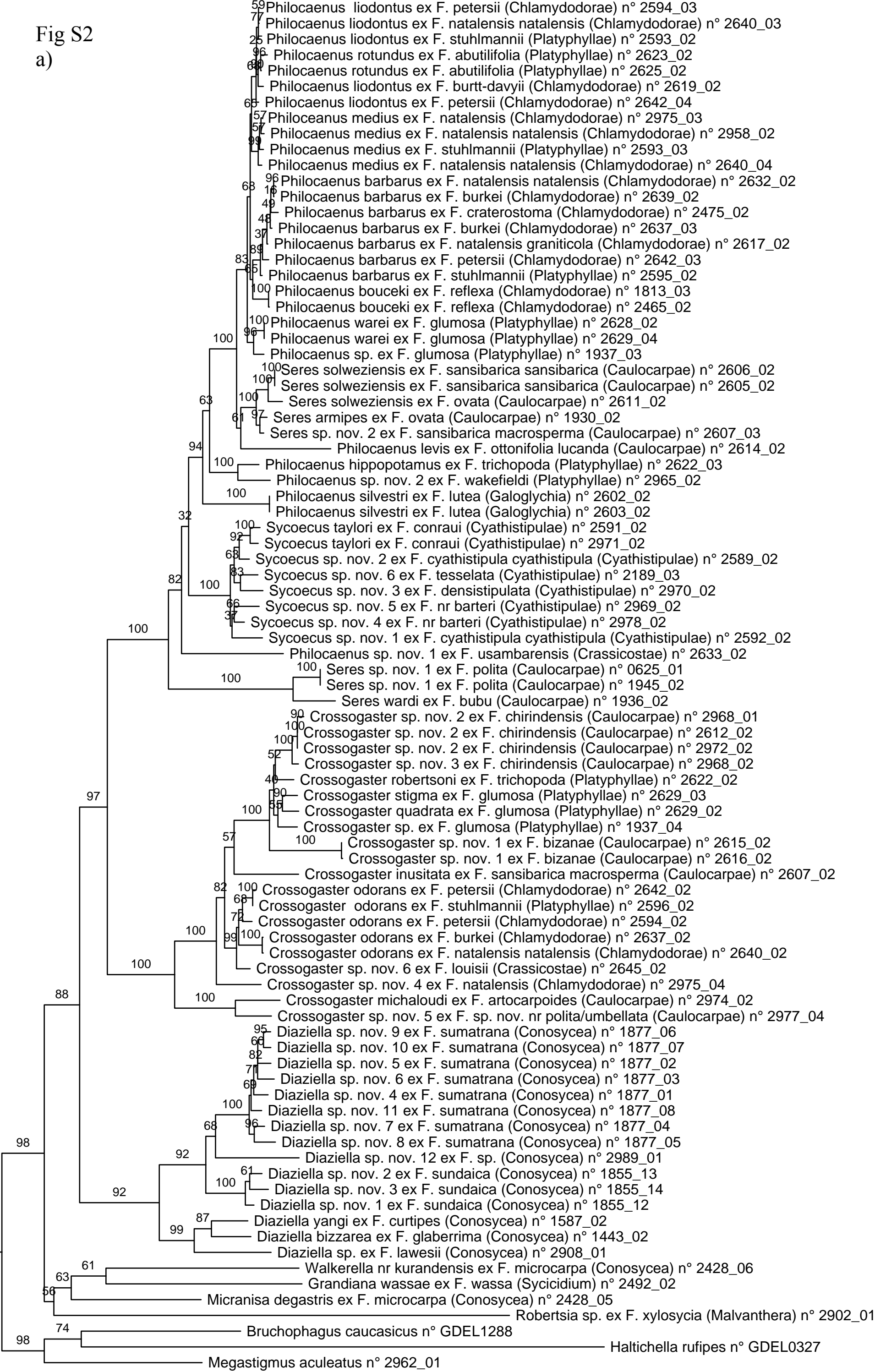

b)

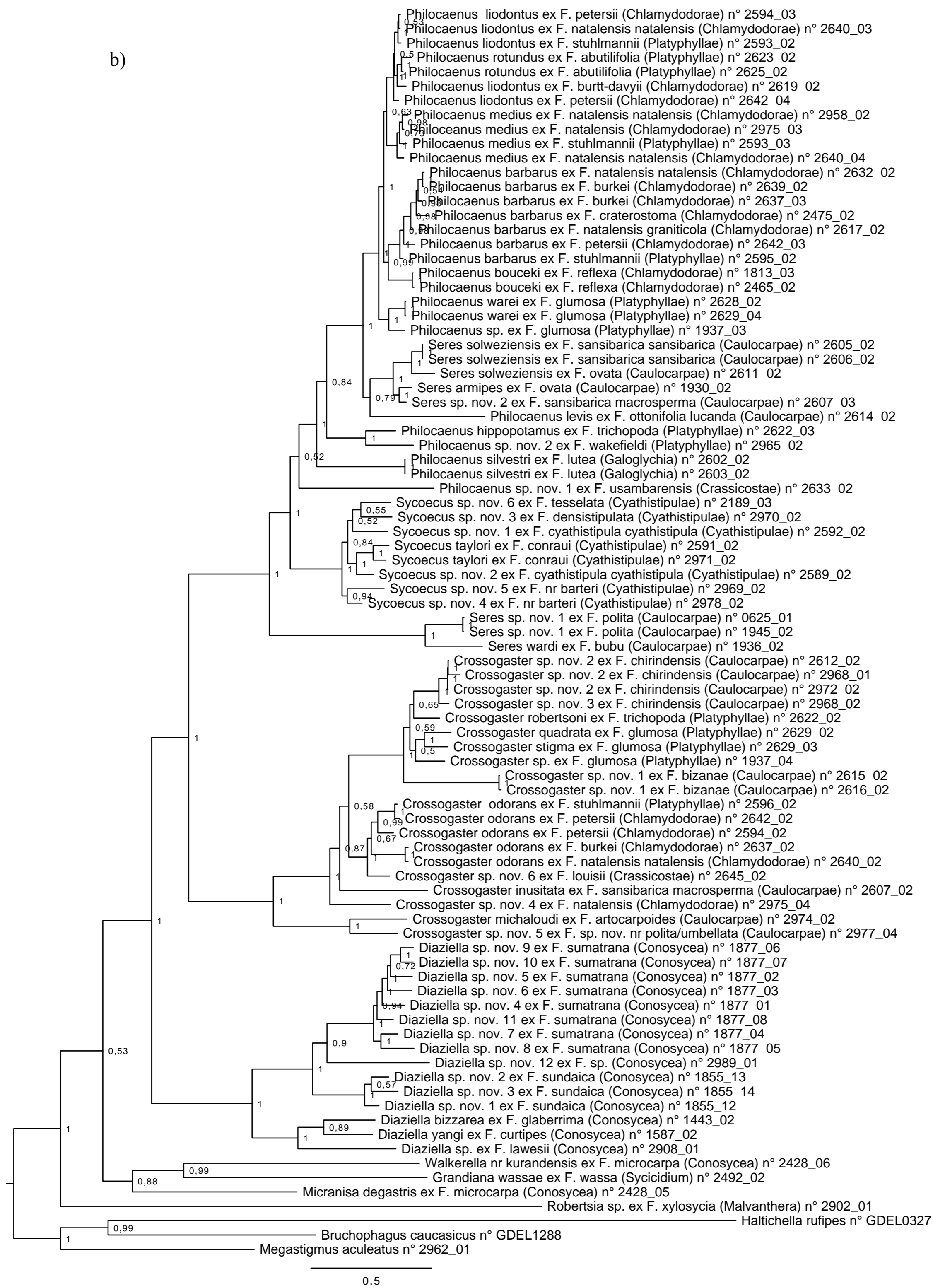

Supplement: Figure S2 — Trees from a) the ML and b) Bayesian analyses of the combined dataset aligned using ClustalW and 6 partitions. Likelihood bootstrap values and Posterior probabilities are indicated at nodes. (PDF) [file pone.0079291.s006.pdf]

Fig. S3

a)

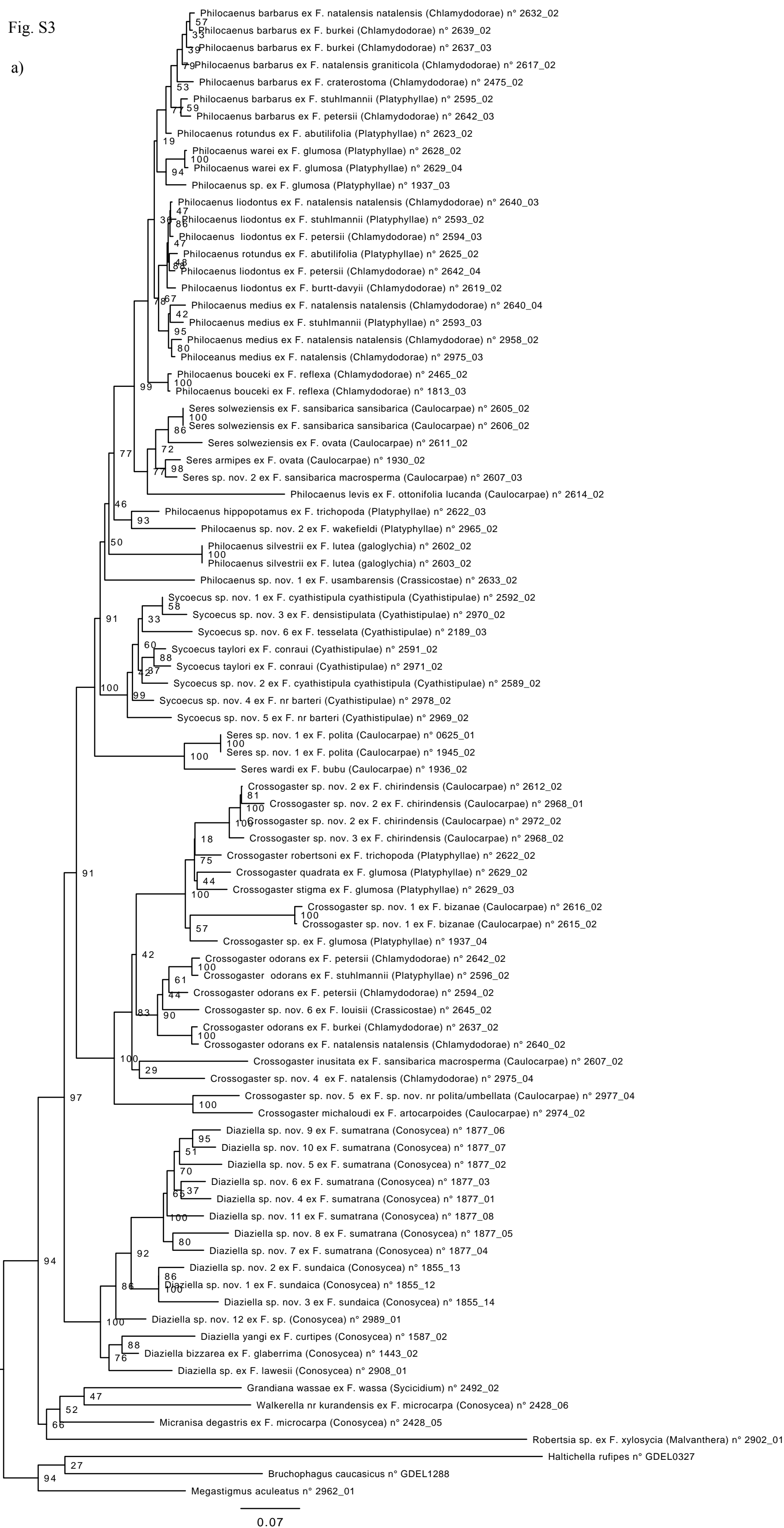

b)

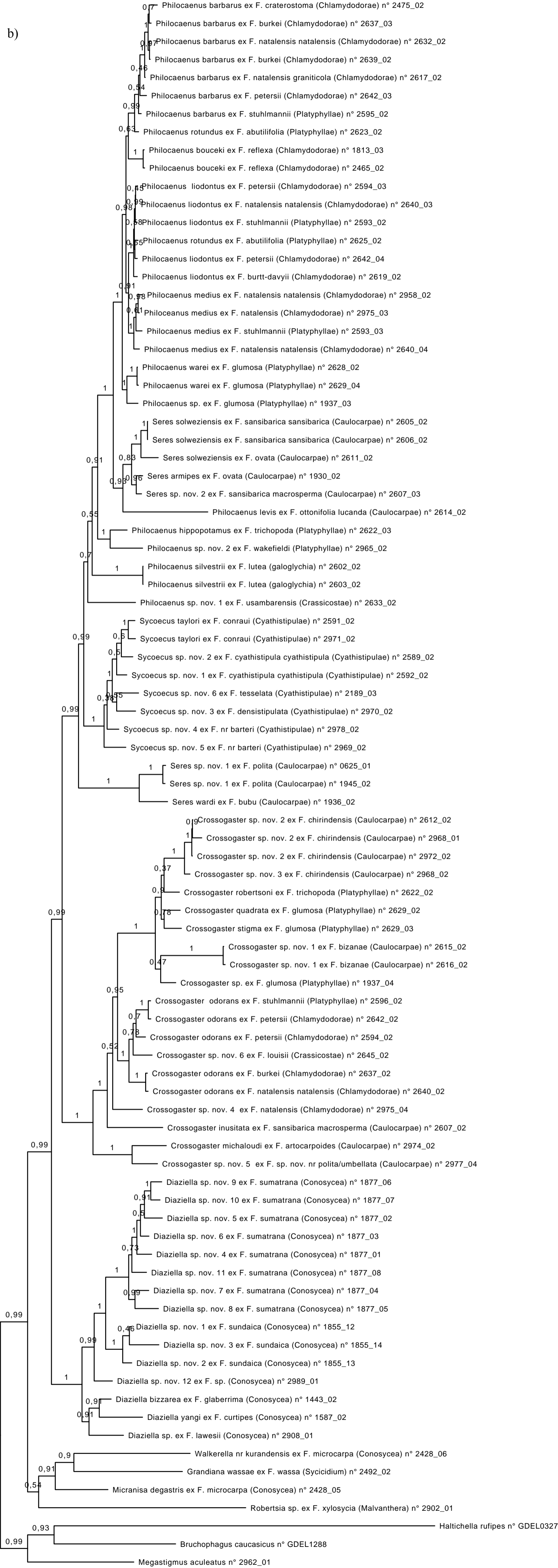

0.3

Supplement: Figure S3 — Trees from a) the ML and b) Bayesian analyses of the combined dataset aligned using ClustalW + Gblocks (default parameters) and 5 partitions. Likelihood bootstrap values and Posterior probabilities are indicated at nodes. (PDF) [file pone.0079291.s007.pdf]

Fig S4  
a)

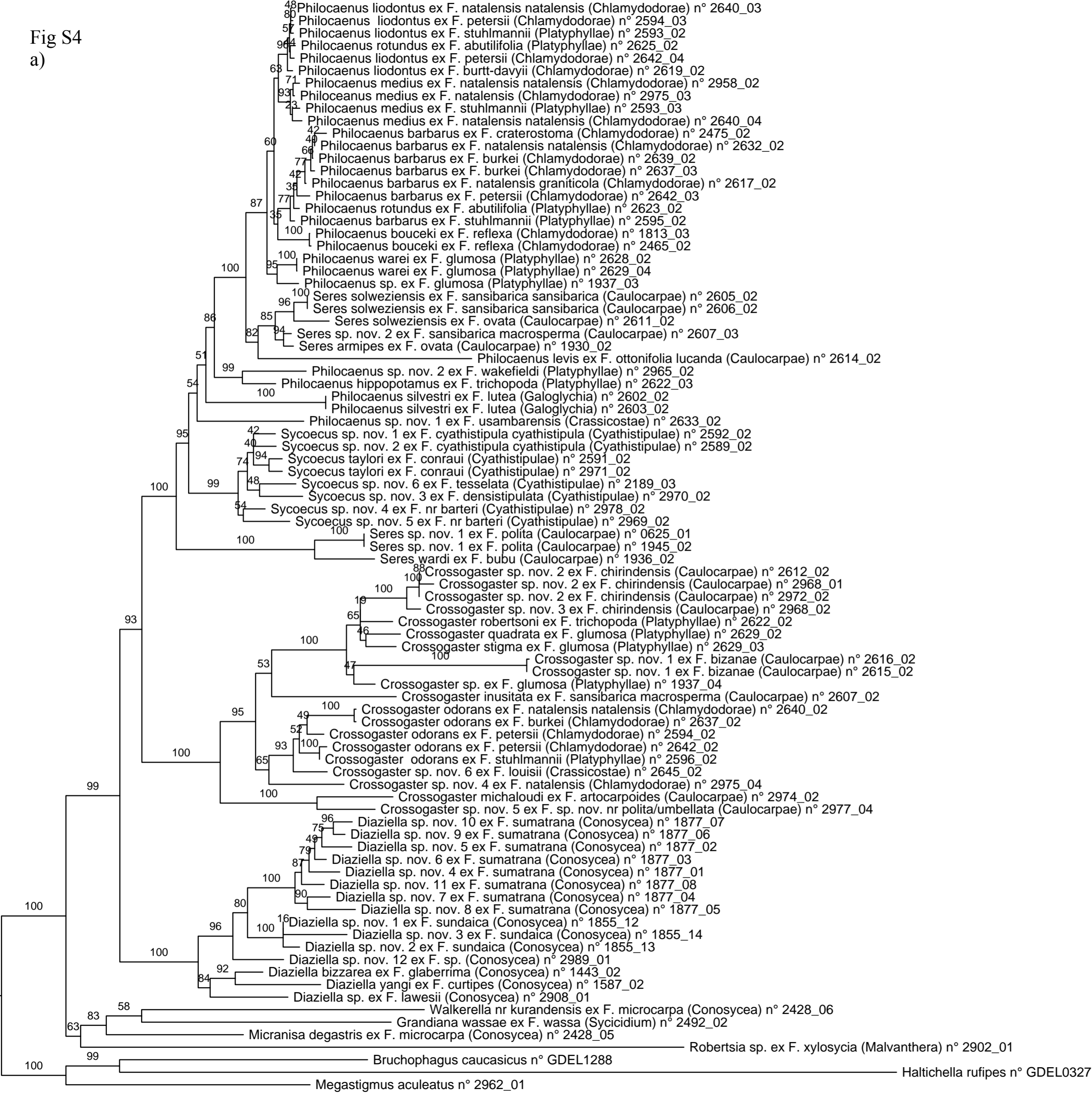

b)

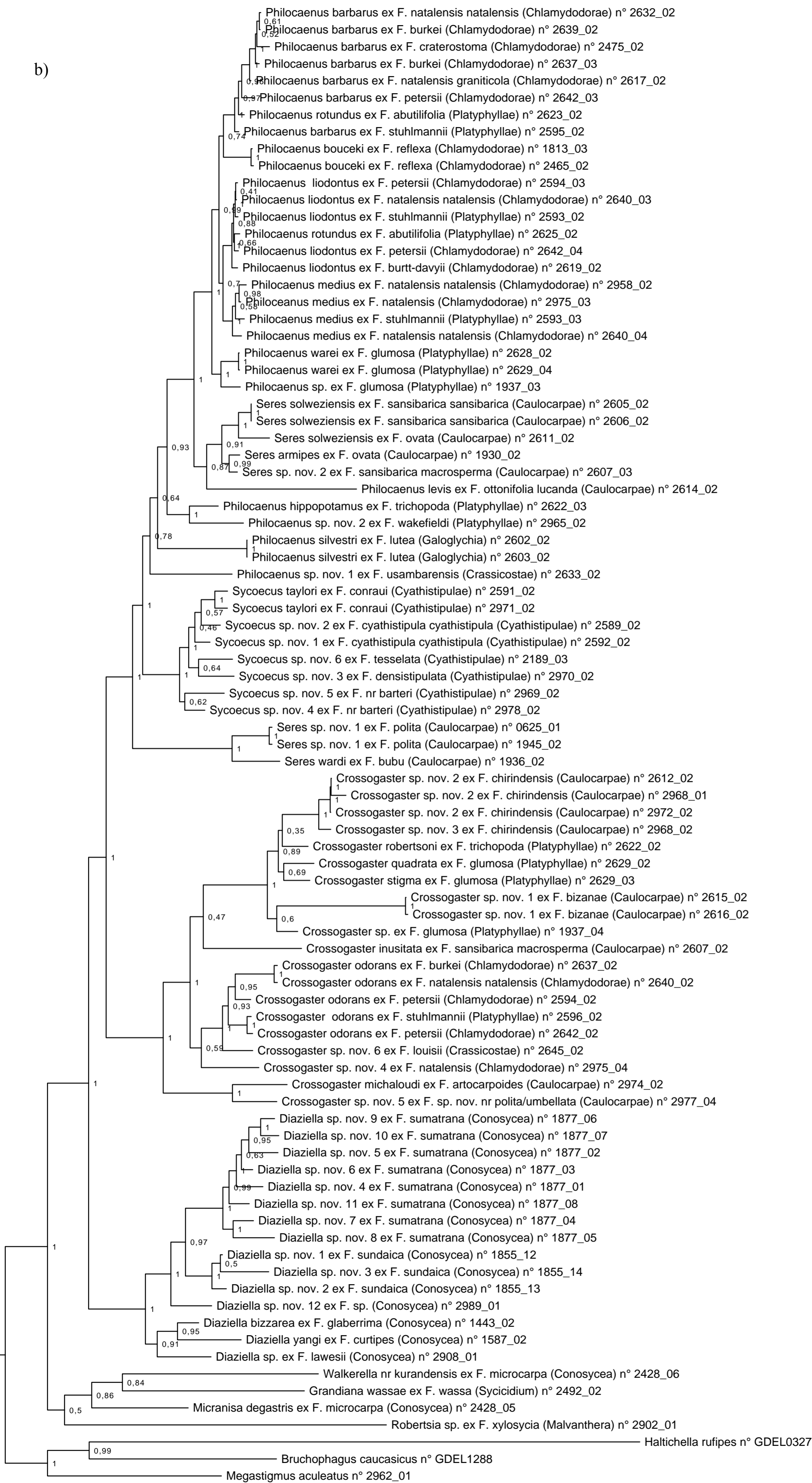

Supplement: Figure S4 — Trees from a) the ML and b) Bayesian analyses of the combined dataset aligned using ClustalW + Gblocks (default parameters) and 6 partitions. Likelihood bootstrap values and Posterior probabilities are indicated at nodes. (PDF) [file pone.0079291.s008.pdf]

Fig. S5  
a)

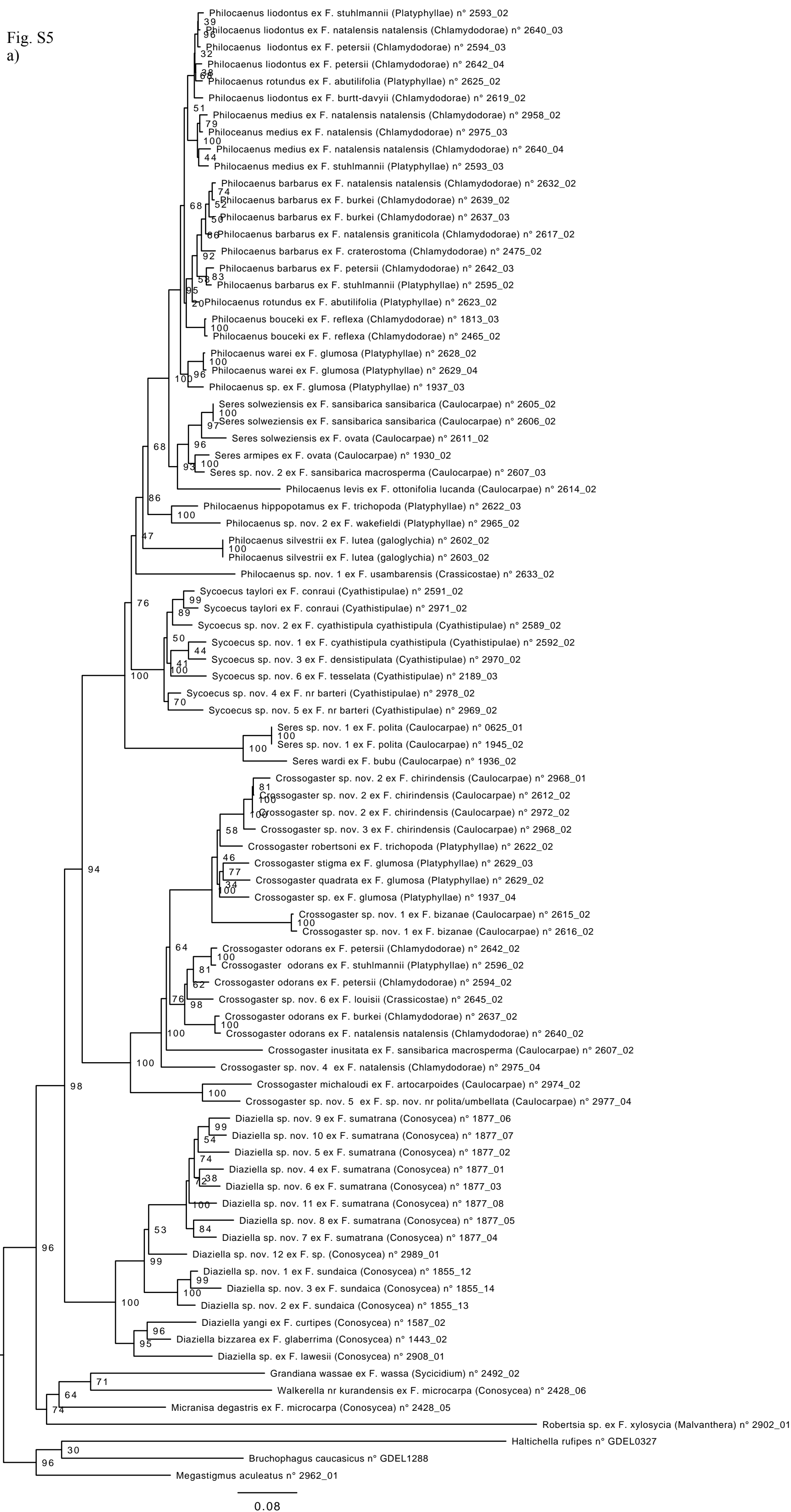

b)

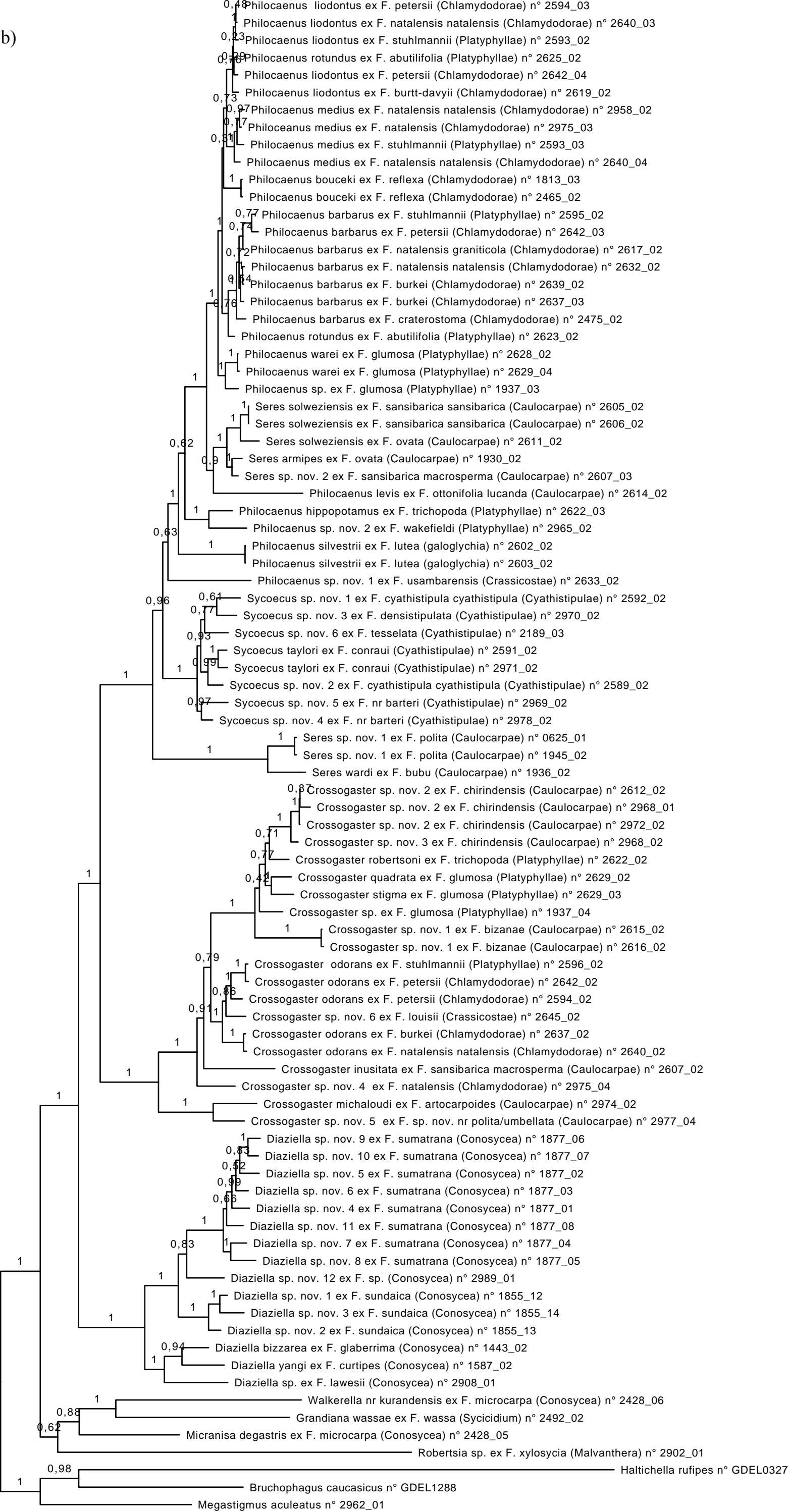

0.3

Supplement: Figure S5 — Trees from a) the ML and b) Bayesian analyses of the combined dataset aligned using ClustalW + Gblocks (relaxed parameters) and 5 partitions. Likelihood bootstrap values and Posterior probabilities are indicated at nodes. (PDF) [file pone.0079291.s009.pdf]

Fig S6  
a)

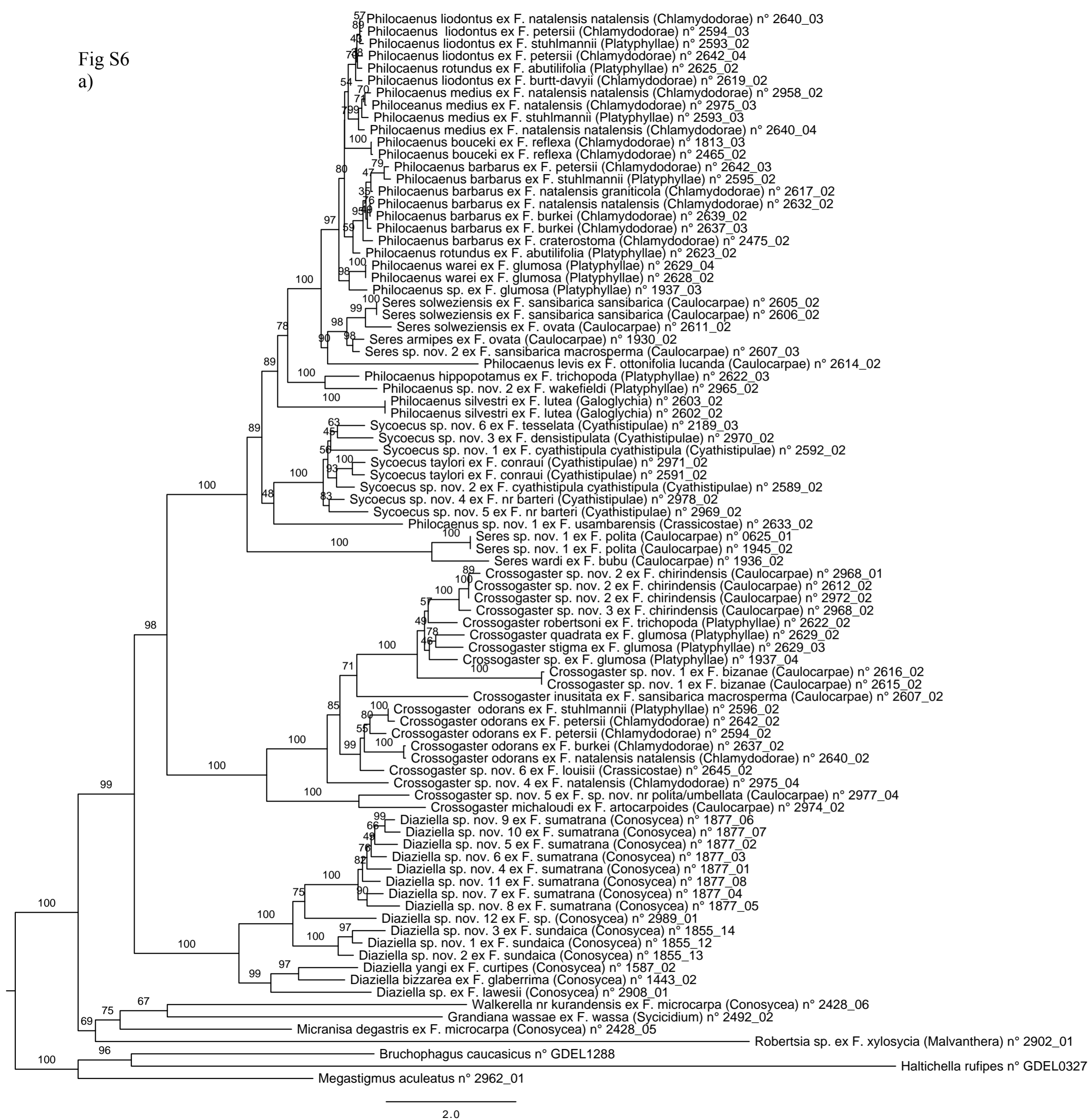

b)

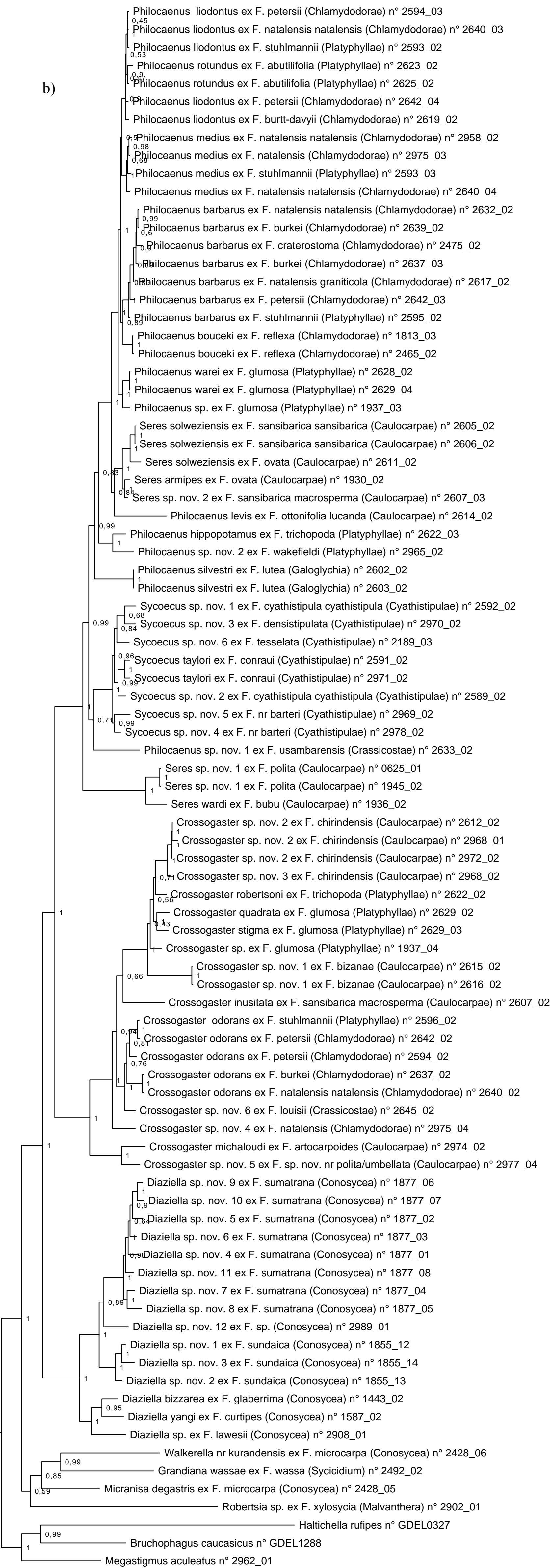

0.3

Supplement: Figure S6 — Trees from a) the ML and b) Bayesian analyses of the combined dataset aligned using ClustalW + Gblocks (relaxed parameters) and 6 partitions. Likelihood bootstrap values and Posterior probabilities are indicated at nodes. (PDF) [file pone.0079291.s010.pdf]

Fig. S7  
a)

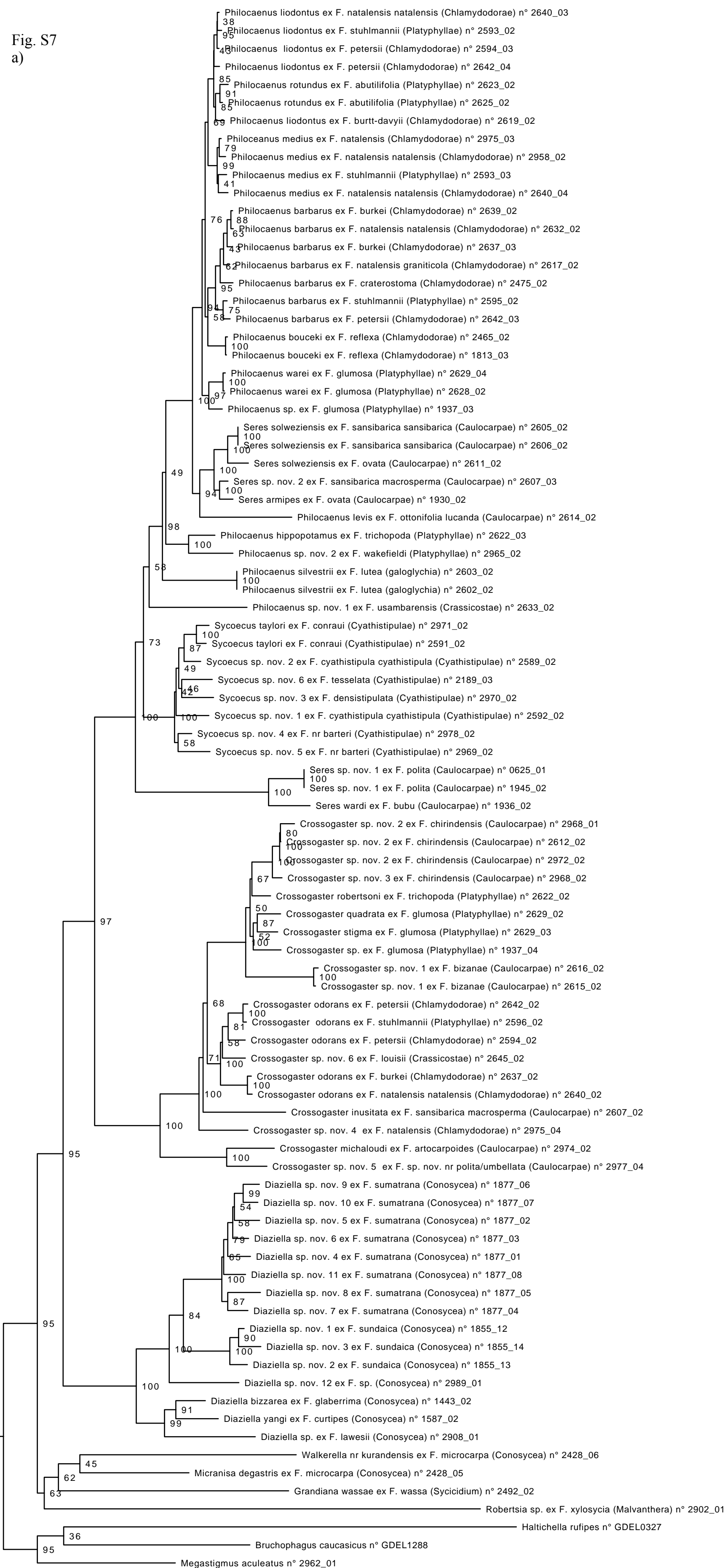

b)

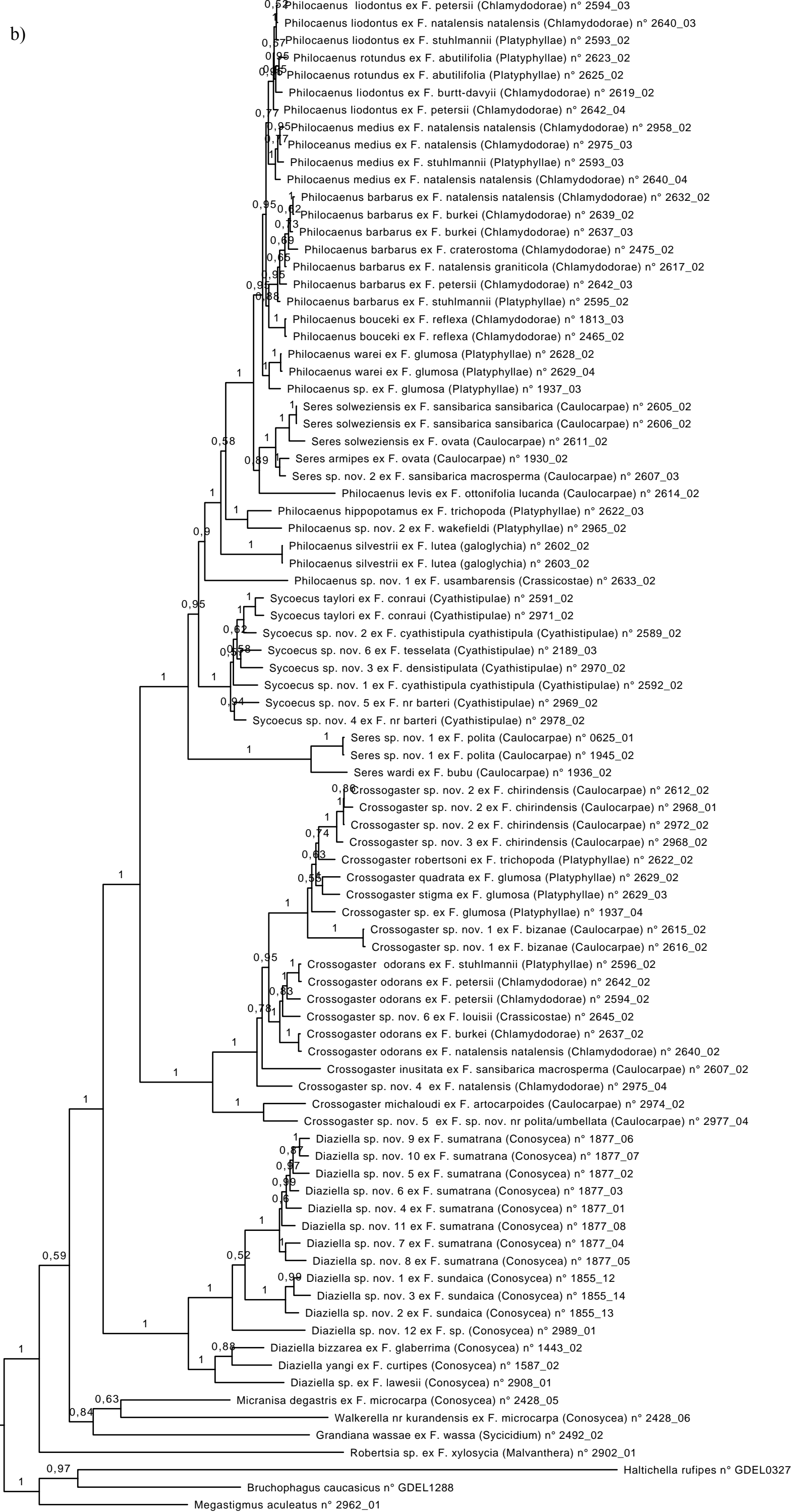

0.3

Supplement: Figure S7 — Trees from a) the ML and b) Bayesian analyses of the combined dataset aligned using MAFFT and 5 partitions. Likelihood bootstrap values and Posterior probabilities are indicated at nodes. (PDF) [file pone.0079291.s011.pdf]

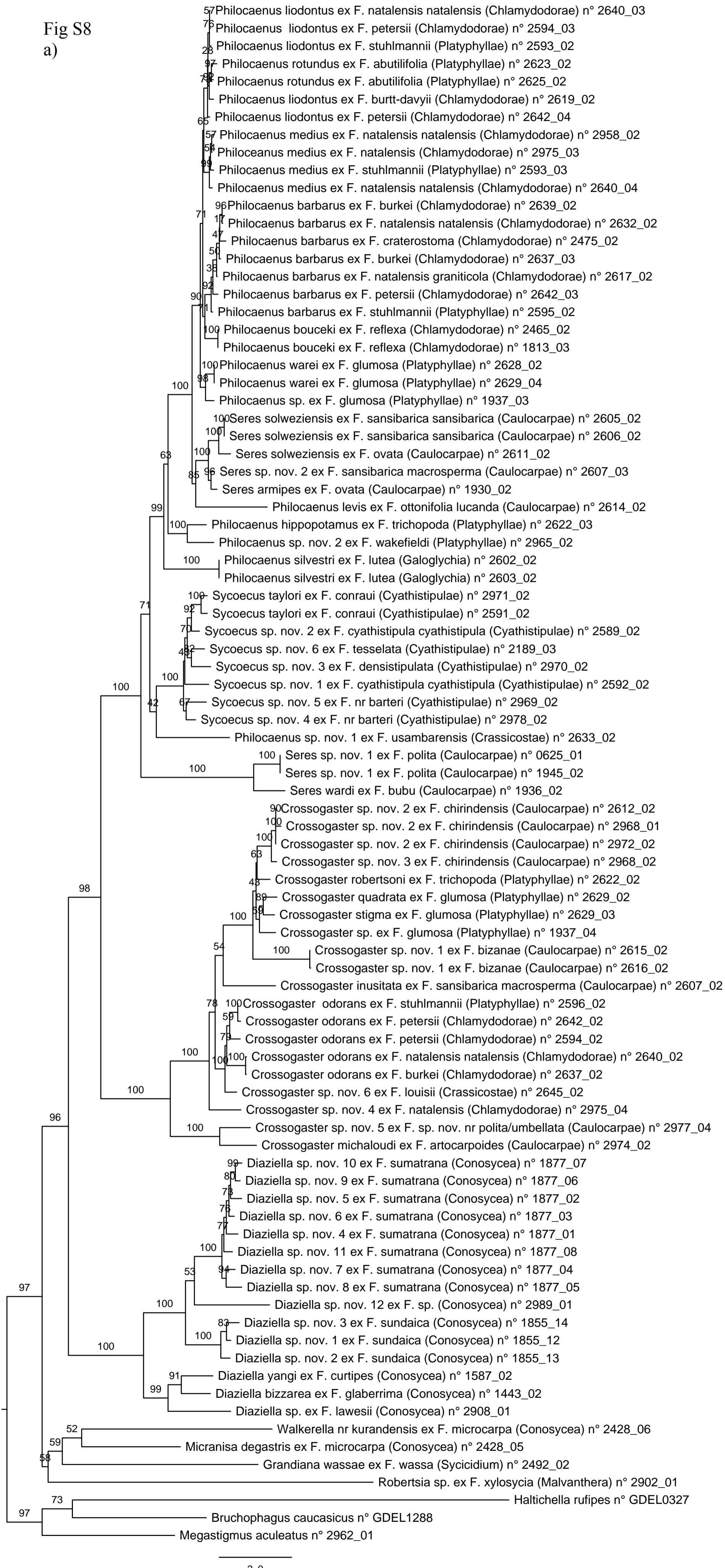

b)

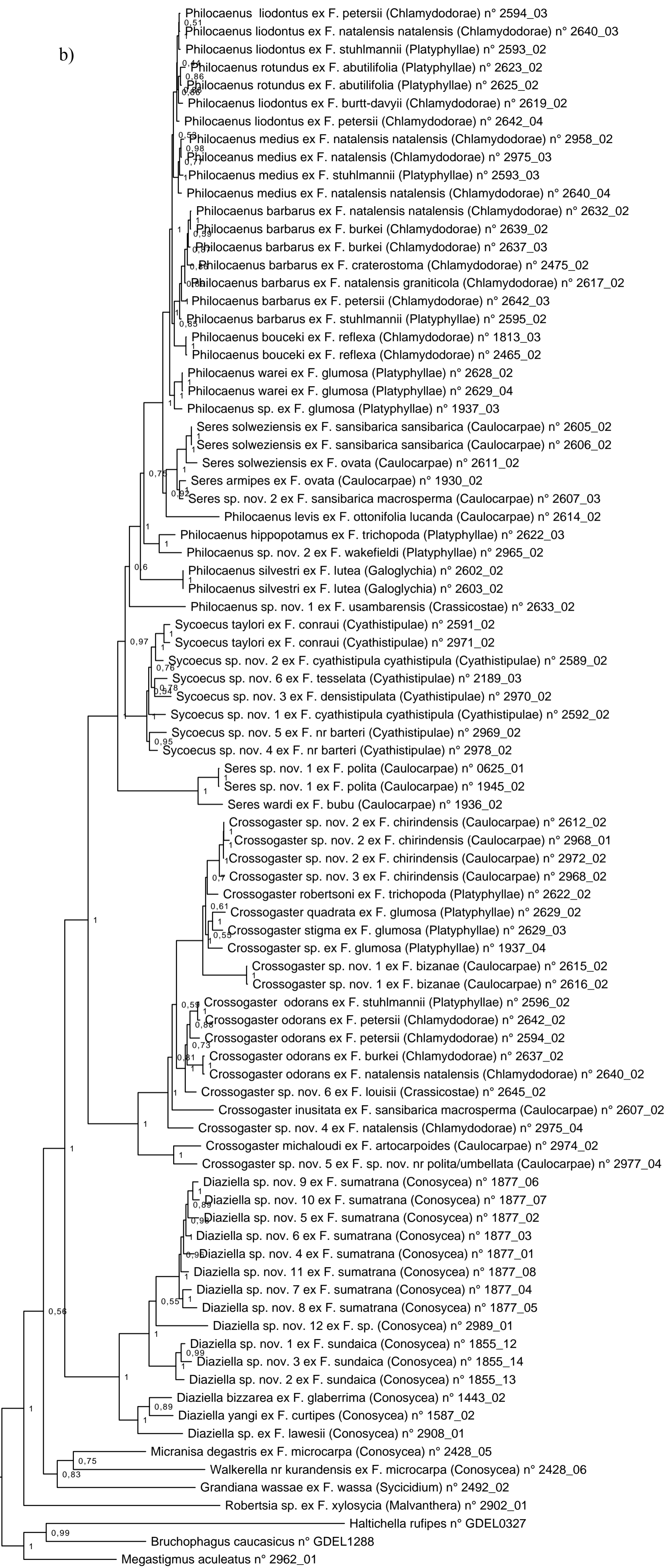

0.4

Supplement: Figure S8 — Trees from a) the ML and b) Bayesian analyses of the combined dataset aligned using MAFFT and 6 partitions. Likelihood bootstrap values and Posterior probabilities are indicated at nodes. (PDF) [file pone.0079291.s012.pdf]

Fig. S9  
a)

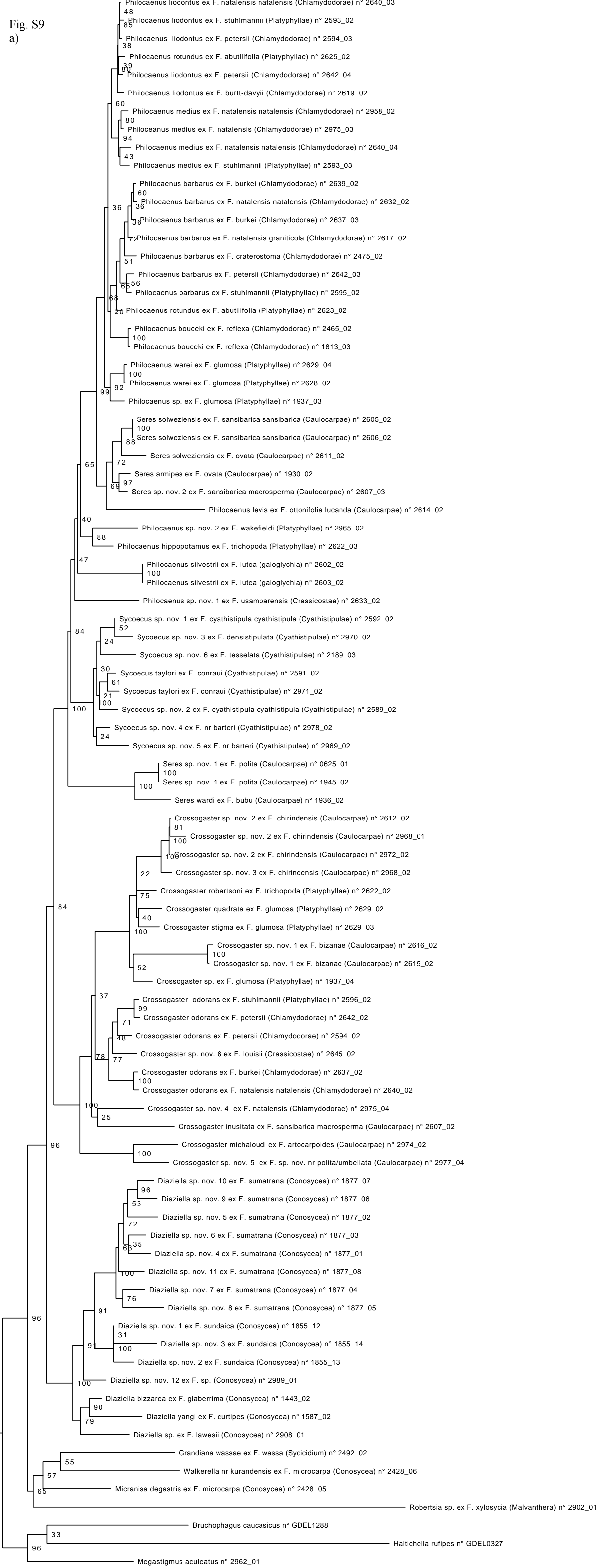

b)

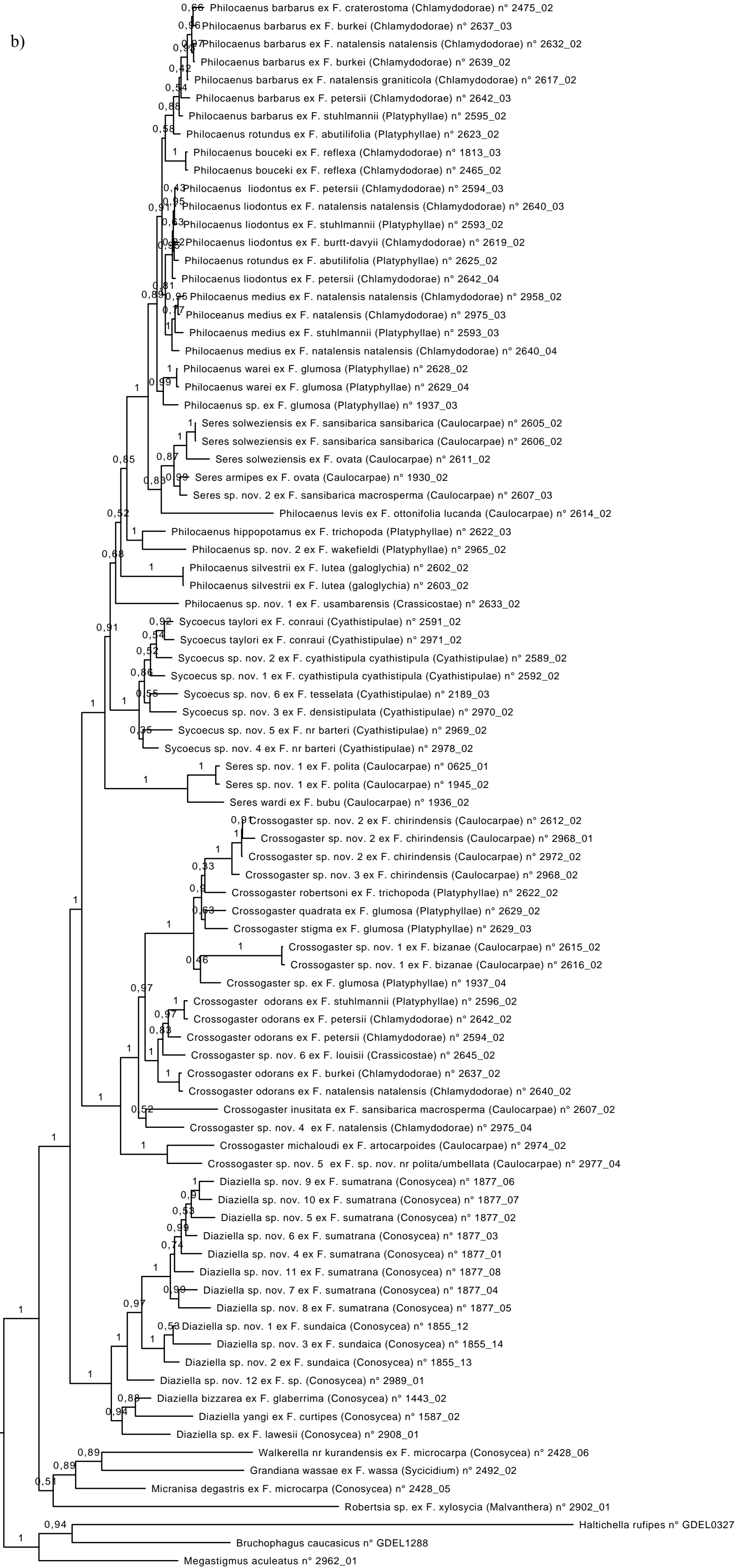

0.3

Supplement: Figure S9 — Trees from a) the ML and b) Bayesian analyses of the combined dataset aligned using MAFFT + Gblocks (default parameters) and 5 partitions. Likelihood bootstrap values and Posterior probabilities are indicated at nodes. (PDF) [file pone.0079291.s013.pdf]

Fig S10  
a)

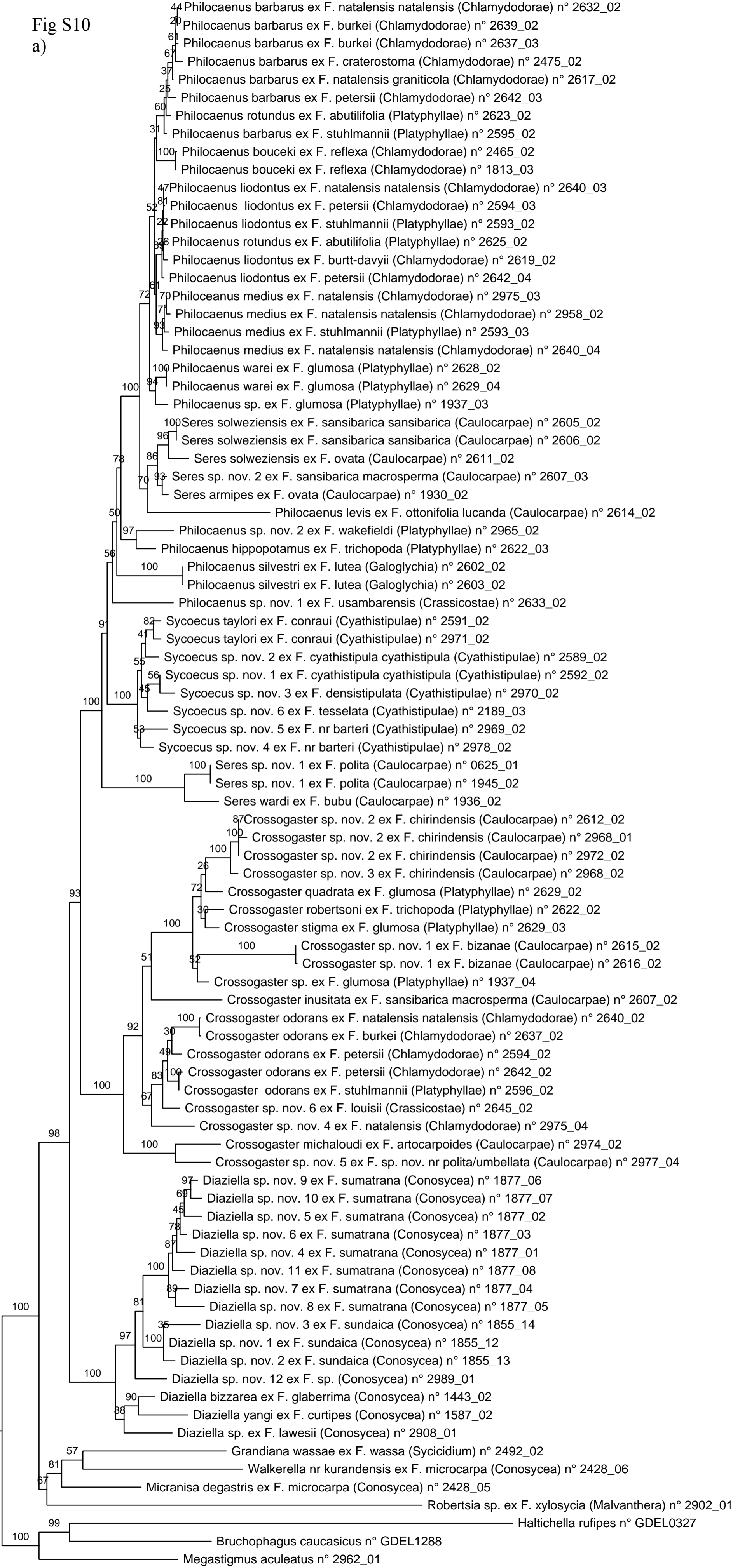

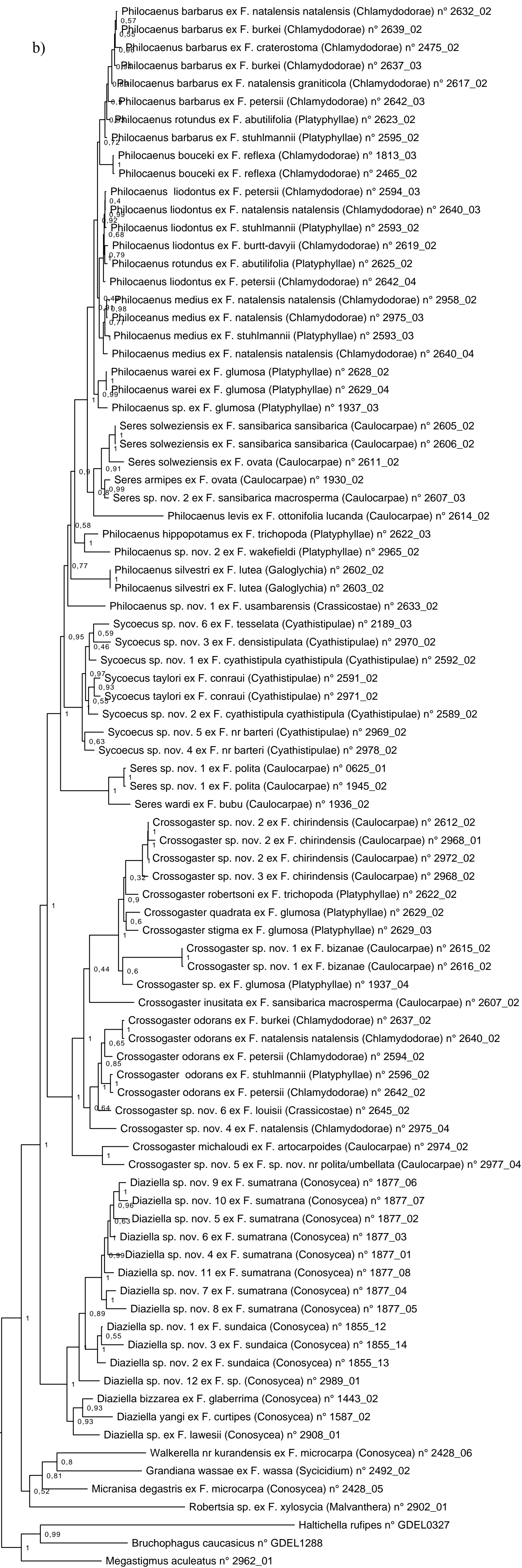

Supplement: Figure S10 — Trees from a) the ML and b) Bayesian analyses of the combined dataset aligned using MAFFT + Gblocks (default parameters) and 6 partitions. Likelihood bootstrap values and Posterior probabilities are indicated at nodes. (PDF) [file pone.0079291.s014.pdf]

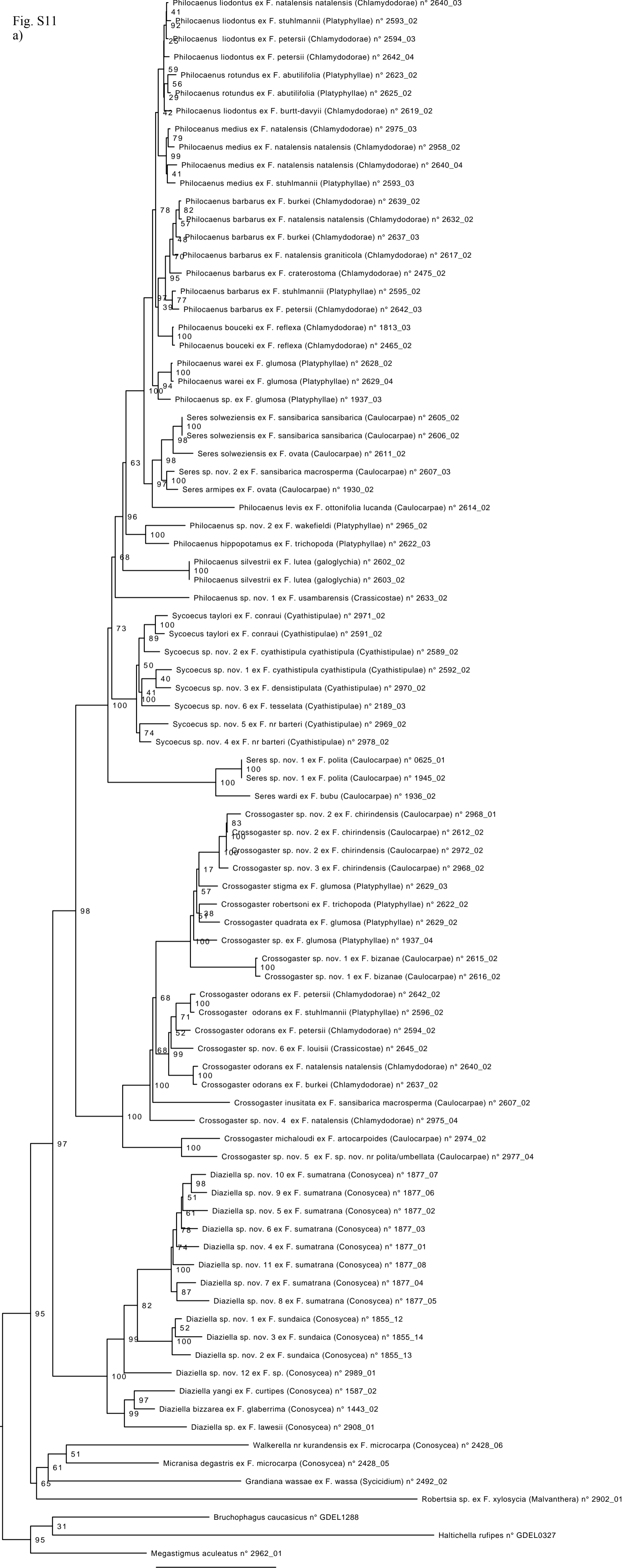

0.2

b)

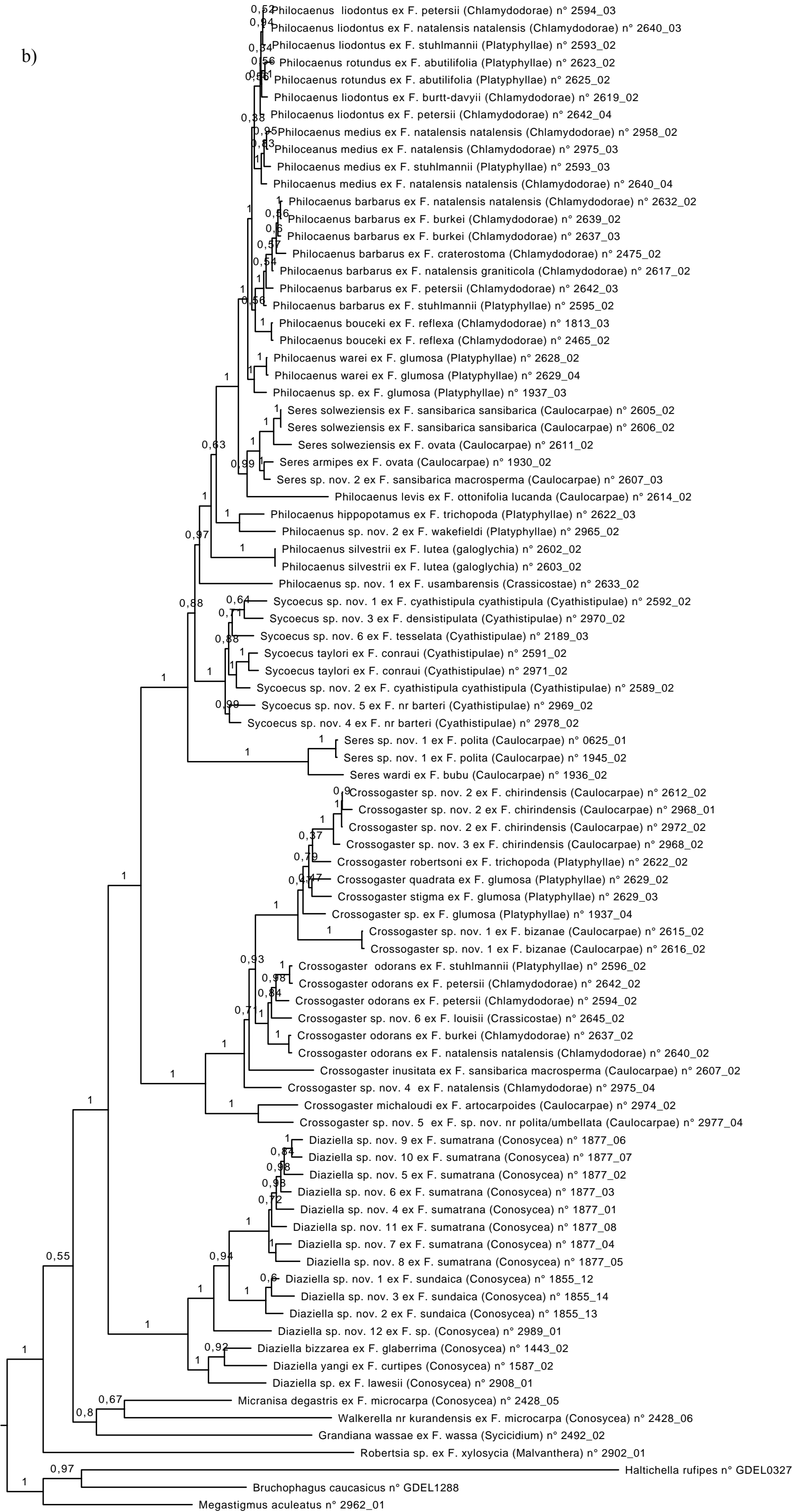

Supplement: Figure S11 — Trees from a) the ML and b) Bayesian analyses of the combined dataset aligned using MAFFT + Gblocks (relaxed parameters) and 5 partitions. Likelihood bootstrap values and Posterior probabilities are indicated at nodes. (PDF) [file pone.0079291.s015.pdf]

Fig S12  
a)

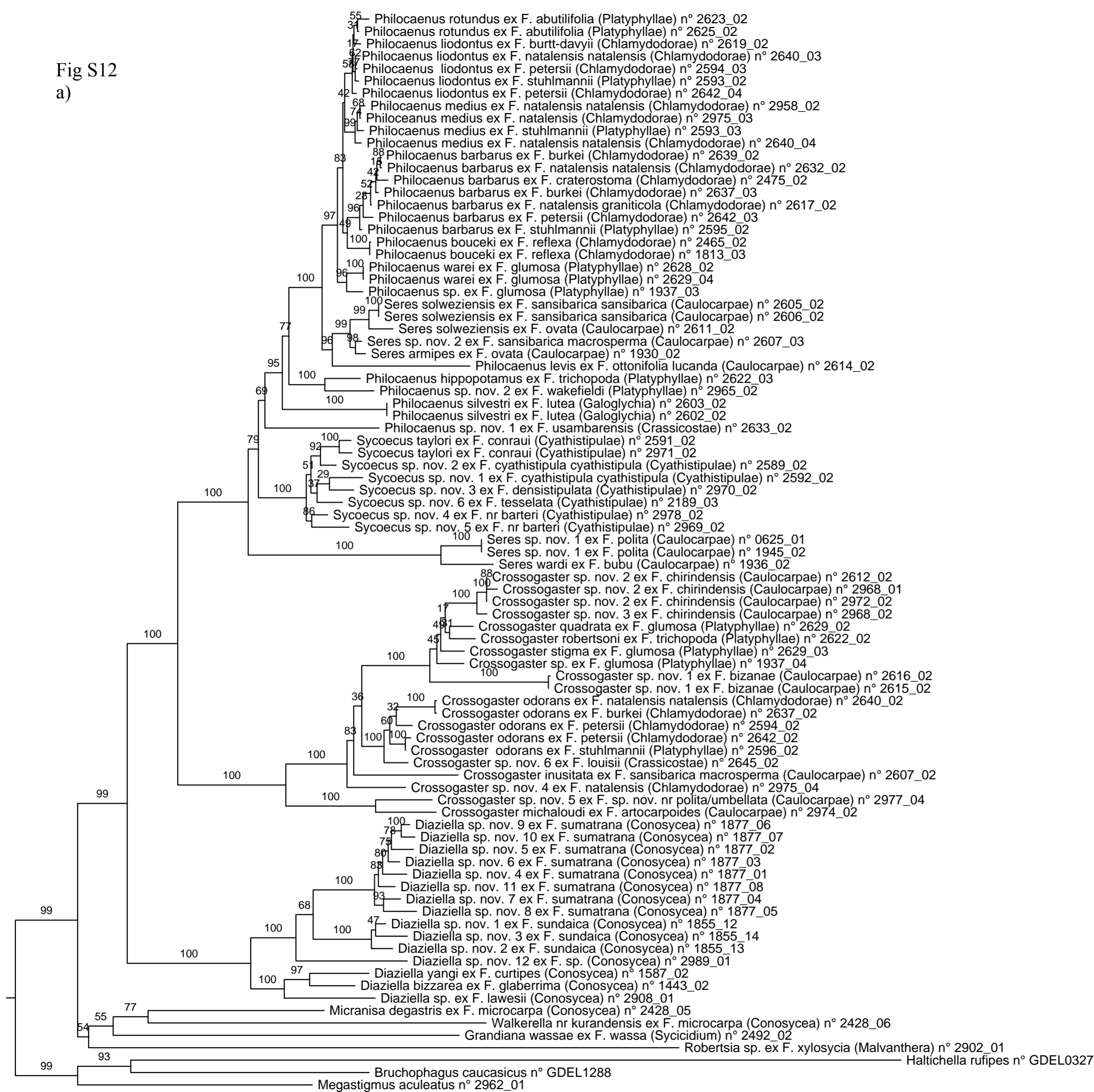

b)

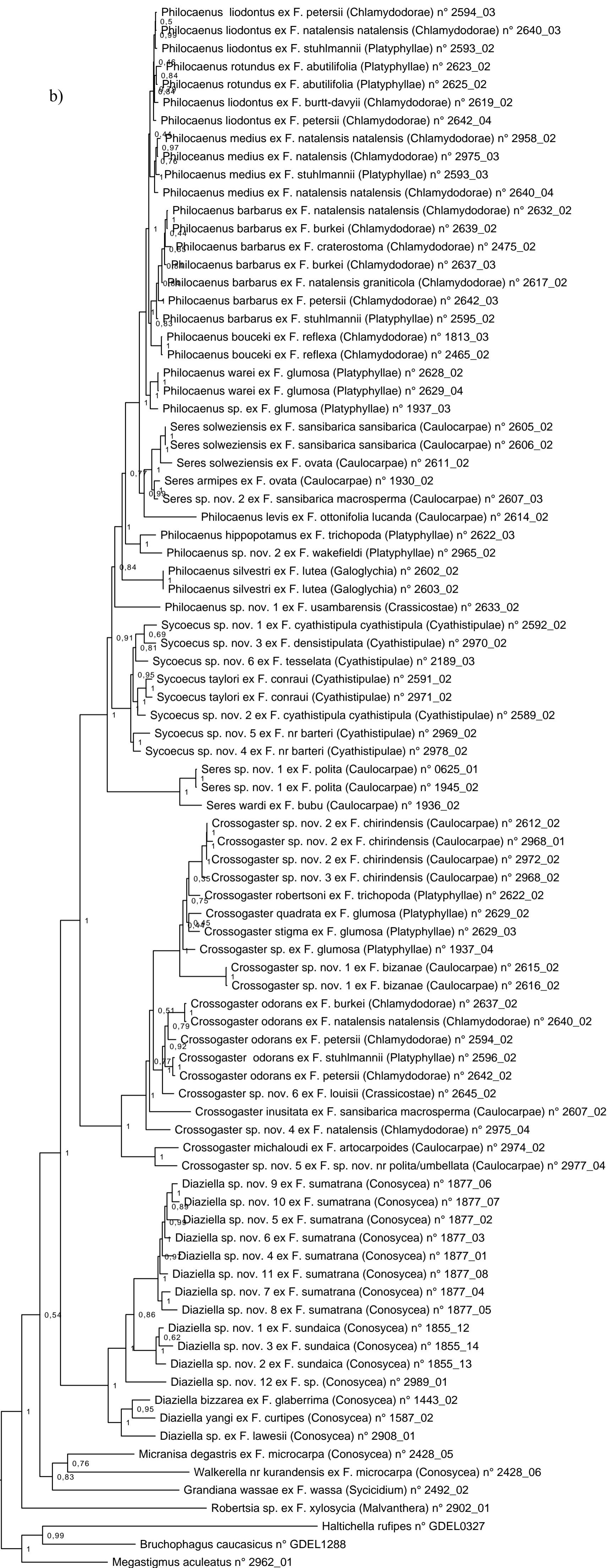

0.3

Supplement: Figure S12 — Trees from a) the ML and b) Bayesian analyses of the combined dataset aligned using MAFFT + Gblocks (relaxed parameters) and 6 partitions. Likelihood bootstrap values and Posterior probabilities are indicated at nodes. (PDF) [file pone.0079291.s016.pdf]

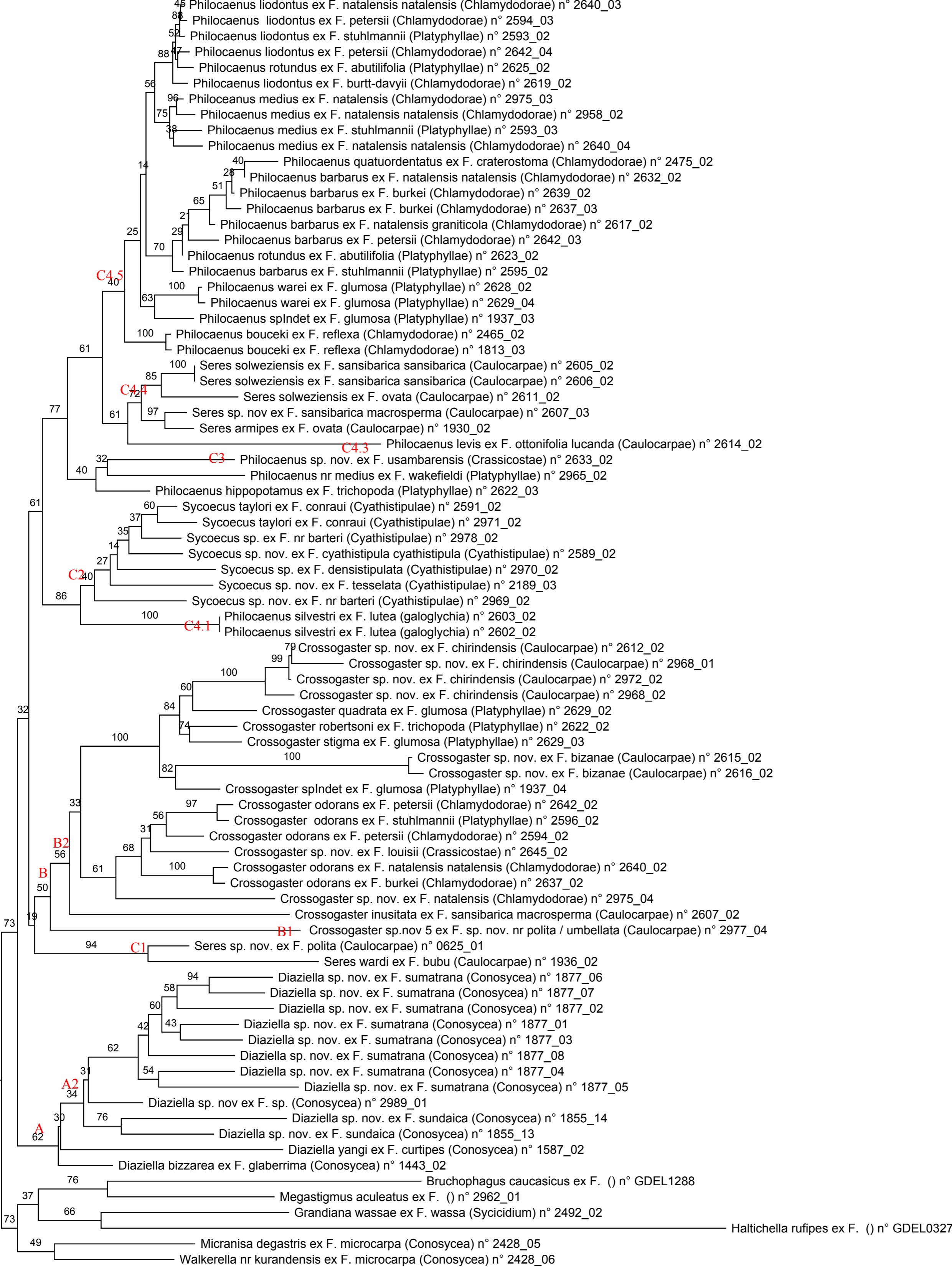

0.07

Supplement: Figure S13 — Tree from the ML analysis of the mitochondrial partition. Likelihood bootstrap values are indicated at nodes (1000 replicates). (PDF) [file pone.0079291.s017.pdf]

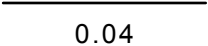

Supplement: Figure S14 — Tree from the ML analysis of the EF-1α gene region. Likelihood bootstrap values are indicated at nodes (1000 replicates). (PDF) [file pone.0079291.s018.pdf]

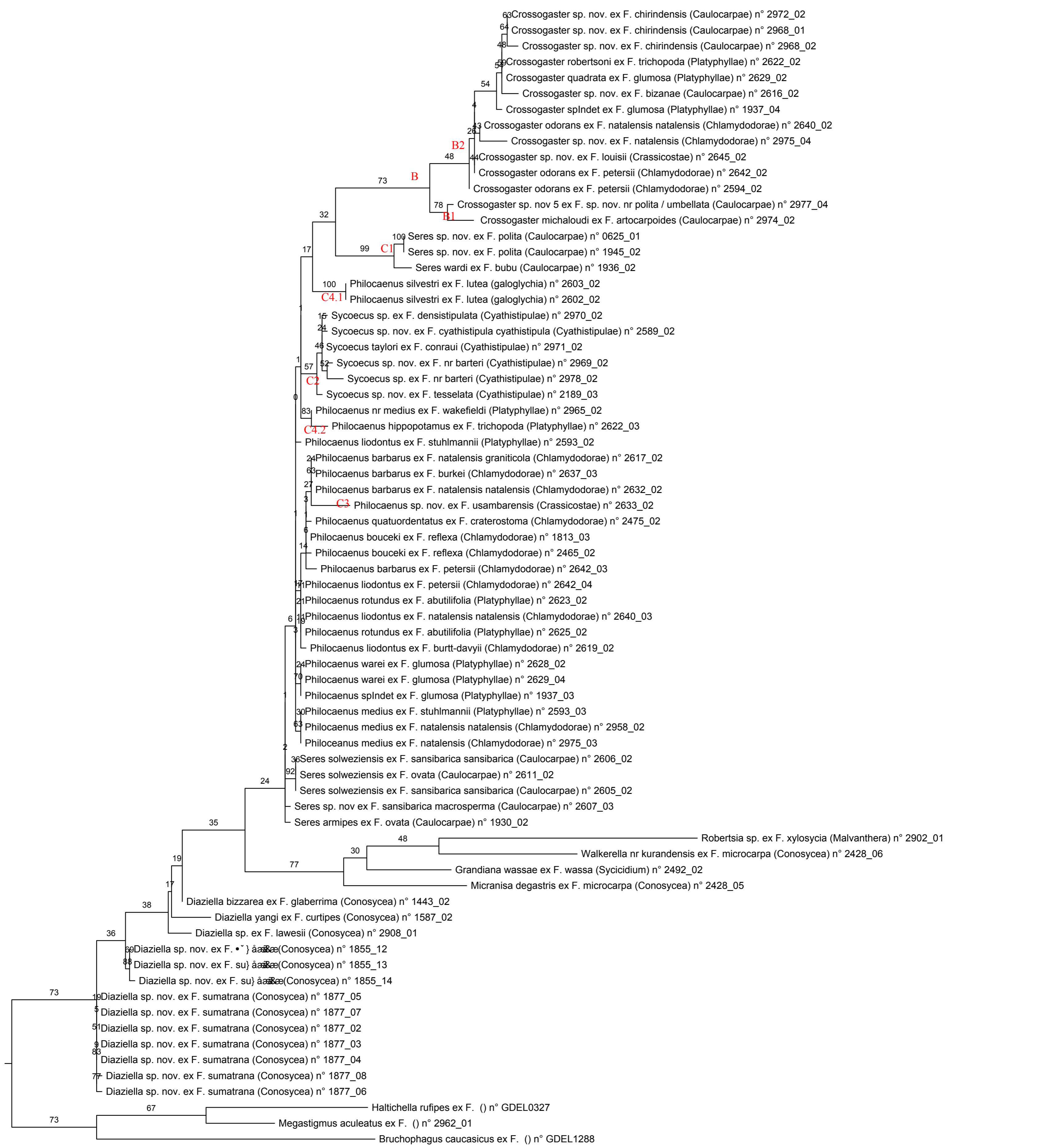

0.05

Supplement: Figure S15 — Tree from the ML analysis of the magonashi gene region. Likelihood bootstrap values are indicated at nodes (1000 replicates). (PDF) [file pone.0079291.s019.pdf]

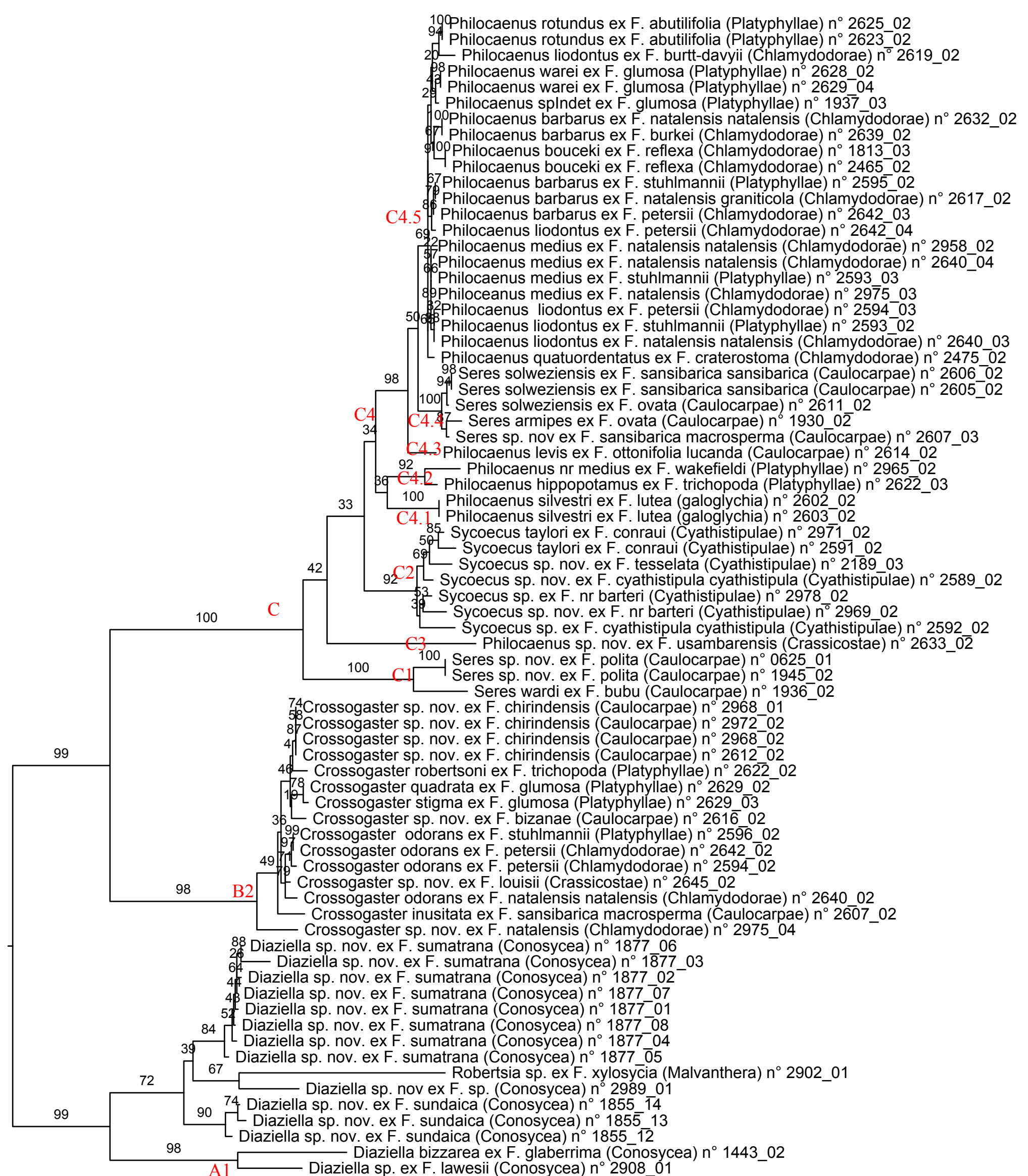

Supplement: Figure S16 — Tree from the ML analysis of the ITS2 gene region (ClustalW alignment). Likelihood bootstrap values are indicated at nodes (1000 replicates). (PDF) [file pone.0079291.s020.pdf]

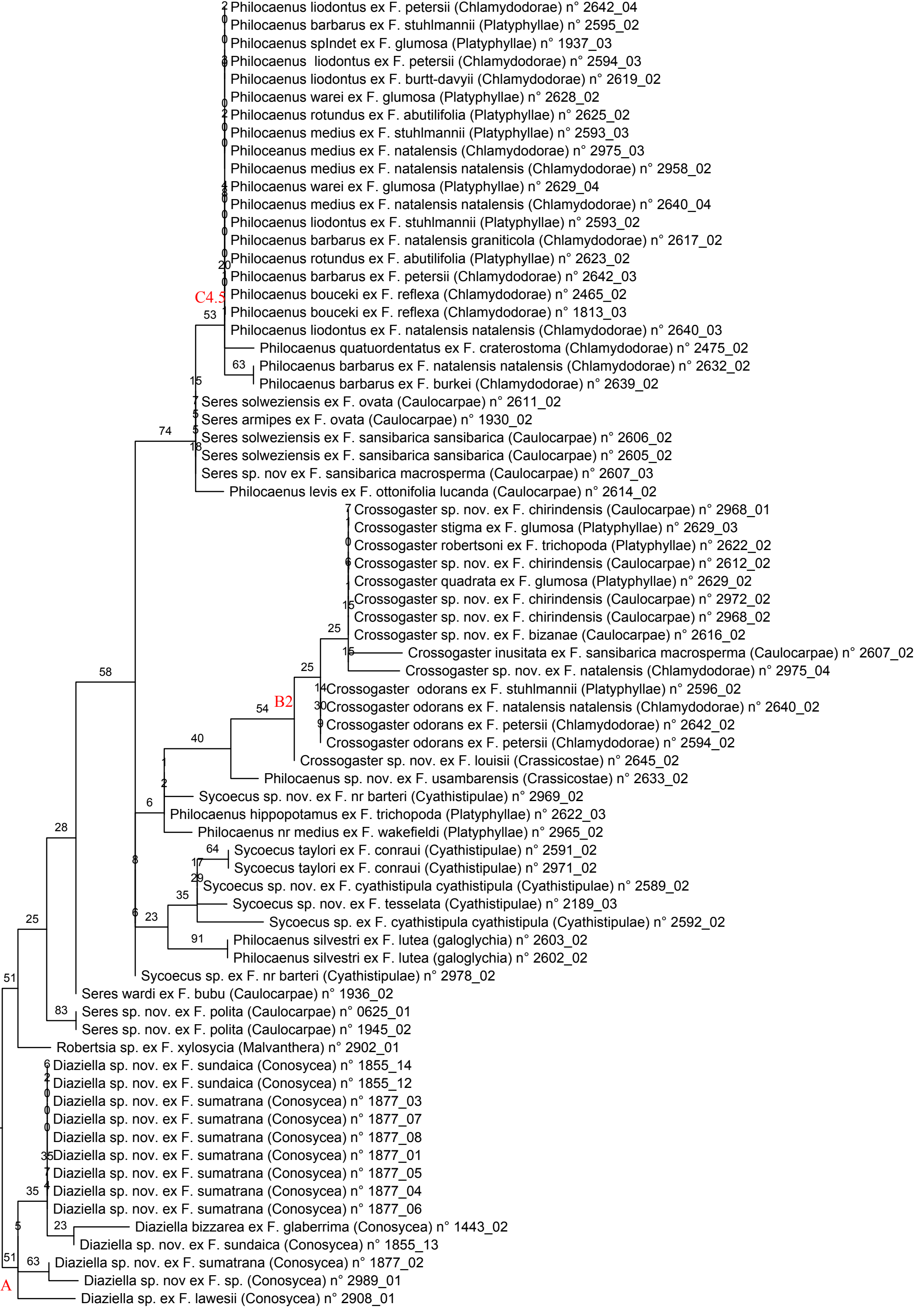

A

0.03

Supplement: Figure S17 — Tree from the ML analysis of the ITS2 gene region (ClustalW alignment + Gblocks default parameters). Likelihood bootstrap values are indicated at nodes (1000 replicates). (PDF) [file pone.0079291.s021.pdf]

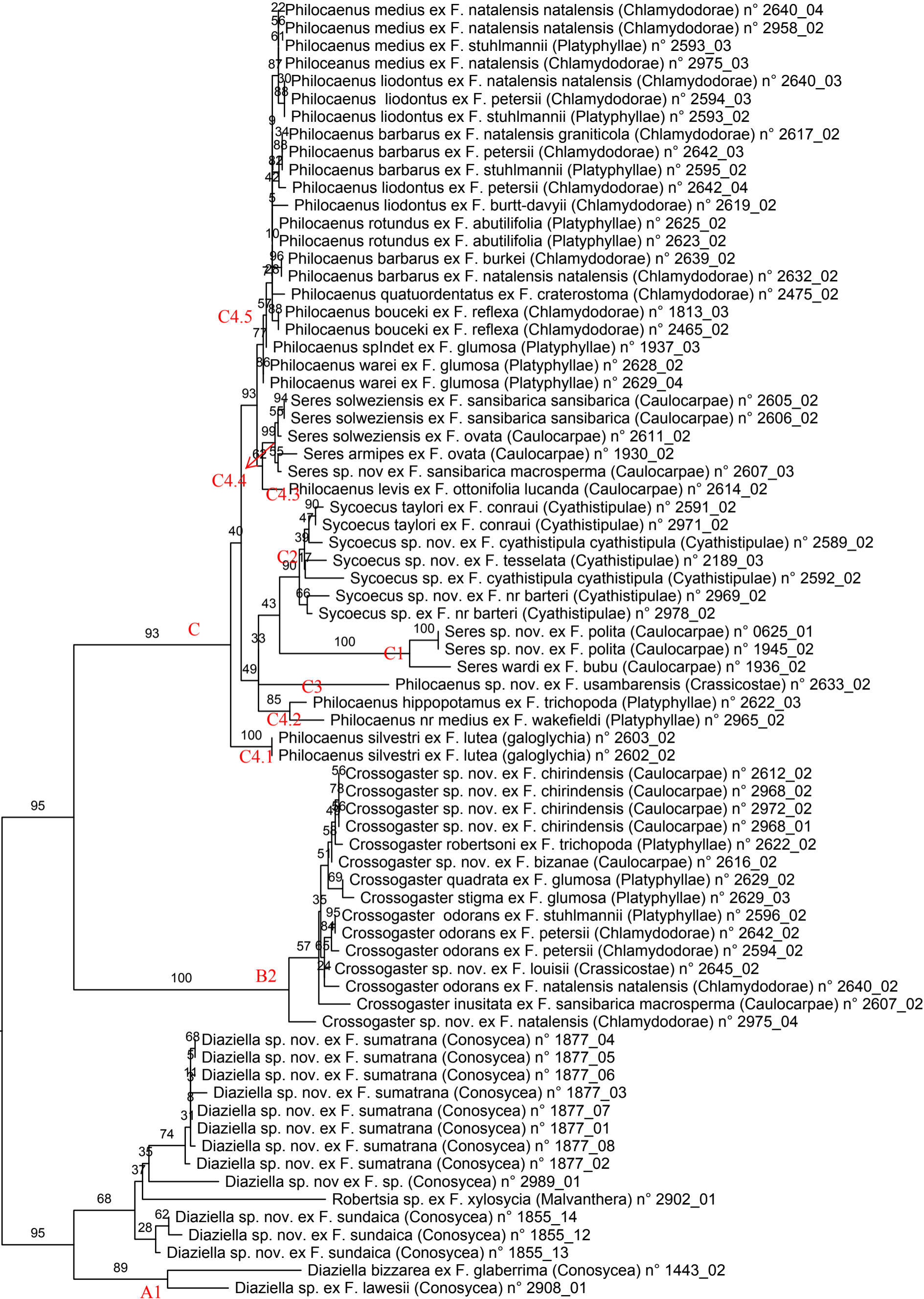

0.05

Supplement: Figure S18 — Tree from the ML analysis of the ITS2 gene region (ClustalW alignment + Gblocks relaxed parameters). Likelihood bootstrap values are indicated at nodes (1000 replicates). (PDF) [file pone.0079291.s022.pdf]

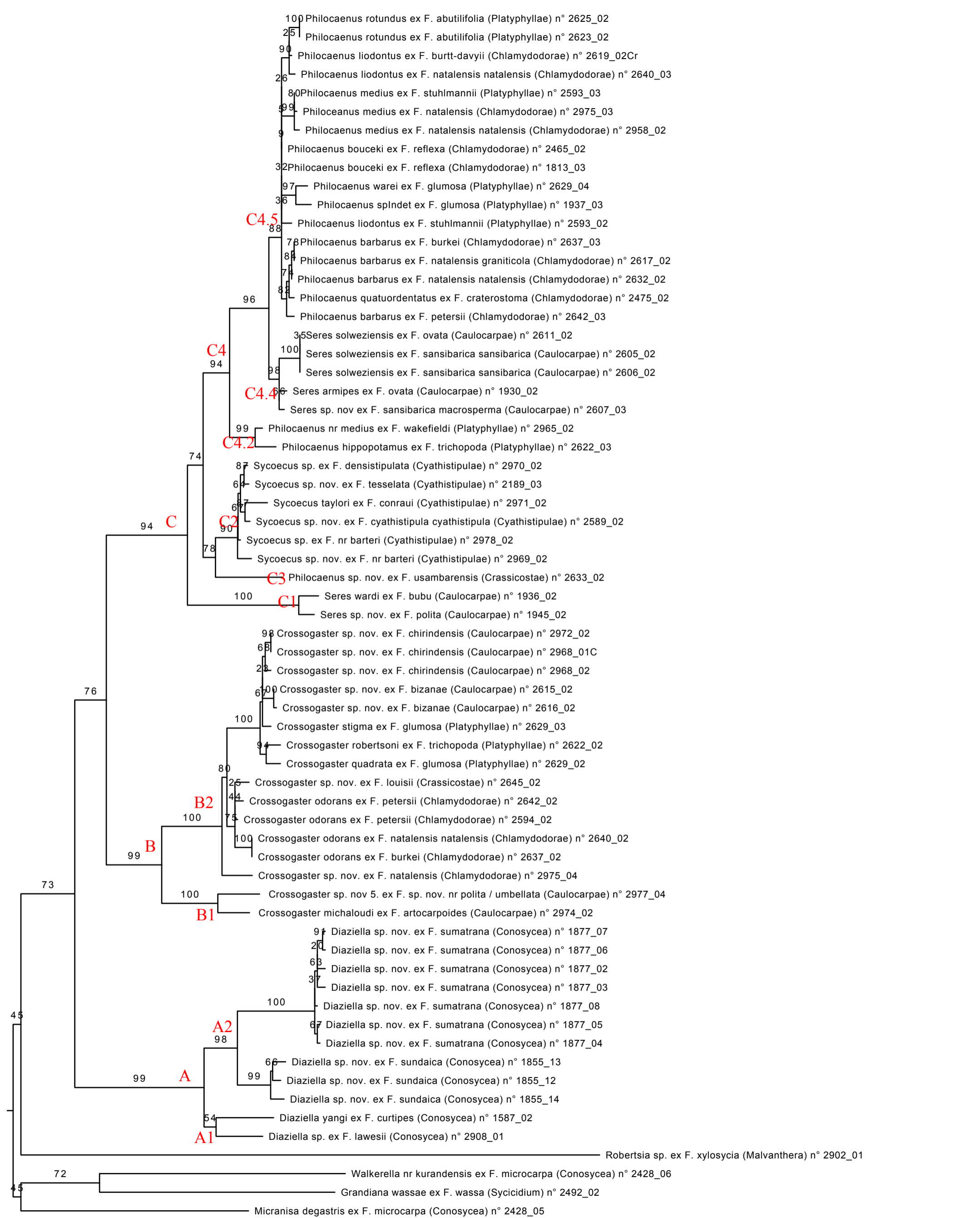

0.05

Supplement: Figure S19 — Tree from the ML analysis of the RpL27a gene region (ClustalW alignment). Likelihood bootstrap values are indicated at nodes (1000 replicates). (PDF) [file pone.0079291.s023.pdf]

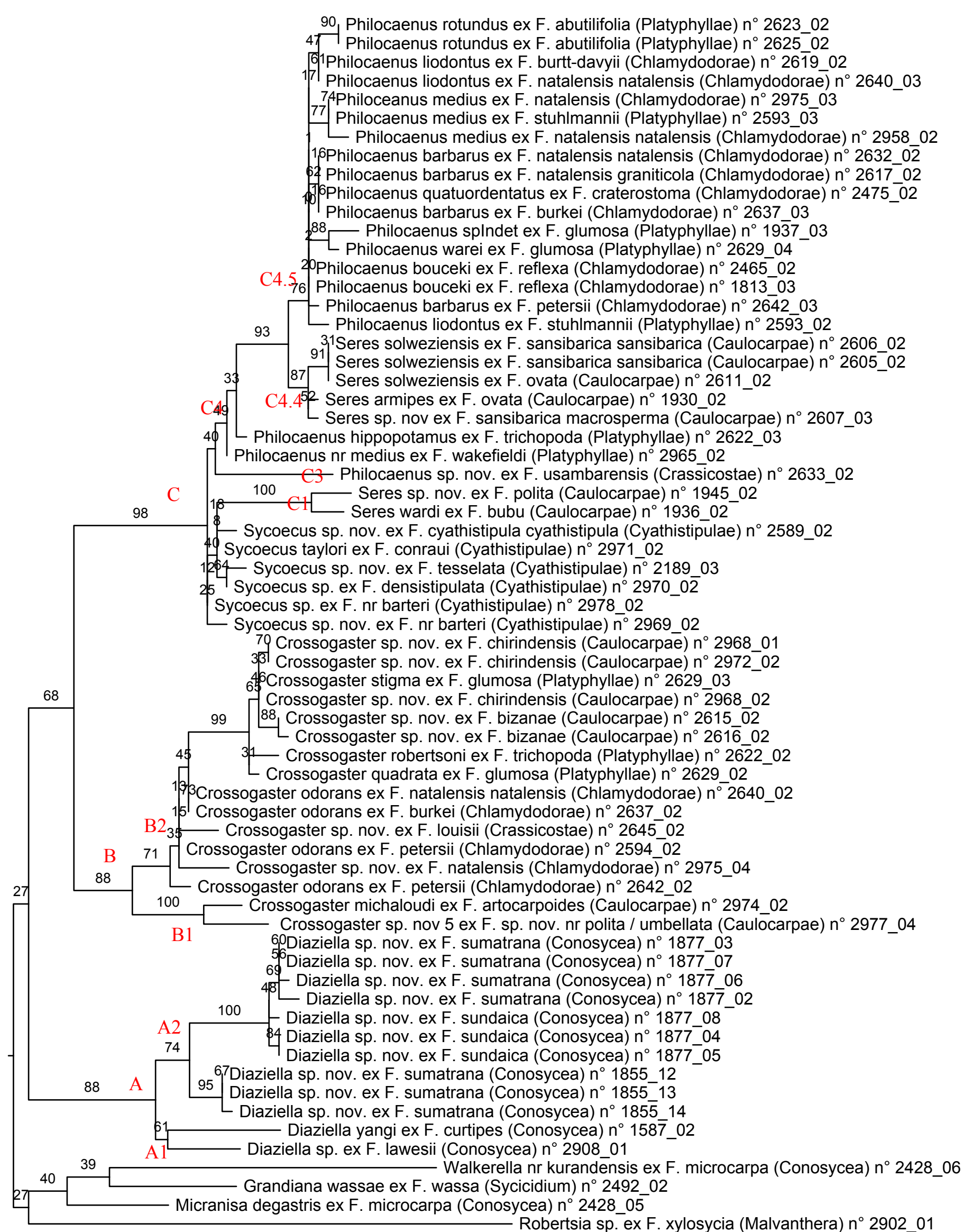

Supplement: Figure S20 — Tree from the ML analysis of the RpL27a gene region (ClustalW alignment + Gblocks default parameters). Likelihood bootstrap values are indicated at nodes (1000 replicates). (PDF) [file pone.0079291.s024.pdf]

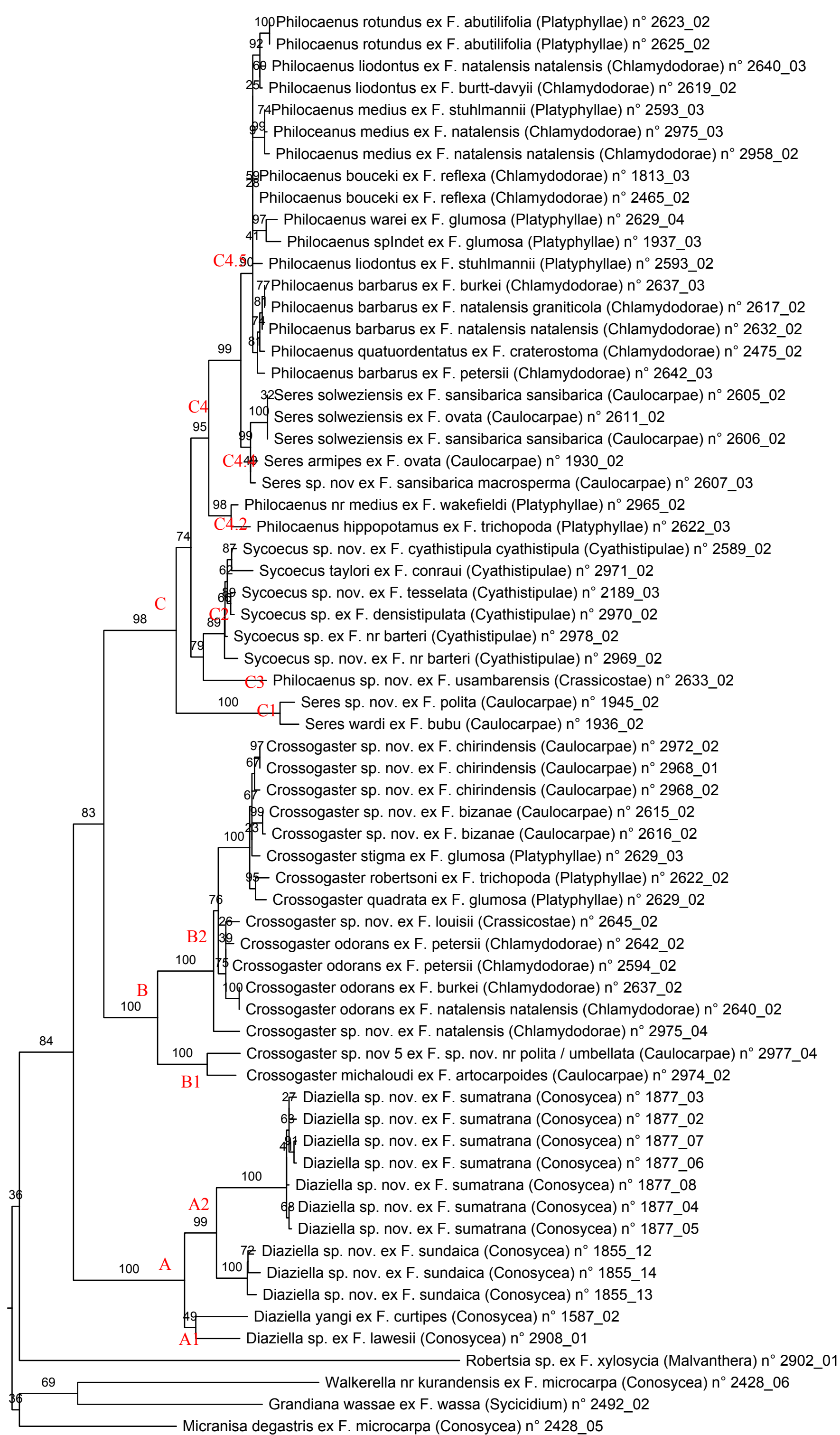

Supplement: Figure S21 — Tree from the ML analysis of the RpL27a gene region (ClustalW alignment + Gblocks relaxed parameters). Likelihood bootstrap values are indicated at nodes (1000 replicates). (PDF) [file pone.0079291.s025.pdf]

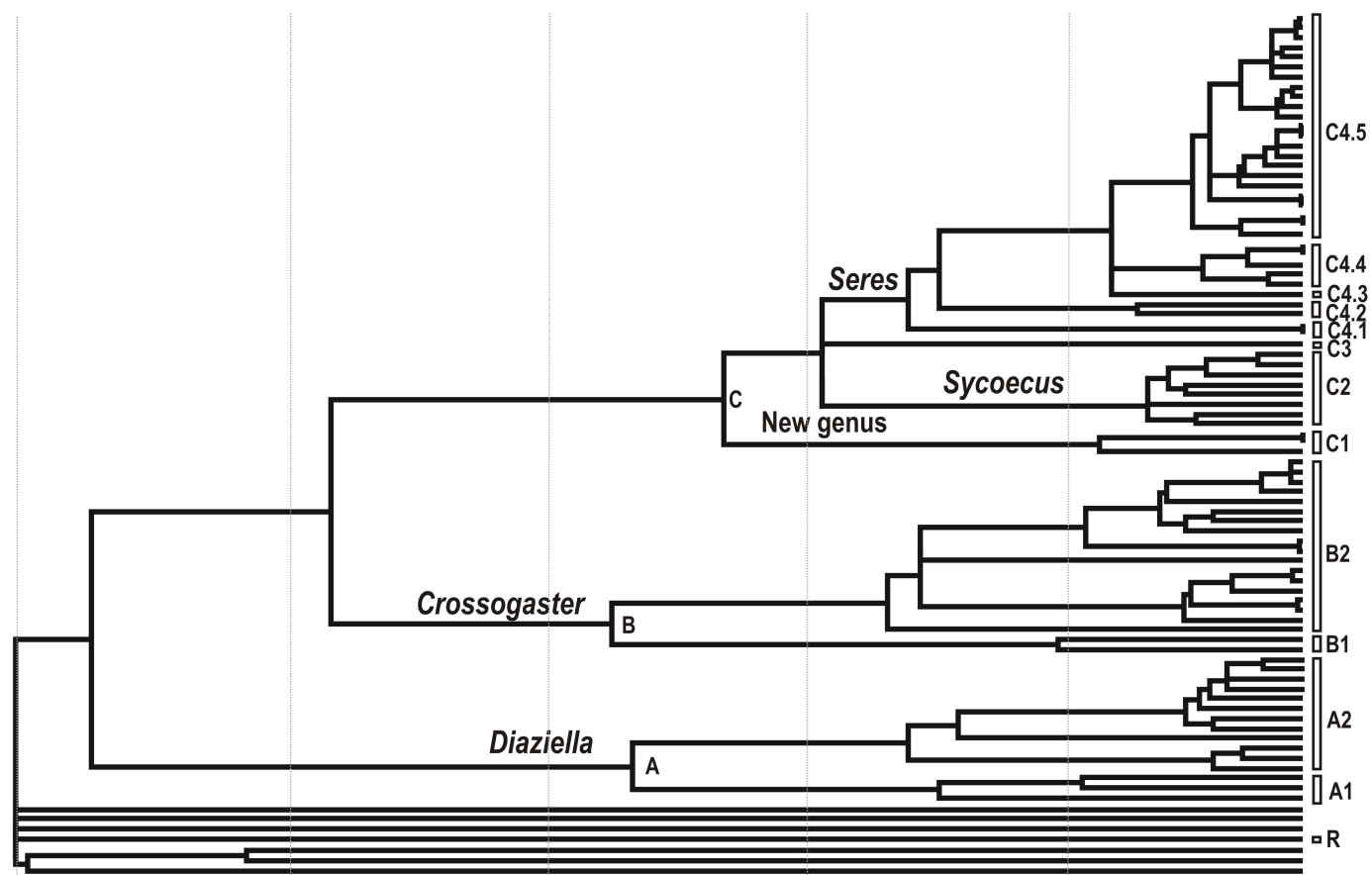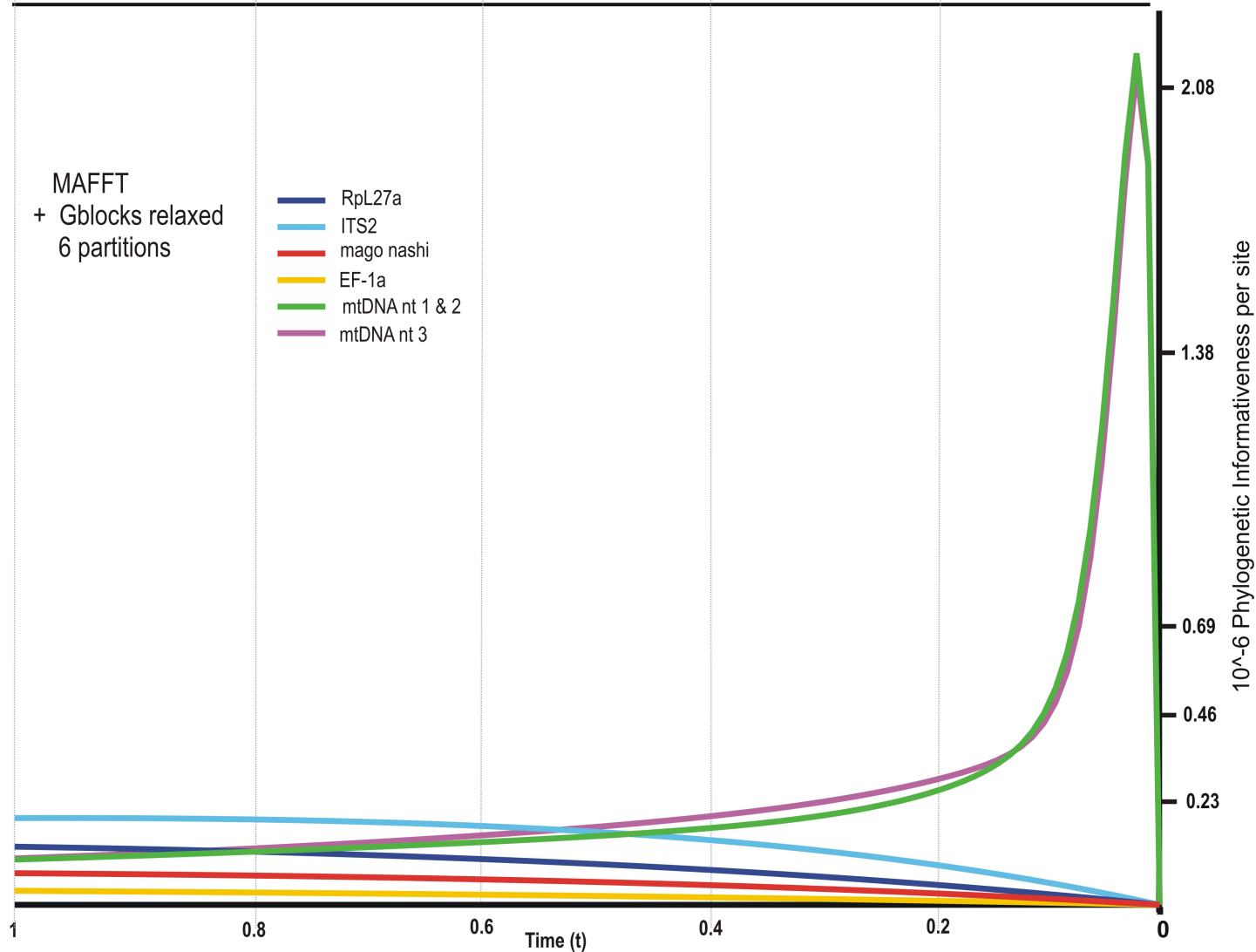

Supplement: Figure S22 — Per site phylogenetic informativeness profiles of the markers based on the MAFFT + Gblocks relaxed parameters dataset. Uppercase letters refer to clades discussed in the text (see also Figures 1 & 2). (PDF) [file pone.0079291.s026.pdf]

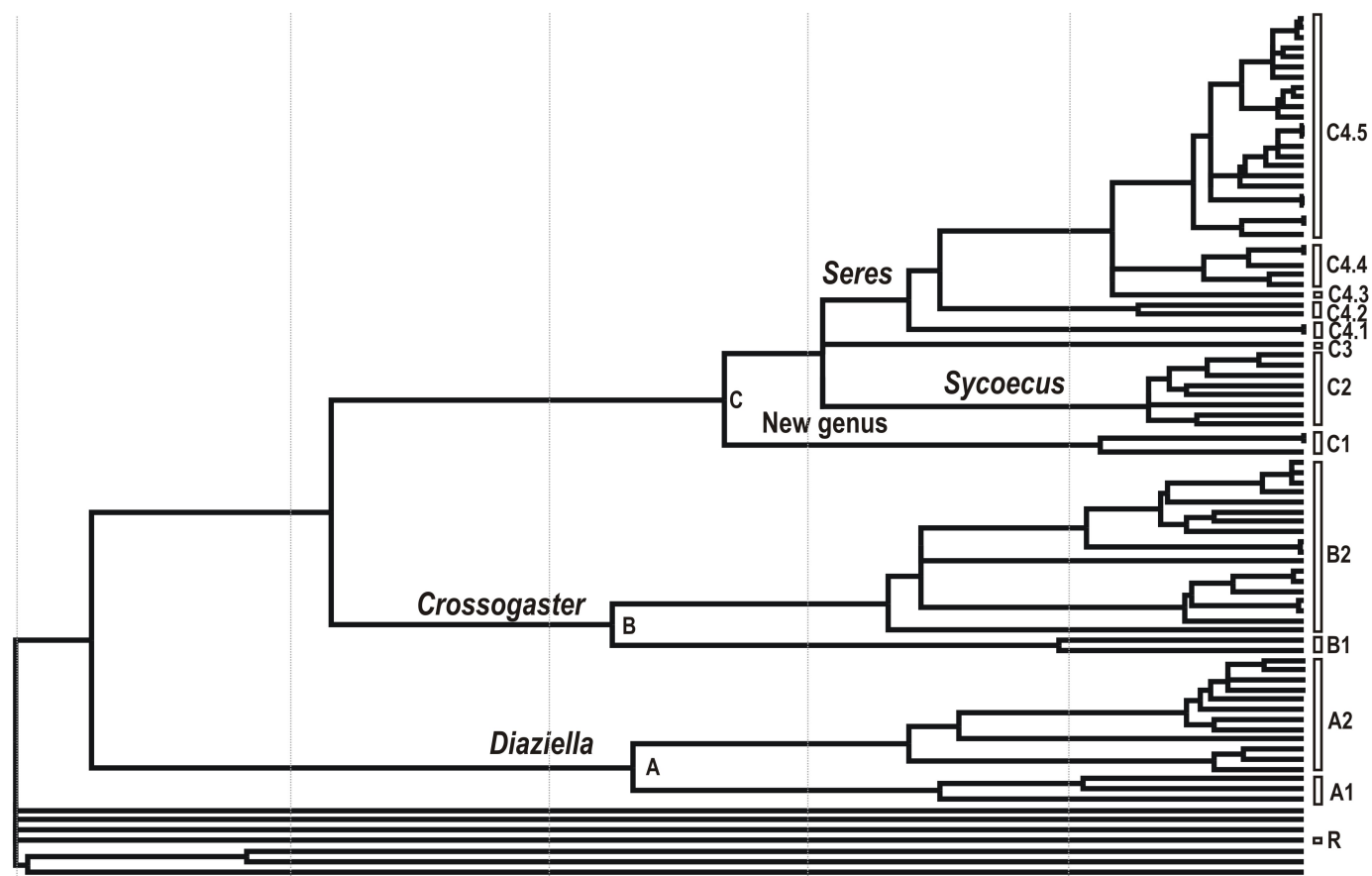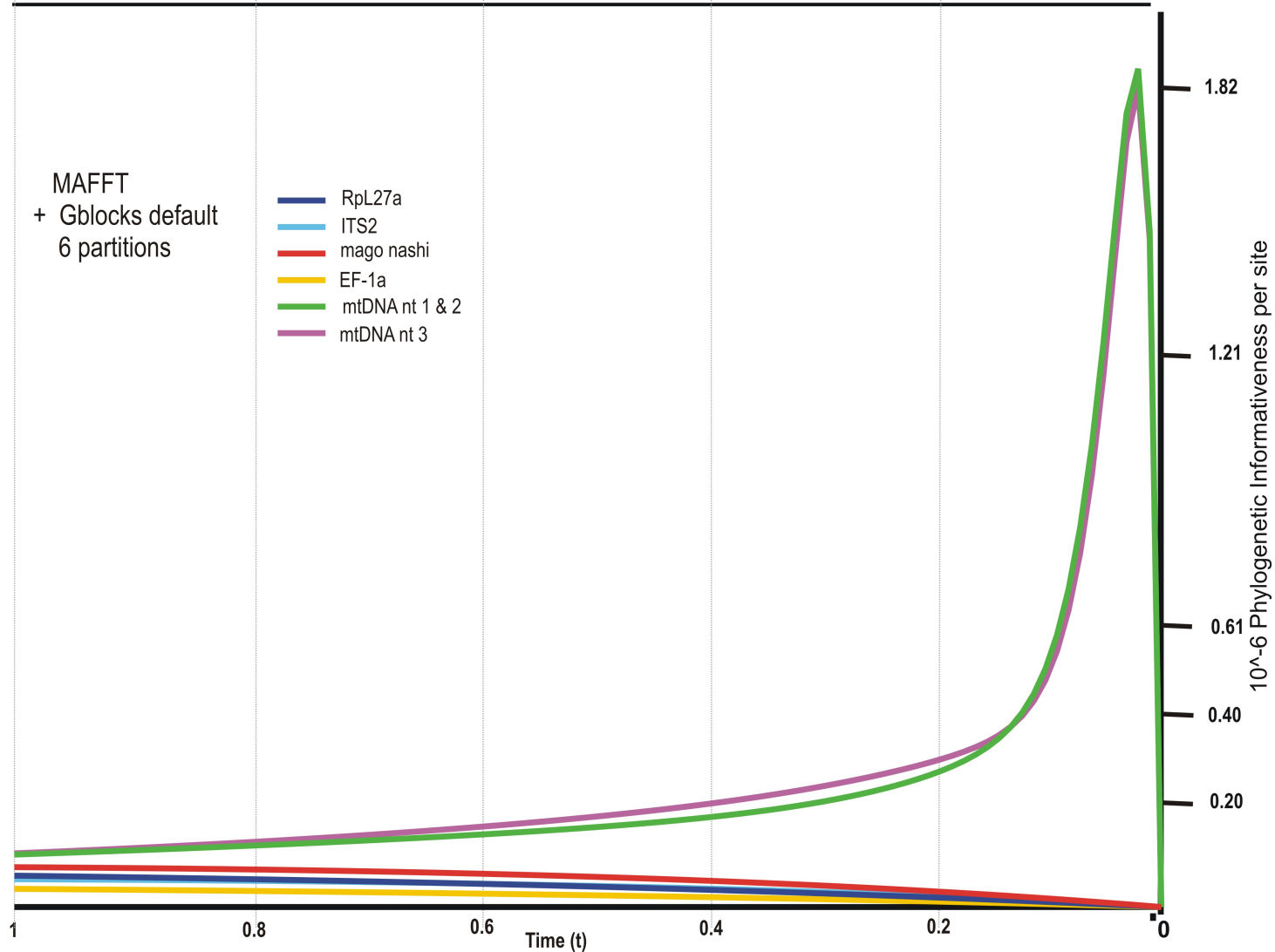

Supplement: Figure S23 — Per site phylogenetic informativeness profiles of the markers based on the MAFFT + Gblocks default parameters dataset. Uppercase letters refer to clades discussed in the text (see also Figures 1 & 2). (PDF) [file pone.0079291.s027.pdf]
